# Arctic soil methane sink increases with drier conditions and higher ecosystem respiration

**DOI:** 10.1038/s41558-023-01785-3

**Published:** 2023-08-31

**Authors:** Carolina Voigt, Anna-Maria Virkkala, Gabriel Hould Gosselin, Kathryn A. Bennett, T. Andrew Black, Matteo Detto, Charles Chevrier-Dion, Georg Guggenberger, Wasi Hashmi, Lukas Kohl, Dan Kou, Charlotte Marquis, Philip Marsh, Maija E. Marushchak, Zoran Nesic, Hannu Nykänen, Taija Saarela, Leopold Sauheitl, Branden Walker, Niels Weiss, Evan J. Wilcox, Oliver Sonnentag

**Affiliations:** 1 Department of Environmental and Biological Sciences, University of Eastern Finland, Kuopio, Finland; 2 Département de géographie & Centre d’études nordiques, Université de Montréal, Montréal, Quebec Canada; 3 Institute of Soil Science, Universität Hamburg, Hamburg, Germany; 4 Woodwell Climate Research Center, Falmouth, MA USA; 5 Department of Geography and Environmental Studies & Cold Regions Research Centre, Wilfrid Laurier University, Waterloo, Ontario Canada; 6 Faculty of Land and Food Systems, University of British Columbia, Vancouver, British Columbia Canada; 7 Department of Ecology and Evolutionary Biology, Princeton University, Princeton, NJ USA; 8 Institute of Soil Science, Leibniz Universität Hannover, Hannover, Germany; 9 Department of Biological and Environmental Science, University of Jyväskylä, Jyväskylä, Finland; 10 Northwest Territories Geological Survey, Yellowknife, Northwest Territories Canada

**Keywords:** Biogeochemistry, Biogeochemistry, Cryospheric science, Climate change

## Abstract

Arctic wetlands are known methane (CH_4_) emitters but recent studies suggest that the Arctic CH_4_ sink strength may be underestimated. Here we explore the capacity of well-drained Arctic soils to consume atmospheric CH_4_ using >40,000 hourly flux observations and spatially distributed flux measurements from 4 sites and 14 surface types. While consumption of atmospheric CH_4_ occurred at all sites at rates of 0.092 ± 0.011 mgCH_4_ m^−2^ h^−1^ (mean ± s.e.), CH_4_ uptake displayed distinct diel and seasonal patterns reflecting ecosystem respiration. Combining in situ flux data with laboratory investigations and a machine learning approach, we find biotic drivers to be highly important. Soil moisture outweighed temperature as an abiotic control and higher CH_4_ uptake was linked to increased availability of labile carbon. Our findings imply that soil drying and enhanced nutrient supply will promote CH_4_ uptake by Arctic soils, providing a negative feedback to global climate change.

## Abstract (web summary)

The Arctic is estimated to be a source of atmospheric methane but the sink capacity may be underestimated. This study shows that methane uptake in well-drained Arctic soils is driven by soil moisture and carbon availability, indicating a potential increased methane sink under climate change.

## Main

Soils are the only known biological sink for atmospheric methane (CH_4_), removing 11–49 TgCH_4_ from the atmosphere annually—an amount similar to CH_4_ emitted through biomass and biofuel burning^1^. The governing mechanisms of atmospheric CH_4_ consumption by soils (hereafter, CH_4_ uptake) are poorly constrained globally and especially in Arctic regions^1,2^. While estimated as a CH_4_ source, the Arctic CH_4_ budget remains uncertain (8–55 TgCH_4_ yr^−1^)^1,3^ due to the low temporal and spatial coverage of flux measurements, lack of comprehensive wetland extent datasets and limited understanding of biogeochemical processes^1,4–7^. Additionally, high-latitude wetlands are being studied intensively because they are known CH_4_ emission hot spots^4,5,8^, biasing Arctic CH_4_ inventories towards high-emitting sites^4,6,9–11^. In fact, surprisingly little attention has been paid to the capacity of well-drained Arctic soils to consume atmospheric CH_4_, although CH_4_ uptake is a common phenomenon in Arctic ecosystems^12–20^.

The Arctic is dominated by well-drained, commonly shrub- and lichen-covered uplands comprising 80% of the Arctic-boreal region^21,22^. Sedge-covered, water-saturated wetlands are located in topographic depressions and cover only 14% of the area^21,22^. Uplands and wetlands have distinct redox conditions and patterns of CH_4_ production, oxidation, gas transport and emissions^23^. While CH_4_ production and oxidation occur in both land cover types, high-affinity methanotrophs operating at atmospheric CH_4_ levels in uplands can remove CH_4_ from the atmosphere^19,24^, creating a net ecosystem CH_4_ sink. Although higher CH_4_ uptake in Arctic uplands is frequently linked to higher soil temperature^13,15,17^ stimulating methanotrophic activity, soil moisture is often a more important driver^14,15,25^, as moisture regulates air-filled pore volume and thus diffusion of atmospheric CH_4_ into soil^14,20,26,27^. Given the much larger spatial coverage of uplands, relatively small rates of CH_4_ uptake could partially compensate for carbon (C) losses to the atmosphere^15,25^.

Accurately capturing small CH_4_ fluxes in remote locations is a notable challenge due to logistical and methodological constraints. The recent development of field-deployable, high-accuracy gas analysers has made it possible to reliably measure real-time CH_4_ concentration changes of <1 ppb. Such high precision allows short (<5 min) closure times with chamber methods, preventing temperature and humidity artefacts from affecting the natural gas diffusion gradient^28,29^. Pairing high-accuracy analysers with automated chambers can generate hourly flux measurements, matching the temporal scale at which many abiotic flux drivers vary (for example, temperature, soil moisture and solar radiation). Such high-frequency measurements greatly improve upon traditional, low frequency (weekly) chamber measurements using manual air sampling and long (>30 min) closure times^29^. Importantly, high-frequency and high-accuracy flux measurements may provide insights into previously unexplored temporal dynamics (for example, night time versus daytime) of atmospheric CH_4_ uptake by Arctic soils.

Here, we investigate the temporal and spatial dynamics of Arctic soil CH_4_ uptake using high-accuracy greenhouse gas analysers and link flux patterns to microclimatic conditions and other abiotic and biotic controls. We established an automated chamber system at Trail Valley Creek (68° 44′ 32″ N; 133° 29′ 55″ W), an upland tundra site on continuous permafrost (−8.2 °C mean annual air temperature) in the western Canadian Arctic. Hourly CH_4_ fluxes were recorded between June–August 2019 and 2021 from three common vegetation types: dwarf-shrub tundra with lichen cover lacking vascular plants (hereafter, lichen), deciduous and evergreen dwarf-shrub cover (hereafter, shrub) and tussock (hereafter, tussock) coverage (Supplementary Fig. 1). To judge the spatial representativeness of these quasicontinuous, site-specific measurements, we conducted manual chamber measurements at three additional sites across permafrost zones in the Arctic (Supplementary Tables 1–3). We find that Arctic soil CH_4_ uptake is substantial and controlled by a complex suite of abiotic and biotic drivers. Strong CH_4_ uptake coincided with dry periods but we discovered diel and seasonal variability that could not be explained by temperature and moisture variability. Instead, this variability was related to biotic processes, as indicated by a close link with ecosystem CO_2_ respiration (ER), consistent with stimulation of the CH_4_ sink through addition of labile C.

## Seasonal variation of methane uptake in Arctic tundra

**Fig. 1:**
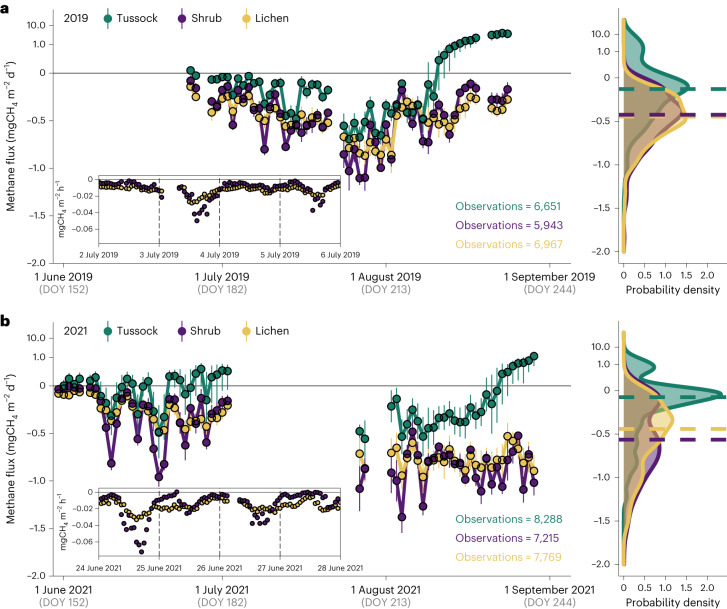
Methane fluxes at Trail Valley Creek. **a**,**b**, Seasonal CH_4_ flux dynamics measured with automated chambers at Trail Valley Creek during 2019 (**a**) and 2021 (**b**). Fluxes were measured between DOY 172–236 in 2019 and DOY 150–243 in 2021. Also shown is the probability density distribution of the data. Flux data show daily sums of hourly measured fluxes and are microsite means ± s.e. of transparent and opaque chambers combined (*n* = 6 for lichen and tussock, *n* = 5 for shrub). Insets show diel variation in CH_4_ uptake, measured hourly, from lichen and shrub for two selected 4 day periods (2–6 July 2019 and 24–28 June 2021; date labels start at 00:00 of the labelled day). Negative flux values denote net CH_4_ uptake. Dashed lines in the probability density plots indicate the median flux. Note that positive CH_4_ fluxes (emissions) are shown on a log scale.

Methane uptake at Trail Valley Creek occurred consistently (95% of fluxes) throughout the measurement periods (Fig. 1). Rates were −0.020 ± 0.016 mgCH_4_ m^−2^ h^−1^ (mean ± s.d.) from lichen sites and −0.024 ± 0.027 mgCH_4_ m^−2^ h^−1^ from shrub sites (Supplementary Table 4), corresponding to a daily flux of −0.49 ± 0.33 and −0.59 ± 0.51 mgCH_4_ m^−2^ d^−1^, respectively. Uptake rates were considerably larger than in recently synthesized data where dry tundra is reported as a growing season CH_4_ source^4^. Even the typically wetter tussock sites displayed CH_4_ uptake in 67% of fluxes, although average growing season fluxes were net zero (mean, 0.003 mgCH_4_ m^−2^ h^−1^; median, −0.003 mgCH_4_ m^−2^ h^−1^; Supplementary Table 4) due to emissions during rainy periods in early and late summer (Fig. 1).

**Fig. 2:**
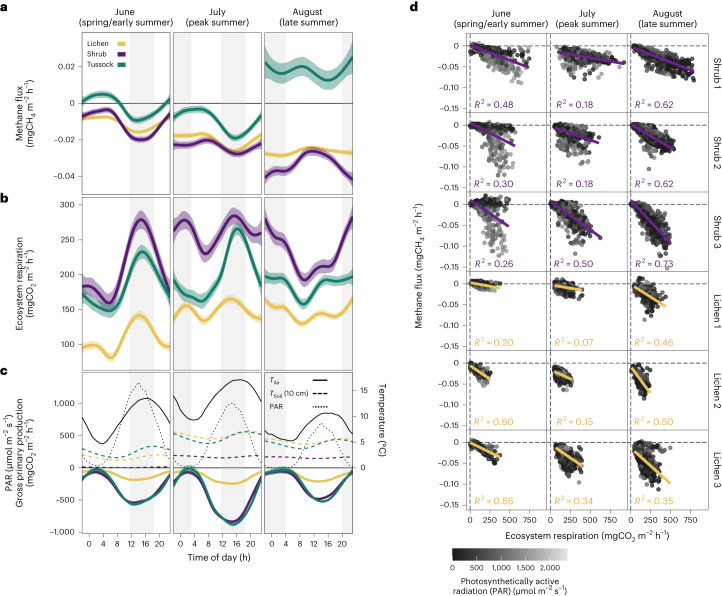
Diel variation in methane fluxes and relationship between methane uptake and ecosystem respiration at Trail Valley Creek. **a**–**c**, Diel variation in CH_4_ fluxes (**a**), ER (**b**) and gross primary production (GPP), temperature and PAR (**c**); measured with automated chambers at Trail Valley Creek, split by early (June), peak (July) and late summer (August). Note that despite 24 h day light in June and July, PAR is <100 µmol m^−2^ s^−1^ between 23:00 and 06:00, resembling night-time conditions. Fluxes are shown as smoothed means with 99% confidence intervals (generalized additive model smoothing) of each class based on hourly values measured during 2019 and 2021. Negative values denote net carbon uptake. Grey shaded areas indicate periods with peaks in CH_4_ consumption and ER. Graphs are based on transparent and opaque chambers combined (*n* = 6 per vegetation type). ER was measured directly with opaque chambers. For transparent chambers, ER was calculated as the mean of each vegetation type measured with opaque chambers (*n* = 3) and GPP was calculated as net ecosystem exchange (measured with transparent chambers) minus ER. **d**, Relationship between CH_4_ flux and ER and *R*^2^ of regression line, for the three individual opaque chambers of shrub and lichen.

**Extended Data Fig. 1:**
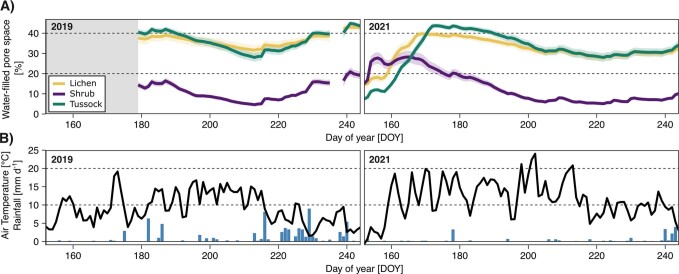
Meteorological conditions at Trail Valley Creek during the study years. Seasonal dynamics of soil water-filled pore space (**a**), air temperature and rainfall (**b**) measured at Trail Valley Creek during 2019 and 2021. Soil water-filled pore space was measured next to each opaque flux collar and is shown as mean with 95% confidence interval, n = 3, 0–12 cm depth). Grey shaded area shows missing data before sensor installation). Air temperature (black line) and daily precipitation (blue bars) were measured at the nearby weather station (n = 1).

Methane uptake was largest during mid to late summer for lichen and shrub (Fig. 1) coinciding with low water-filled pore space (WFPS; lichen, <35%; shrub, <15%; Extended Data Fig. 1). Late summer CH_4_ uptake was larger during 2021, which was warmer and drier than 2019 and the long-term climate normal (Fig. 1, Extended Data Fig. 1 and Supplementary Table 5). The association between drier soils and larger CH_4_ uptake has been reported in polar deserts and dwarf-shrub tundra, as well as the forest-tundra ecotone^12–14^. Average fluxes during August 2021 were higher from shrub (−0.044 ± 0.034 mgCH_4_ m^−2^ h^−1^) than from lichen (−0.032 ± 0.016 mgCH_4_ m^−2^ h^−1^) sites, despite cooler soils under shrubs (Supplementary Figs. 2 and 3). Methane uptake for shrub sites displayed a larger seasonal variability and diel magnitude than for lichen (Figs. 1 and 2a and Supplementary Fig. 4), indicating that the presence of vascular plants as well as plant development stage may influence CH_4_ uptake.

## Diel pattern of tundra methane uptake

**Extended Data Fig. 2:**
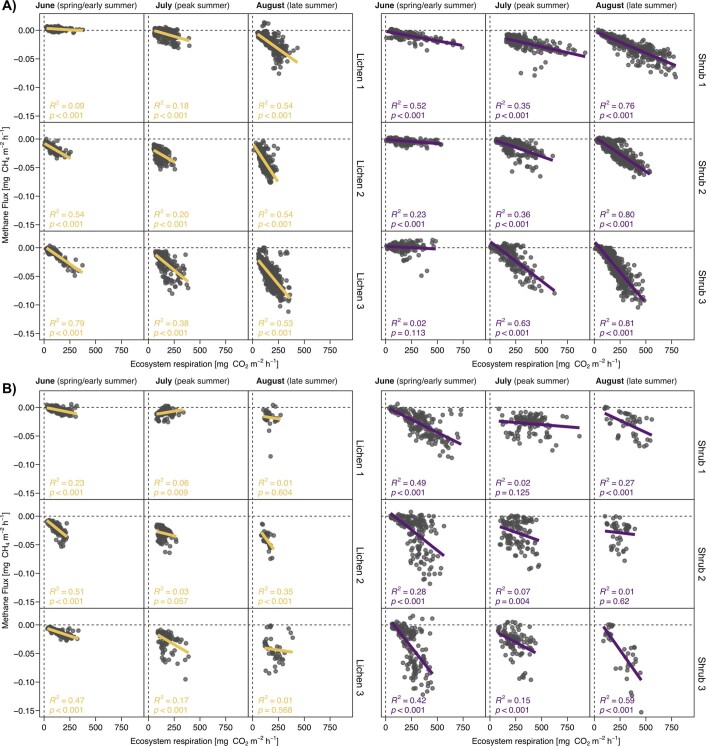
Relationship between methane uptake and ecosystem respiration at Trail Valley Creek. Correlation plots between methane (CH_4_) flux and ecosystem respiration for the three individual opaque chambers on shrub and lichen. **a**) Fluxes measured during low-light periods at photosynthetically active radiation (PAR) ≤ 120 µmol m^−2^ s^−1^. **b**) Fluxes measured during high light periods with PAR >1000 µmol m^−2^ s^−1^. Negative values denote CH_4_ uptake.

Besides seasonal variation, CH_4_ uptake showed distinct diel dynamics and a seasonal shift in the timing of CH_4_ uptake peaks (Fig. 2a). During June, the largest CH_4_ uptake occurred in the afternoon (15:00–16:00), broadly corresponding to daily maxima in air temperature, PAR and ER (Fig. 2a–c). Afternoon peak CH_4_ uptake was two to five times higher than during nocturnal (04:00–07:00) minima (Supplementary Table 6) and maximum CH_4_ uptake occurred exclusively at PAR > 1,000 µmol m^−2^ s^−1^ (Fig. 2c and Extended Data Fig. 2). During July, the PAR and temperature dependency was weaker and differences between night time and daytime fluxes were less pronounced. Unexpectedly, the diel pattern of CH_4_ uptake reversed during August, peaking between 22:00 and 04:00 with rates 21–50% larger than during the daytime minimum (Fig. 2a and Supplementary Table 6).

The presence and timing of diel peaks in CH_4_ uptake have repercussions for estimating Arctic C budgets, considering that manual measurements of plot-scale tundra CH_4_ fluxes are often made on a weekly to biweekly basis, using one measurement during daytime to obtain seasonal CH_4_ budgets via interpolation^4,14,18,25^. Our automated chamber measurements show that diel rates of CH_4_ uptake are not uniform and are more variable than diel patterns of soil temperature. Given the seasonal shift in the diel pattern, limiting measurements to daytime only overestimates daily CH_4_ uptake by 25–37% during early summer but may underestimate uptake by 6–19% during late summer (Supplementary Table 7).

We observed a surprisingly strong correlation between CH_4_ uptake and ER, particularly for shrub (*R*^2^ up to 0.73; Fig. 2d and Extended Data Fig. 2). The correlation with ER was highest in late summer during low-light periods (lichen, *R*^2^ = 0.53–0.54; shrub, *R*^2^ = 0.76–0.81; Extended Data Fig. 2a). Counterintuitively, the strongest CH_4_ uptake (lichen, −0.028 mgCH_4_ m^−2^ h^−1^; shrub, −0.038 mgCH_4_ m^−2^ h^−1^) did not coincide with the highest air and surface soil temperature but occurred in late summer during night time at low air (<8 °C) and soil temperatures (<4 °C at 10 cm), matching ER peaks (Fig. 2a–c). Divergent responses of the ER flux components to temperature^30^ and supply of labile C (ref. ^31^) can cause respiratory processes to deviate from the traditionally assumed, strict positive temperature dependency^32^. A lower-than-expected temperature dependency of C cycle processes may result in severe biases for seasonal and annual CO_2_ and CH_4_ flux budgets^30,33,34^.

The close link between CH_4_ uptake and CO_2_ respiration observed here and noted earlier in temperate forests^35,36^ indicates that input of labile C, such as methanol or formaldehyde^24,37^, to the rhizosphere may be an important mechanism promoting methanotrophic activity in tundra. This ‘rhizodeposition’—a process during which plants allocate assimilated C to soil via living roots^38^—promotes soil organic matter decomposition^31,39,40^ and nutrient mobilization and availability^38,41^. While high-affinity methanotrophs use CH_4_ as a C and energy source in aerobic respiration^24^, most methanotrophs require additional C compounds, such as CO_2_ and carbon monoxide (CO), or nitrogen (N) for growth^42,43^ and the supply of these elements may stimulate methanotrophic activity^44,45^. The seasonal evolution of diel dynamics, that was particularly pronounced for shrub (Fig. 2a), links CH_4_ uptake to ER and suggests that plant and rhizosphere processes may mediate the microbial consumption of CH_4_ in tundra soils. Regardless of the biogeochemical mechanism, the correlation between CH_4_ uptake and ER opens new opportunities to accurately model CH_4_ uptake based on ER measurements in combination with other abiotic (for example, soil moisture and temperature) and biotic variables (for example, biomass and microbial community)—most of which are easier and more cost-efficient to measure than CH_4_ fluxes.

## Drivers of the temporal variation of methane uptake

**Fig. 3:**
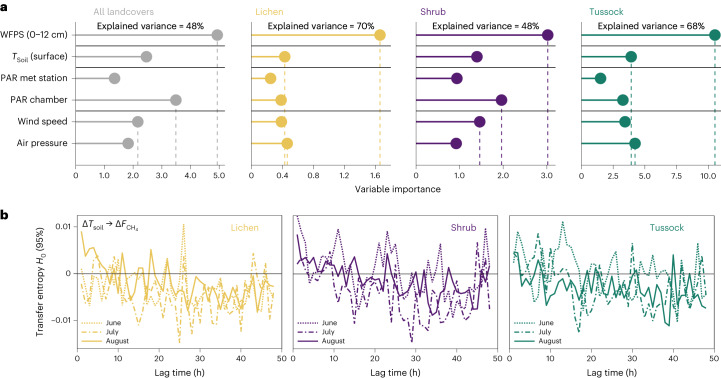
Relative importance of abiotic variables on methane fluxes at Trail Valley Creek. **a**, Relative importance of abiotic variables on hourly measured CH_4_ fluxes determined with an RF model for lichen, shrub and tussock measured with automated chambers at Trail Valley Creek. For relative importance of variables for data aggregated to different timescales see Extended Data Fig. 3 and for the direction of the responses see Extended Data Fig. 4. The RF analysis used data from years 2019 and 2021 (36,782 observations; Supplementary Table 8). The RF model for all vegetation types (*n* = 18 chambers) includes only CH_4_ uptake, whereas for the individual vegetation types (*n* = 6 chambers), all data were included. The three most important variables are indicated by vertical dashed lines. Note that ‘PAR chamber’ denotes measurements in opaque and transparent chambers (PAR set to 0 µmol m^−2^ s^−1^ in opaque chambers), whereas ‘PAR met station’ was measured at the nearby weather station. *T*_Soil_ (surface) denotes surface soil temperature, measured at the soil surface below the vegetation or lichen layer. **b**, Lagged effects of surface soil temperature on CH_4_ fluxes estimated from transfer entropy (positive values indicate significant effects with a 95% confidence interval).

**Extended Data Fig. 3:**
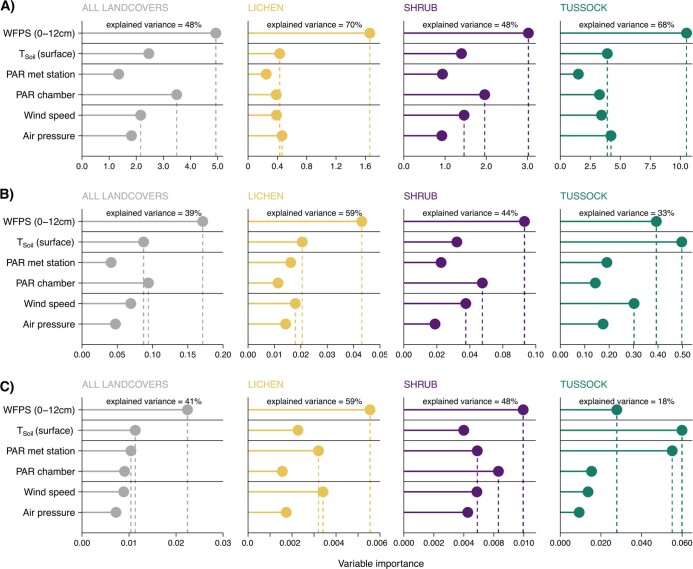
Relative importance of abiotic variables on methane fluxes at trail Valley Creek at different temporal scales. Relative importance of abiotic variables on **a**) hourly measured, **b**) aggregated daily mean and **c**) aggregated weekly mean methane fluxes determined with a Random Forest (RF) model for lichen, shrub, and tussock sites measured with automated chambers at Trail Valley Creek. RF was based on 36782 measurements (hourly, **a**), 1930 measurements (daily aggregates, **b**) and 321 measurements (weekly aggregates, **c**). The RF model for all vegetation types (18 chambers) includes only CH_4_ uptake, whereas for the individual vegetation types (6 chambers per vegetation type), all fluxes were included. This includes mainly CH_4_ uptake for lichen and shrub, but occasional emissions from tussock. Variables are grouped from top to bottom into ‘moisture-related’, ‘temperature-related’, ‘PAR-related’, and ‘other meteorological variables’, and the three most important variables are indicated by vertical dashed lines. Note that ‘PAR chamber’ considers opaque and transparent chambers (PAR = 0 µmol m^−2^ s^−1^ in opaque chambers), whereas PAR met station are actual site PAR data. T_Soil_ = soil temperature, WFPS = soil water-filled pore space.

**Extended Data Fig. 4:**
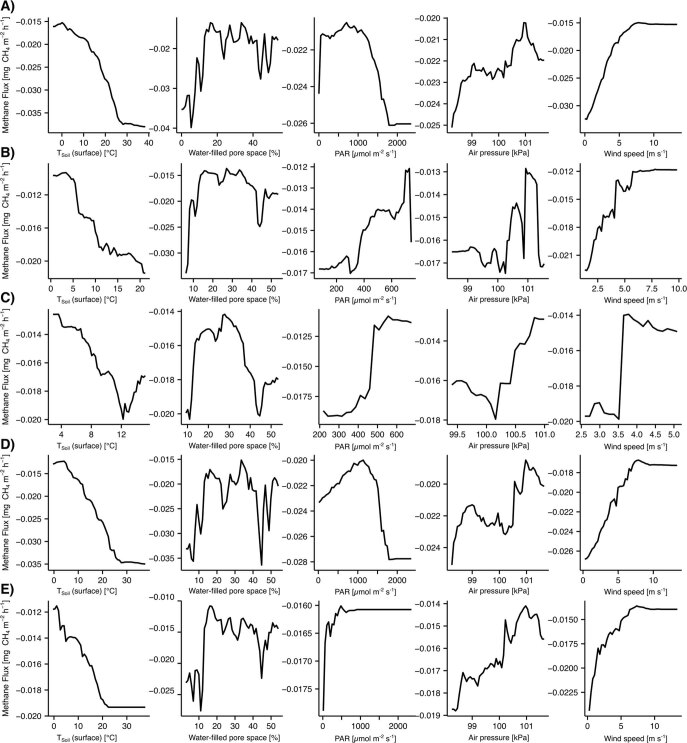
Partial dependence plots of abiotic variables in Random Forest model. Partial dependence plots as outcome of the Random Forest (RF) models in Fig. 3a and Extended Data Fig. 3. for all vegetation types (18 chambers). Plots show the dependence of methane fluxes on surface soil temperature (T_Soil_ (surface)), water-filled pore space, photosynthetically active radiation (PAR, measured as photon flux density), air pressure and wind speed. Partial dependence is plotted for models using hourly measured fluxes (**a**) as well as data aggregated to daily (**b**) and weekly timescales (**c**), as well as fluxes measured during daytime (**d**) and night time (**e**).

**Extended Data Fig. 5:**
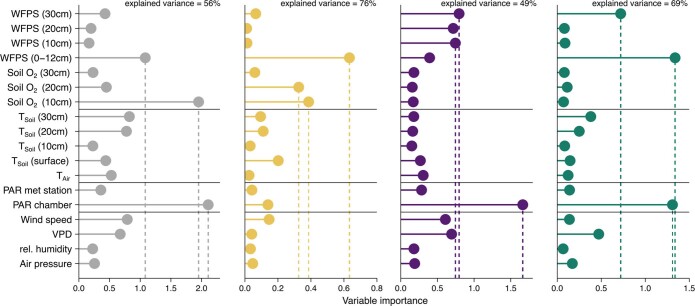
Relative importance of a wide range of abiotic variables on methane fluxes at Trail Valley Creek. Relative importance of abiotic variables on hourly measured methane fluxes determined with a Random Forest (RF) model for lichen, shrub, and tussock sites measured with automated chambers at Trail Valley Creek. The RF analysis was performed with data collected during year 2021 (20 531 observations) with a larger set of measured abiotic variables compared to Fig. 3 (see Supplementary Table 7 for details). The RF model for all vegetation types (18 chambers) includes only CH_4_ uptake, whereas for the individual vegetation types (6 chambers per vegetation type), all fluxes were included, meaning mainly CH_4_ uptake for lichen and shrub, but occasional emissions from tussock. Variables are grouped from top to bottom into ‘moisture-related’, ‘temperature-related’, ‘PAR-related’, and ‘other meteorological variables’, and the three most important variables are indicated by vertical dashed lines. Note that ‘PAR chamber’ denotes measurements in opaque and transparent chambers (PAR set to 0 µmol m^−2^ s^−1^ in opaque chambers), whereas ‘PAR met station’ the nearby weather station are actual site PAR data. WFPS = soil water-filled pore space, VPD = vapour pressure deficit, O_2_ = oxygen, T_Air_ = air temperature, T_Soil_ (surface) = surface soil temperature, measured at the soil surface below the vegetation or lichen layer.

To rate the relative importance of abiotic variables on CH_4_ uptake at Trail Valley Creek, we applied a random forest (RF) model to our automated chamber dataset (36,782 observations; Supplementary Table 8). The RF analysis showed that WFPS was the most important abiotic control on CH_4_ uptake (Fig. 3a and Supplementary Fig. 5). The importance of temperature was smaller but gained importance when data were aggregated to longer timescales (hourly versus daily and weekly; Extended Data Figs. 3 and 4). Lagged effects of surface soil temperature on CH_4_ uptake were weak and typically lasted <4 h, except for June when significant lags occurred over longer timescales for shrub and tussock (Fig. 3b). Separate RF models for the 2021 measurement season with a larger range of measured predictors confirmed WFPS and oxygen availability as the most important predictors, particularly for lichen (Extended Data Fig. 5). For tussock, the only vegetation type displaying CH_4_ emissions, WFPS at 30 cm depth was an important predictor. Probably, CH_4_ production in tussock occurs in deeper, wetter layers caused by shallower thaw (Supplementary Fig. 6) and air-filled sedge tissue facilitates gas transport to the atmosphere^23^. Fluxes from shrub and tussock, where vascular plants are present, further showed dependence on PAR (Fig. 3a), indicating a link between CH_4_ uptake and plant functioning through processes such as enhanced evapotranspiration affecting soil WFPS or input of labile C.

**Fig. 4:**
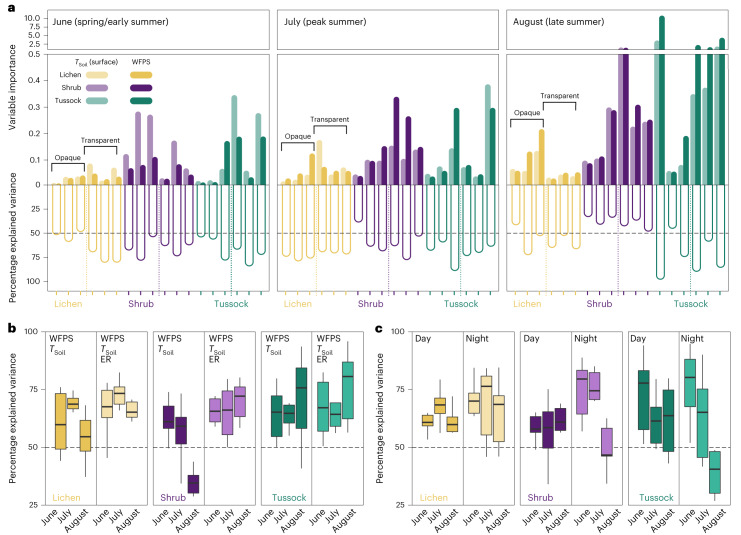
Relative importance of soil moisture, temperature and ecosystem respiration on methane fluxes at Trail Valley Creek. **a**, Relative importance of the two abiotic predictors, surface soil temperature (*T*_Soil_ (surface)) and soil WFPS on methane (CH_4_) fluxes and percentage of variance explained by these two variables. Variable importance and explained variance were determined with an RF approach modelling CH_4_ fluxes measured with automated chambers on lichen, shrub and tussock at Trail Valley Creek. Data were split by chamber (18 chambers) and month. Figure shows individual chambers, of which the first three chambers of each vegetation type are opaque and the other three are transparent. **b**, Explained variance of **a** compared to an RF model with inclusion of ER as predictor. **c**, Model output of RF model with inclusion of ER for fluxes measured during daytime and night time. Boxplots are derived from *n* = 6 chambers per vegetation type and show median (thick black line), upper and lower quartile (boxes) and the highest and lowest values (black vertical lines).

**Extended Data Fig. 6:**
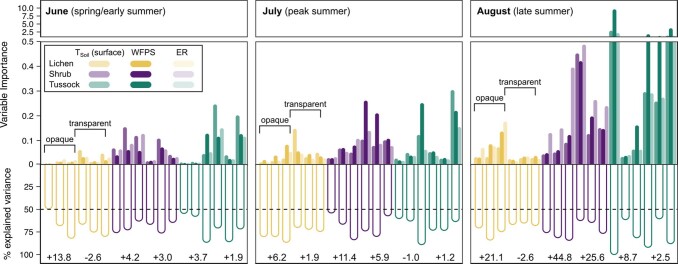
Relative importance of soil moisture and temperature on methane fluxes at Trail Valley Creek as well as the additional explanatory power of ecosystem respiration. Relative importance of the two important predictors, surface soil temperature and soil water-filled pore space (WFPS), of methane (CH_4_) fluxes measured with automated chambers at Trail Valley Creek for lichen, shrub, and tussock sites, as well as the additional percentage of variance explained by ecosystem respiration (ER). Data were split by chamber (18 chambers) and month. The additional percentages of variance explained by ER is indicated as the % change of model fit compared to fitting the model to only soil temperature and WFPS. See Supplementary Fig. 7 for the same RF model using soil temperature at the 20-cm depth instead of surface soil temperature.

**Extended Data Fig. 7:**
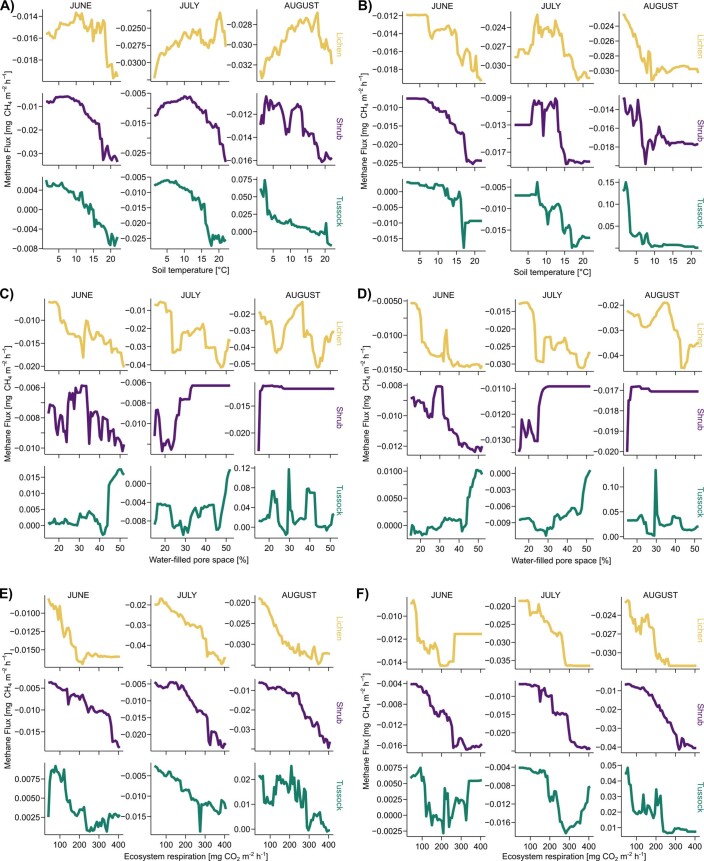
Partial dependence plots of soil temperature, moisture, and ecosystem respiration in Random Forest model. Partial dependence plots as outcome of the Random Forest (RF) models. Plots show the dependence of methane fluxes on surface soil temperature (**a** and **b**), soil water-filled pore space (**c** and **d**) as well as on ecosystem respiration (**e** and **f**). Partial dependence was plotted for RF models using hourly measured fluxes (**a, c, e**) as well as the daily aggregated mean flux (**b, d, f**).

The RF models explained 48–76% of the flux variance for individual vegetation types, with the highest percentage explained for lichen sites (70–76%), whereas for shrub 51% of the flux variance remained unexplained (Fig. 3a, Extended Data Fig. 5 and Supplementary Table 8). Soil temperature was important in explaining CH_4_ fluxes during early summer only, whereas WFPS was the most important predictor during other periods (Fig. 4a). Surface soil temperature and WFPS alone explained over 50% of the flux variance for most chambers, with a drop in explanatory power of these two variables during late summer, particularly for shrub sites (Fig. 4a). The seasonal decline in explanatory power and the large portion of unexplained variance for shrub suggests that CH_4_ uptake may be further governed by biotic processes in the plant–soil continuum. Given the observed link between CH_4_ uptake and ER, we added ER as a predictor in the RF analysis. Adding ER—a function of microbial activity and substrate supply—improved RF model performance substantially during late summer, where ER explained an additional 26–45% of variance in CH_4_ uptake for shrub sites (Fig. 4b and Extended Data Figs. 6 and 7); however, predictive performance was notably poor for late summer night-time fluxes (Fig. 4c).

## Variability of the upland soil methane sink across the Arctic

**Fig. 5:**
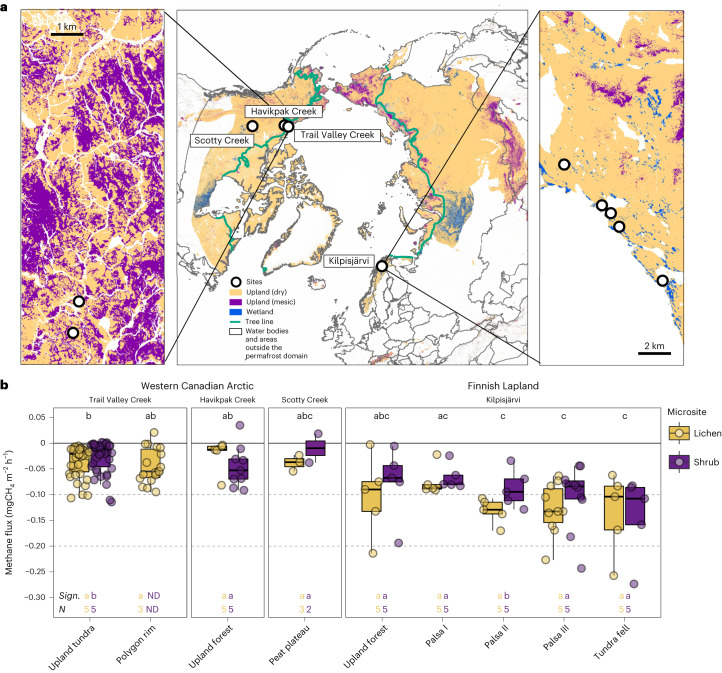
Methane fluxes at the study sites and distribution of uplands and wetlands across the Arctic. **a**, Map of study sites in the western Canadian and European (Sub-)Arctic and regional maps of the intensively sampled sites, Trail Valley Creek and Kilpisjärvi. The upland class is split into dry uplands (high potential for methane (CH_4_) uptake) and wetter, graminoid-dominated, mesic uplands (fluctuation between CH_4_ sink and source). **b**, Spatial variability of CH_4_ fluxes measured with manual chambers at typical, well-drained tundra and boreal land cover types. Boxplots show median (thick, black line), upper and lower quartile (boxes), the highest and lowest values (black vertical lines) and all measured values (circles). Numbers indicate count of replicate collars (*N*). Negative values denote CH_4_ uptake. Fluxes were measured in mid to late summer at all sites, except at Havikpak Creek, where fluxes were measured in early summer (Supplementary Table 2). Statistically significant differences (two-tailed) are indicated by letters, where different letters show differences at *P* ≤ 0.05 (land cover type, Dunn’s test, adjusted *P* values; vegetation type, Welch’s two-sample *t*-test). Exact *P* values for the differences between lichen and shrub are, from left to right, 0.031, ND, 0.391, 0.564, 0.251, 0.047, 0.257, 0.754. ND, not determined. Credit: **a**, ESRI, USGS.

To gain a wider geographical perspective on CH_4_ uptake in Arctic uplands, we conducted manual chamber measurements at three additional sites in the western Canadian and European Arctic and Sub-Arctic (Fig. 5a). We selected representative lichen and shrub plots at well-drained upland land cover types (WFPS <50%; Supplementary Tables 1–3) within the tundra and boreal biomes, as well as small-scale landforms with thick organic soils. On the basis of 176 campaign-based, growing season flux observations, all sites acted as CH_4_ sinks (Fig. 5b and Supplementary Tables 9 and 10). We observed significantly (*P* < 0.001) higher CH_4_ uptake in Finnish Lapland (mean, −0.143 mgCH_4_ m^−2^ h^−1^; median, −0.120 mgCH_4_ m^−2^ h^−1^; Supplementary Table 10) compared to the Canadian Arctic (mean, −0.041 mgCH_4_ m^−2^ h^−1^; median, −0.039 mgCH_4_ m^−2^ h^−1^). Measured soil gas concentrations revealed below ambient CH_4_ concentrations down to 20 cm depth at most sites, indicating active CH_4_ consumption in the soil profile and corroborating our observation of higher CH_4_ uptake at the Finnish sites (Supplementary Fig. 8).

Extrapolating manual measurements to daily flux units, CH_4_ uptake based on median and mean values (Supplementary Table 10) was −2.88 to −3.43 mgCH_4_ m^−2^ d^−1^ in Finnish Lapland and −0.94 to −0.98 mgCH_4_ m^−2^ d^−1^ across all Canadian sites. At Trail Valley Creek, median fluxes (−0.67 mgCH_4_ m^−2^ d^−1^) matched automated chamber observations (July, −0.55 mgCH_4_ m^−2^ d^−1^; August, −0.72 mgCH_4_ m^−2^ d^−1^), whereas manual chamber means (−1.06 mgCH_4_ m^−2^ d^−1^) overestimated CH_4_ uptake compared to automated chambers. Observed CH_4_ uptake was larger than recent estimates for dry tundra, which is estimated as a CH_4_ source based on observations from 63 sites (mean, +3.83 mgCH_4_ m^−2^ d^−1^; median, −0.01 mgCH_4_ m^−2^ d^−1^) (ref. ^4^) and conventional, process-based CH_4_ models parameterized for wetlands (+0.57 mgCH_4_ m^−2^ d^−1^) (ref. ^46^). Uptake was also higher than reported for the boreal biome (mean, −1.1 mgCH_4_ m^−2^ d^−1^; median, −0.4 mgCH_4_ m^−2^ d^−1^) (ref. ^4^). Compared to studies using portable laser instruments, uptake rates at Trail Valley Creek were of the same magnitude as measured in the Canadian High Arctic^17^ and Western Greenland^12^. Even higher uptake rates (<−3 mg m^−2^ d^−1^) are not uncommon in the Arctic^15,16,25^.

## Soil biogeochemical controls on Arctic soil methane uptake

**Extended Data Fig. 8:**
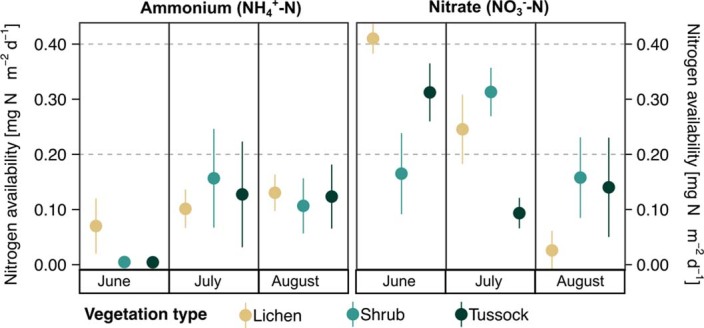
Inorganic nitrogen availability at Trail Valley Creek. Plant-available nitrogen (N; mean ± standard deviation, n = 5) for lichen, shrub, and tussock sites at Trail Valley Creek during 2019. The presence of nitrate (NO_3_^−^) and negative correlation between ammonium (NH_4_^+^) and NO_3_^−^ indicates nitrification is an active process at Trail Valley Creek. During nitrification, NH_4_ is oxidized to NO_3_^−^ via several intermediates. The low amounts of NH_4_ in early summer (June) under shrub and tussock indicate NH_4_^+^-limitation, due to high microbial N demand (for example, through nitrification) and plant N demand during onset of the plant growth phase, but a lifting of N-limitation in late summer when plant N demand is lower during the senescence period.

It is known that methanotrophs are metabolically capable of utilizing a variety of substrates including hydrogen, ammonia, dinitrogen, CO and sulfur compounds^42,43,45,47^ and methanotrophic activity is also linked to the availability of phosphorus^48^, copper^24^ and other elements. Importantly, methanotrophs require bioavailable N to sustain their metabolism and N is the main nutrient limiting plant and microbial growth in Arctic ecosystems^49,50^. One chief competitor for N resources, in particular ammonium (NH_4_^+^), are nitrifiers^24,44,51^. Nitrification, the conversion of NH_4_^+^ to nitrate (NO_3_^−^), is an active process at Trail Valley Creek (Extended Data Fig. 8), manifesting direct competition between methanotrophs and nitrifiers for N substrates.

**Fig. 6:**
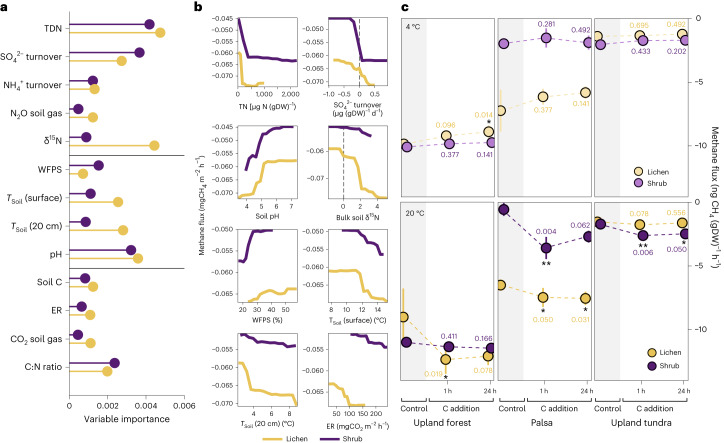
Relationship between methane fluxes, abiotic and biotic variables in uplands of Trail Valley Creek and Kilpisjärvi. **a**, Relative importance of variables on methane fluxes measured at Trail Valley Creek and Kilpisjärvi determined with an RF model. Variable importance is grouped from top to bottom into variables related to nutrient cycling, abiotic variables and variables related to C cycling. **b**,**c**, Partial dependence plots of the RF output for selected variables (**b**) and oxidation rates (mean ± s.d.; *n* = 4) measured during soil incubations before and after C addition from soils collected at Kilpisjärvi (**c**). Differences between control and C-addition treatments (two-tailed) were determined with Dunn’s test and unadjusted *P* values are significant at *P ≤ 0.05 and **P ≤ 0.01. Exact *P* values are indicated in the figure. Carbon was added as glucose and measurements took place before C addition (control), within 1 h after C addition, as well as 24 h after C addition. DW, dry weight.

**Extended Data Fig. 9:**
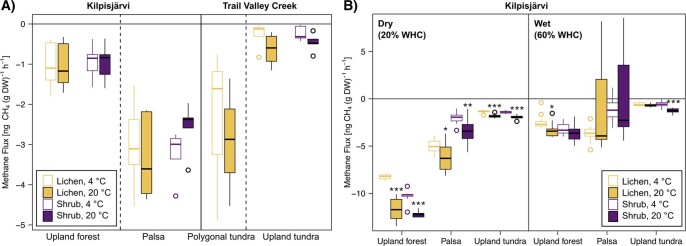
Methane fluxes during laboratory incubations. Methane (CH_4_) fluxes measured during incubations at 4 °C and 20 °C from soil samples (0–10 cm) collected at Trail Valley Creek and Kilpisjärvi (**a**), as well as CH_4_ fluxes measured under dry conditions (water-holding capacity (WHC) = 20 %) and wet conditions (WHC = 60 %) from soils collected at Kilpisjärvi (**b**). Soil sampling in **a**) took place in year 2021 corresponding to the observed *in situ* CH_4_ flux rates, whereas soil sampling in **b**) took place in 2022 from plots located within ~50 m of the original locations sampled during 2021. Differences between 4 °C and 20 °C in **a**), determined by Welch’s two-sample t-test (two-tailed), were not statistically significant. Differences (two-tailed) in **b**) were statistically different at p ≤ 0.05 (*), p ≤ 0.01 (**) and p ≤ 0.001 (***). DW = dry weight. The incubation in **a**) used a replication (biological replicates) of n = 5 for Upland forest, Palsa and Upland tundra (Shrub), n = 4 for Upland tundra (Lichen), and n = 3 for Polygonal tundra, and in **b**) a replication (technical replicates) of n = 4. Boxplots show median (thick, horizontal lines), upper and lower quartile (boxes), highest and lowest values (thin, vertical lines), and outliers (circles). Exact p-values in **b**) from left to right: 3.8×10^−6^, 3.4×10^−7^, 0.03, 0.004, 7.6×10^−6^, 6.1×10^−8^, 0.01, 0.18, 0.09, 0.43, 0.10, 9.3×10^−6^.

Across sites, soil pH and variables related to nutrient availability had the highest relative importance in RF analysis (Fig. 6a). Partial dependence plots (Fig. 6b) show that CH_4_ uptake increased with higher dissolved N concentration, sulfate (SO_4_^2−^) turnover rate, soil δ^15^N, ER and soil temperature and decreased with higher pH and WFPS. Soil pH is important in regulating microbial community composition, with functional differences between the methanotrophic upland soil cluster alpha (acidic soils; Finnish Lapland) and gamma communities (neutral and alkaline soils; Trail Valley Creek; Supplementary Table 9)^43,52^. Matching in situ observations, differences between CH_4_ uptake determined at 4 and 20 °C during laboratory incubations were only statistically significant under dry conditions (Extended Data Fig. 9) but labile C addition significantly enhanced CH_4_ uptake under the 20 °C treatment (Fig. 6c). Together with the link to ER fluxes observed at Trail Valley Creek, our findings imply that biotic controls are important in driving the Arctic CH_4_ sink, whereas temperature becomes an important secondary control under favourable substrate and moisture conditions.

## Discussion and conclusions

Consumption of trace gases is an important process in substrate-limited environments^47^, where methanotrophs derive their energy from atmospheric CH_4_ and create a growing season CH_4_ sink in Arctic uplands. Our results suggest that soil moisture is the most important abiotic driver of CH_4_ uptake, with drier soils leading to increased CH_4_ uptake. Temperature, known to stimulate CH_4_ emissions^8^, showed seasonally variable and complex effects on CH_4_ uptake that varied by vegetation type. Other important controls on CH_4_ uptake frequently override the effect of temperature in Arctic and other biomes^2,26,53^. Our findings highlight that observed drastic high-latitude warming^54^ itself will promote atmospheric CH_4_ uptake less than predicted large-scale drying^55,56^.

We find that abiotic controls alone cannot explain seasonal and diel patterns of CH_4_ uptake, particularly when shrubs are present—an important caveat considering Arctic shrubification^57^. As recently noted for CH_4_ emissions^33^, biotic drivers related to plant and microbial functioning added substantial explanatory power for observed CH_4_ uptake rates. We show that CH_4_ uptake reflects diel patterns in ER. Although the underlying mechanisms remain unclear, our findings indicate that methanotrophic activity in Arctic uplands thrives with additional input of bioavailable C and N sources. We propose that, in Arctic soils, where plants and microbes compete intensively for C and nutrients^40,50^, rhizosphere processes are important and that alleviated nutrient limitation through rhizodeposition^39^ may create a negative C feedback by stimulating soil CH_4_ uptake. Competition for N substrates between methanotrophs and nitrifiers, as well as rhizodeposition of C and N are two important links between the two major biogeochemical cycles^51^. Both are regulatory mechanisms mediating CH_4_ removal from the atmosphere.

Importantly, our study highlights that the Arctic CH_4_ sink may currently be underestimated, corroborating recent reports that the inclusion of high-affinity CH_4_ oxidation in process-based models may more than double the Arctic CH_4_ sink to 6.2–9.5 TgCH_4_ yr^−1^ (ref. ^7^). A first-order approximation places our results in the same range (Supplementary Table 11), adding evidence that CH_4_ uptake may drive the discrepancy in Arctic CH_4_ budgets derived by bottom-up and top-down inversion estimates^1,6^. However, for a refined estimation of the Arctic CH_4_ budget, our study emphasizes the need to (1) record night time and non-growing season CH_4_ uptake; (2) apply new relationships identified in this study to upscale CH_4_ uptake; (3) identify microbiological mechanisms and plant–soil interactions regulating Arctic CH_4_ uptake; (4) produce high-resolution data products of Arctic wetland versus upland extent; and (5) measure and report CH_4_ fluxes from low-emitting sites that act as ‘cold spots’ in the Arctic to correct the observation-bias towards high-emitting wetlands. Considering the immense gaseous and lateral losses of C associated with thawing permafrost and their climatic impact^58^, we need to understand natural sinks, their capacity to balance emissions and their response to a changing Arctic.

## Methods

### Site description

Automated chamber and auxiliary measurements were carried out at Trail Valley Creek (68° 44′ 32″ N, 133° 29′ 55″ W, 68 m above sea level (a.s.l.), mean annual air temperature (MAAT) −8.2°C, mean annual precipitation (MAP) 241 mm), an upland tundra site located 45 km north of Inuvik, Northwest Territories, in the western Canadian Arctic. Additional manual chamber measurements were carried out at three sites in the western Canadian and European Arctic and Sub-Arctic (Fig. 5a and Supplementary Tables 1–3): Havikpak Creek, Northwest Territories (68° 19′ 15″ N, 133° 31′ 05″ W, 68 m a.s.l.), Scotty Creek, Northwest Territories (61° 18′ 29″ N, 121° 18′ 01″ W, 169 m a.s.l., MAAT −2.8 °C, MAP 388 mm) and Kilpisjärvi in Finnish Lapland (68° 51′ 54″ N, 21° 06′ 24″ E, 85 m a.s.l., MAAT −1.9 °C, MAP 487 mm). All sites are located in the northern circumpolar permafrost region and extend from the continuous permafrost zone in the north to the sporadic permafrost zone in the south. Sites span the tundra and boreal biomes and are described in detail in Supplementary Methods.

### Automated chamber flux measurements

A total of 18 automated chambers were installed within dwarf-shrub-dominated upland tundra at Trail Valley Creek (Supplementary Fig. 1), the most abundant land cover type in Arctic tundra^21^. Six replicates each were established on the three dominant vegetation types occurring within the selected area: lichen-, shrub- and tussock-dominated patches (referred to as lichen, shrub and tussock). Wooden boardwalks were installed to minimize disturbance when installing and accessing the automated chambers. Chamber installation took place in early June 2019 right after snowmelt coinciding with the onset of the growing season. Chambers were similar in design to the ones described by refs. ^59^ and ^60^ and consisted of transparent plexiglass domes (diameter, 51 cm; height, 20 cm; dome volume, 30 l) attached to PVC collars (diameter, 55 cm; height, 15 cm; wall thickness, 1 cm). The total effective chamber volume was 30–45 l. Motors (linear actuators, model FA-150-12-3″-P, Firgelli Automations) tightly closed the chambers and rubber seals at the bottom edge of the chamber lid (EPDM Foam rubber seals, 1″ wide, 1/6″ thick, McMaster-Carr) effectively sealed the chamber towards the atmosphere during chamber closure.

The chamber system started measuring gas concentrations and auxiliary variables on 21 June 2019. The domes of half of the chambers were covered with reflective thermal bubble wrap to block out photosynthetically active radiation (PAR) from *n* = 3 chambers per vegetation type (opaque chambers), while the other half of the chambers remained without an opaque cover (transparent chambers). Each chamber was equipped with a fan and a pressure equalization tube, ensuring headspace air mixing and preventing pressure differences during the measurement. Air temperature was monitored in each chamber using custom-made, calibrated type T copper-constantan thermocouples (Omega Sensing Solutions). We monitored PAR as photosynthetic photon flux density (PQS1-L, Kipp & Zonen) inside the nine transparent chambers and soil temperature and moisture (CS655-L Water Content Reflectometer Plus, Campbell Scientific) were measured next to each of the nine opaque chambers using vertically inserted, 12 cm long rods. These auxiliary data were logged at 10 s (PAR and air temperature) and 30 min intervals (soil moisture and soil temperature) and data were recorded with a CR1000X datalogger and AM16/32B multiplexer (Campbell Scientific).

Gas concentrations in the chamber headspace were measured with a Los Gatos Research (LGR) Enhanced Performance greenhouse gas analyser (Rackmount GGA-24EP 911-0010, Los Gatos), enhanced for thermal stability to provide ultrastable readings, with a precision (1 sigma at 1 s; Supplementary Table 12) of 1 ppb (CH_4_), 300 ppb (CO_2_) and 15 ppm (H_2_O), a maximum drift over 24 h of 5 ppb (CH_4_) and 300 ppb (CO_2_) and a measurement range of 0.01–100 ppm (CH_4_), 200–20,000 ppm (CO_2_) and up to 70,000 ppm for H_2_O. Given the specifications of our greenhouse gas analyser, the observed CH_4_ uptake rates in our study can clearly be distinguished from net zero fluxes. We used a measurement frequency of 1 Hz (enhanced performance, fast flow) and an external three-head diaphragm pump (N-920, 1.2 s flow-through time, 0.83 Hz, KNF Neuberger), bypassing the internal pump of the analyser. During operation, the flow rate was set to 2.75 l min^−1^ and gas temperature and pressure measured with the LGR were 51–52 °C and 139.4 Torr, respectively.

Each chamber was equipped with a 7 µm filter and water trap in the inlet tube to the LGR, to prevent water and particles entering the analyser. An additional 2 µm filter was installed directly at the gas analyser inlet. Inflow and outflow pressures from each chamber were monitored continuously and were typically 7–8 kPa. The chamber system further consisted of a flow meter (RMA 21-SSV, Dwyer Instruments), a pressure regulator and pressure sensor (MPX5100DP, Motorola), solenoid valves (EV-2M-12-H, Clippard) to switch the gas flow between chambers, a set of relays (Relay Controller SDM-CD16AC, Campbell Scientific) to control chamber lid movement, a CR1000X datalogger and SDM-CD16ACA relay controller (Campbell Scientific) and a PC (ARK-1124U-S1A1E, Advantech Corporation) merging flux data with auxiliary data and creating daily automated backups, as well as input files for flux processing. The external pump and LGR were placed in a temperature-controlled casing with a push–pull fan air circulation system. To provide a constant AC power source alleviating fluctuations in recorded gas concentrations caused by voltage spikes, the LGR was connected to an uninterruptible power supply unit (Tripp Lite SU1500RTXL2UA, Eaton). The chamber control system and gas analyser were placed on a wooden platform and sheltered by a McPherson tent (Fort McPherson Tent & Canvas). An inlet tube and outlet tube (each 38 m long; outside diameter, 6.35 mm; inner diameter, 4.3 mm; Synflex 1300 Metal-Plastic composite tubing, Eaton) connected each chamber with the control system.

The chamber system was powered by an onsite hybrid energy system providing AC power between 21 June and 24 August 2019 (day-of-year (DOY) 172–236) and 30 May and 31 August 2021 (DOY 150–243). Chamber closure times were 3 min per chamber. Data processing was performed with various routines developed inhouse in the MATLAB computing environment, v.R2020b (The MathWorks) and is described in Supplementary Methods. In total, 84% of CO_2_ fluxes were calculated using exponential fits and 16% using linear fits. As the chamber CH_4_ concentration increase or decrease was mostly small compared to that of CO_2_, fluxes of CH_4_ were preferentially calculated using linear fits. However, exponential fits were selected if they yielded a better fit for fluxes above a certain threshold (Supplementary Methods). A total of 93% of CH_4_ fluxes were calculated using linear fits and 7% using exponential fits. Data cleaning was applied for time periods with inconsistent inflow and outflow pressures, to make sure there was no underpressure or overpressure in the chambers. A final data cleaning step was carried out to account for periods with poor atmospheric mixing conditions, commonly occurring at night time at low wind speeds leading to an overestimation of fluxes measured with the chamber technique during those periods^59,61^. We used the friction velocity (${u}_{* }$) and wind speed (measured at 7 m height) to exclude fluxes measured between 23:00 and 07:00 that occurred when ${u}_{* }$ < 0.15 and wind speed <1.50 m s^−1^. After applying all data cleaning steps, 16% of CO_2_ fluxes and 15% of CH_4_ fluxes were discarded, resulting in a final dataset of 44,644 individual data points for CO_2_ and 44,848 measurement points for CH_4_ (sum of both measurement years). For obtaining diel fluxes of CH_4_, hourly measured fluxes were summed over each 24 h period for each individual chamber. If the number of observations was less than 24, short data gaps (<12 h) were filled using linear interpolation. Days with longer data gaps (>12 h) were treated as missing data.

### Manual chamber flux measurements

Manual chamber measurements at Trail Valley Creek were conducted one to two times per week between 15 June and 30 August 2019 (DOY 166–243) and measurements were made once at all other sites (Scotty Creek, September 2018 (DOY 255); Havikpak Creek, June 2021 (DOY 171); Kilpisjärvi, August 2021 (DOY 229–238); Supplementary Table 2). Manual chamber fluxes were measured during daytime (09:00–21:00, with most flux measurements between 11:00 and 15:00). For comparison of growing season fluxes between sites, measurements taken at Trail Valley Creek during the spring shoulder period (before DOY 171) were excluded from the analyses. Fluxes of CH_4_ were measured with portable greenhouse gas analysers, capable of measuring CH_4_, CO_2_ and H_2_O. At Scotty Creek we used an LGR gas analyser (LGR U-GGA-915 Ultraportable, Los Gatos) with a precision (1 sigma at 1 s) of <2 ppb for CH_4_, <300 ppb for CO_2_ and <100 ppm for H_2_O. At all other sites (Trail Valley Creek, Havikpak Creek and Kilpisjärvi), we used a Picarro gas analyser (G4301 GasScouter, Picarro) with a precision (1 sigma at 5 s) of 3 ppb for CH_4_, 400 ppb for CO_2_ and 100 ppm for H_2_O (Supplementary Table 12). Gas concentrations were recorded at 1 s intervals over an enclosure time of 5 min and fluxes were calculated on the basis of linear and nonlinear model fits using the chamberflux script^62^ in MATLAB v.R2020b. Further details on chamber design, flux calculation and quality control are provided in Supplementary Methods.

### Vegetation, soil gas and soil characteristics

To determine vegetation greenness, flux collar photographs were taken weekly to biweekly at Trail Valley Creek and once at all other sites. Collar greenness was calculated using the Canopeo beta version Foliage (v.1.0)^63^. We used a stainless-steel tube equipped with three-way valve to collect soil pore gas samples for determining concentrations of CH_4_, CO_2_ and N_2_O at 2, 5, 10 and 20 cm depths as well as in ambient air and samples for determining the stable isotopic signal of δ^13^CH_4_ and δ^13^CO_2_ in 10 cm depth (Supplementary Methods). Soil samples were collected at a subset of sites in the western Canadian and European Arctic (Supplementary Table 9) to link observed CH_4_ uptake rates with soil physical–chemical properties. Soil samples were collected from lichen and shrub plots at the manual chamber flux locations. At Trail Valley Creek, sampling was done on upland tundra and on polygonal tundra where soil samples were collected from the lichen-covered polygon rims (no shrub class present). At the Kilpisjärvi site, soil samples were collected from two locations, one of these a mountain birch forest on mineral soil (upland forest) and the other a permafrost peatland (Palsa II). Samples were taken from the top 0–10 cm. Soils were homogenized and visible roots removed within two days from sampling.

Soil pH was determined in a soil slurry with a 1:2 volume ratio of deionized water and fresh soil. Soil bulk density was determined by drying soil samples of known volume to a constant weight. WFPS was calculated on the basis of bulk density, volumetric water content and particle density^64^. For analysis of soil organic matter, soil dry weight and soil C and N content and the δ^13^C and δ^15^N isotopic signals, soil samples were oven dried to a constant weight at 65 °C for 48 h. Soil organic matter was determined via loss on ignition at 550 °C. Soil C and N were determined from homogenized soil after milling at 30 r.p.m. (Retsch MM301). Soil samples were measured for organic C and total N (TN) as well as for δ^13^C via dry combustion in an isotope cube element analyser (Elementar Analysensysteme) coupled to an IsoPrime 100 IRMS (IsoPrime) after removing inorganic C by fumigation with HCl and subsequent neutralization over NaOH pellets (modified from ref. ^65^). Rates of CH_4_ oxidation at 4 and 20 °C as well as the effect of different moisture conditions and labile C addition were studied in laboratory incubations. Details on soil analyses are provided in Supplementary Methods.

### Nutrients and dissolved organic carbon

Amounts of the plant-available N forms NH_4_^+^ and NO_3_^−^ at Trail Valley Creek were determined using plant–root simulator (PRS) probes (Western Ag Innovations). Probes were installed next to each manual chamber flux collar of lichen, shrub and tussock and left in place for four weeks, after which the next set of probes was installed at the same location. We determined plant nutrient supply for three time periods (June, July and August) during 2019. Probes were returned to the manufacturer for analysis. To determine nutrient turnover rates, that is, use of macronutrients by microbes, nutrients were extracted for the intensively studied sites (Trail Valley Creek, Kilpisjärvi Palsa II and Kilpisjärvi upland forest) using 1 M KCl for NH_4_^+^ and deionized water for the ions NO_3_^−^, nitrite, phosphate, SO_4_^2−^ and chloride. Soil extractions were repeated after four weeks of storing soil samples in the dark at +4 °C. Extracts were stored frozen until analysis. NH_4_^+^ concentrations were determined spectrophotometrically as described previously^18^. Ions were analysed by ion chromatography (Dionex ICS-2100 and AS-DV, Thermo Scientific). Dissolved organic carbon (DOC) and dissolved TN were determined on a TOC analyser with TN measurement unit and autosampler (TOC-L, TNM-L and ASI-L, Shimadzu). Dissolved organic nitrogen was determined by subtracting the amounts of inorganic N forms from TN concentrations. For nutrient and DOC analyses, the final sample concentrations were calculated on the basis of a standard series as well as blanks. Net ammonification was calculated as the difference in NH_4_^+^ concentrations between the second (after incubation) and first (initial) extraction divided by the number of days of the incubation time^66^. Net nitrification was determined similarly but based on the difference of NO_3_^−^ concentrations.

### Auxiliary data collection

Accompanying manual chamber flux measurements, thaw depth, surface soil moisture (0–6 cm depth), air and soil temperature (5 cm depth) were recorded next to each flux collar concurrent with manual chamber flux measurements (Supplementary Methods). For continuous measurements of soil temperature, volumetric water content and soil oxygen concentration to accompany automated chamber measurements at Trail Valley Creek, we installed sensors at three depths (10, 20 and 30 cm) in one soil profile per vegetation type (lichen, shrub and tussock) using soil moisture probes (CS650L, Water Content Reflectometer Plus with 30 cm long rods, Campbell Scientific) and oxygen probes (Yuasa KE-25, Figaro Engineering). Oxygen sensors were calibrated in ambient air and waterproofed by placing them in a silicon tube sealed with rubber septum. Meteorological variables were collected at nearby automated weather stations located within a 50 m radius of the automated chamber set-up. Meteorological measurements included air temperature, relative humidity, wind speed, rainfall, PAR and air pressure and details are provided in Supplementary Methods.

### Statistical analyses

Statistical analyses were performed in R v.4.2.2 (ref. ^67^). For high-resolution, automated chamber data we report effect sizes rather than *P* values to assess differences between vegetation types and diel flux rates. One transparent chamber with large CH_4_ uptake (−2.3 ± 0.13 mgCH_4_ m^−2^ d^−1^ during August 2021) was removed for Fig. 1 and Supplementary Fig. 4 to not distort the calculation of mean and standard error (lichen, *n* = 6; shrub, *n* = 5; tussock, *n* = 6), whereas all chambers were included in other analyses. We applied an RF model approach, well suited for large datasets involving non-normal and nonlinear distribution and relationships^68^. RF analysis was performed using the R package randomForest^69^ and one model was created for all vegetation types (including only fluxes <0 mgCH_4_ m^−2^ h^−1^), as well as three separate models for individual vegetation types. Data were further split into two datasets (Supplementary Table 8):

1. Flux data from 2019 and 2021, with a smaller set of environmental variables (soil temperature and moisture only measured in the surface soil; six predictors).
2. Flux data from 2021, during which a larger set of environmental variables were measured (including soil temperature and moisture in the soil profile down to 30 cm; 18 predictors). This model included some highly correlated predictors (Spearman correlation coefficient >0.7).

We used 500 trees to construct the random forests (ntree = 500) and the number of variables tried at each split was determined individually for each model using function tuneRF. Variable importance was assessed by the average increase in node purity of the regression trees. Further RF models were created for each chamber to explore the relative importance of the two established controls on CH_4_ uptake, soil temperature and soil moisture^2,26,70,71^, as well as the additional explanatory power of ER. RF models were created for each replicate chamber to control for microsite heterogeneity, split by early, peak and late summer. To ensure robustness of the RF analysis and to identify if the dominant predictors change over different temporal scales as observed for CH_4_ emissions by ref. ^72^, we repeated model 1 (hourly measured fluxes) for daily and weekly aggregated data, as well as data split by daytime versus night time. On the basis of the RF results, we applied transfer entropy analysis to detect lagged interactions between fluxes measured by automated chambers and environmental variables, in particular temperature. Details on the RF models and transfer entropy analysis are provided in Supplementary Methods.

For manual chamber flux data, we conducted significance tests (two-tailed) to identify differences between sites, land cover and vegetation type. For comparison between vegetation types (lichen and shrub) between all sites, as well as differences between all Canadian compared to all Finnish sites, we applied Welch’s two-sample *t*-test for non-normal distribution (Supplementary Table 10). To test for differences between all sites and land covers, we performed a Kruskal–Wallis test for non-parametric data followed by pairwise multiple comparison using Dunn’s test with R packages FSA^73^, multcompView^74^ and reshape^75^.

### Reporting summary

Further information on research design is available in the Nature Portfolio Reporting Summary linked to this article.

## Online content

Any methods, additional references, Nature Portfolio reporting summaries, source data, extended data, supplementary information, acknowledgements, peer review information; details of author contributions and competing interests; and statements of data and code availability are available at 10.1038/s41558-023-01785-3.

### Supplementary information

Supplementary Information Supplementary Figs. 1–15, Tables 1–14 and Methods.

Reporting Summary

## Data Availability

The main flux datasets generated within this study are publicly available on the PANGAEA data repository (10.1594/PANGAEA.953120)^76^. Further, auxiliary data are available from the authors upon reasonable request.

## Code availability

The code used to process automated chamber flux data is available on GitHub at https://github.com/znesic/Voigt2023_CH4_uptake ref. ^77^. The R code for creation of RF models has been posted on Zenodo (10.5281/zenodo.8152386)^78^.
