# Supplementary material for: Arctic soil methane sink increases with drier conditions and higher ecosystem respiration
Source: Nat Clim Chang. 2023 Aug 31;13(10):1095–104. doi: 10.1038/s41558-023-01785-3 (PMC10550823; doi:10.1038/s41558-023-01785-3)
Supplement: Supplementary file 1 — Supplementary Figs. 1–15, Tables 1–14 and Methods. [file 41558_2023_1785_MOESM1_ESM.pdf]

nature climate change

Article https://doi.org/10.1038/s41558-023-01785-3

Arctic soil methane sink increases with drier conditions and higher ecosystem respiration

In the format provided by the authors and unedited

Table of Contents

Supplementary Figures

Supplementary Fig. 1: Overview of the automated chamber set-up at Trail Valley Creek.
Supplementary Fig. 2: Soil temperature and moisture profiles at Trail Valley Creek.
Supplementary Fig. 3: Diel cycles of fluxes and environmental conditions at Trail Valley Creek.
Supplementary Fig. 4: Diel variability of methane fluxes in early and late summer at Trail Valley Creek.
Supplementary Fig. 5: Relative importance of abiotic variables on methane fluxes at Trail Valley Creek.
Supplementary Fig. 6: Seasonal variation in thaw depth at Trail Valley Creek.
Supplementary Fig. 7: Relative importance of temperature, soil moisture, and ecosystem respiration.
Supplementary Fig. 8: Methane concentration in the soil profile.
Supplementary Fig. 9: Soil profiles of carbon and nitrogen at Trail Valley Creek.
Supplementary Fig. 10: Flux processing of automated chamber data.
Supplementary Fig. 11: Stable carbon isotope data.
Supplementary Fig. 12: Principal component analysis.
Supplementary Fig. 13: Transfer entropy analysis of fluxes and surface soil temperature.
Supplementary Fig. 14: Transfer entropy analysis of fluxes and soil temperature in 10 cm.
Supplementary Fig. 15: Transfer entropy analysis of fluxes and photosynthetically active radiation.

Supplementary Tables

Supplementary Table 1: Overview of measurement sites.
Supplementary Table 2: Sites, sampling times, and number of observations included in this study.
Supplementary Table 3: Meteorological conditions during manual chamber measurements at the Canadian and Finnish sampling locations.
Supplementary Table 4: Methane fluxes measured with automated chambers at Trail Valley Creek Creek.
Supplementary Table 5: Climate data for Inuvik.
Supplementary Table 6: Diel peaks in methane uptake at Trail Valley Creek.
Supplementary Table 7: Over- and underestimation of daily methane fluxes at Trail Valley Creek.
Supplementary Table 8: Overview table of Random Forest models.
Supplementary Table 9: Fluxes and environmental conditions measured at Trail Valley Creek and other sites across the Arctic.
Supplementary Table 10: Methane fluxes measured with manual chambers at Trail Valley Creek and other sites across the Arctic.
Supplementary Table 11: Upscaled growing season methane uptake.
Supplementary Table 12: Technical specifications of greenhouse gas analyzers used in this study.
Supplementary Table 13: Linear-mixed-effects models.
Supplementary Table 14: Model comparison.

Supplementary Methods

References for Supplementary Material

Supplementary Figures

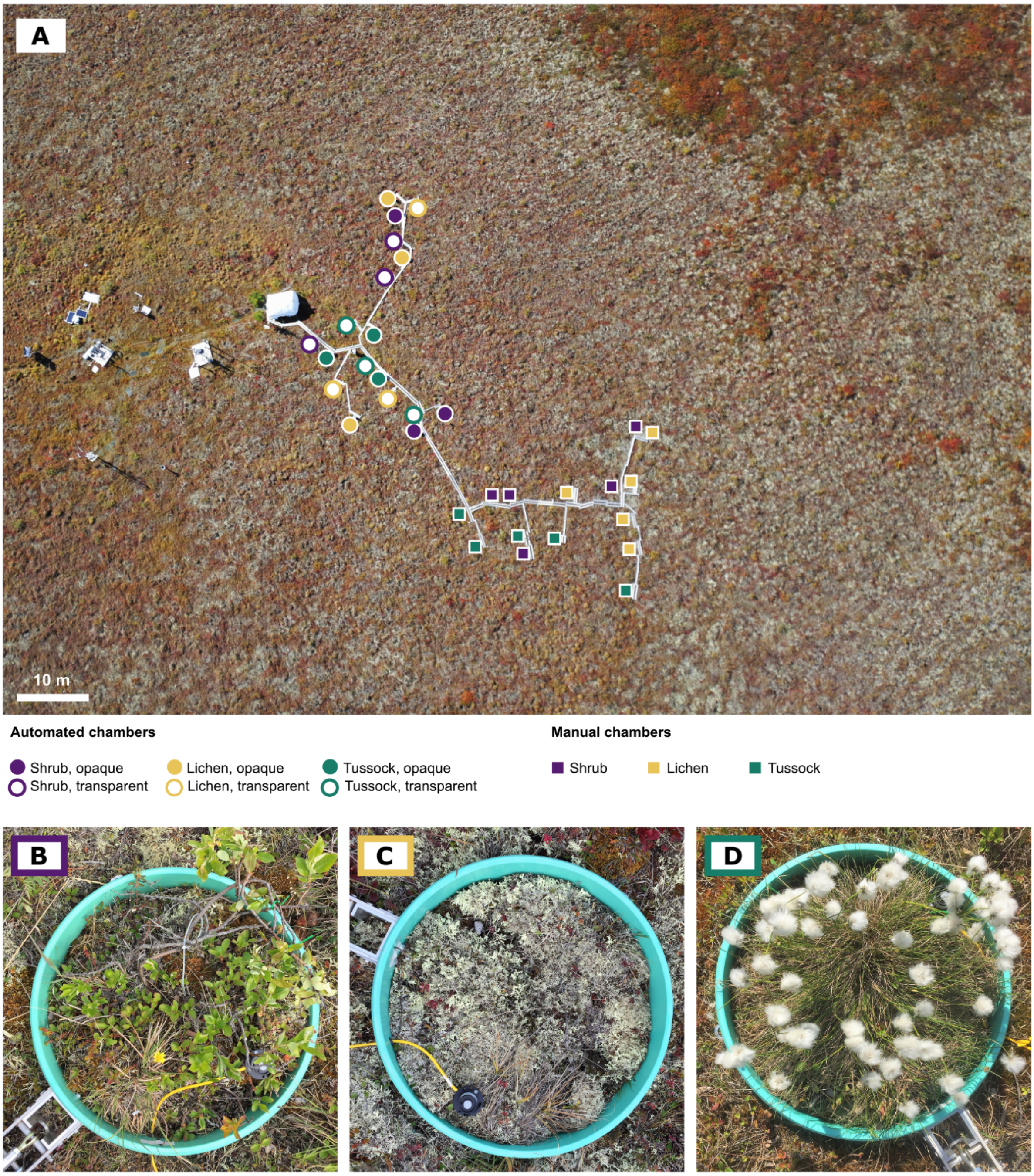

Supplementary Fig. 1: Overview of the automated chamber set-up at Trail Valley Creek. Figure shows locations of the automated and manual chamber flux measurements on upland tundra (**A**), as well as a typical Shrub (**B**), Lichen (**C**), and Tussock (**D**) collars measured with automated chambers. Photos were taken at the end of August (A) and at the end of June (B-D).

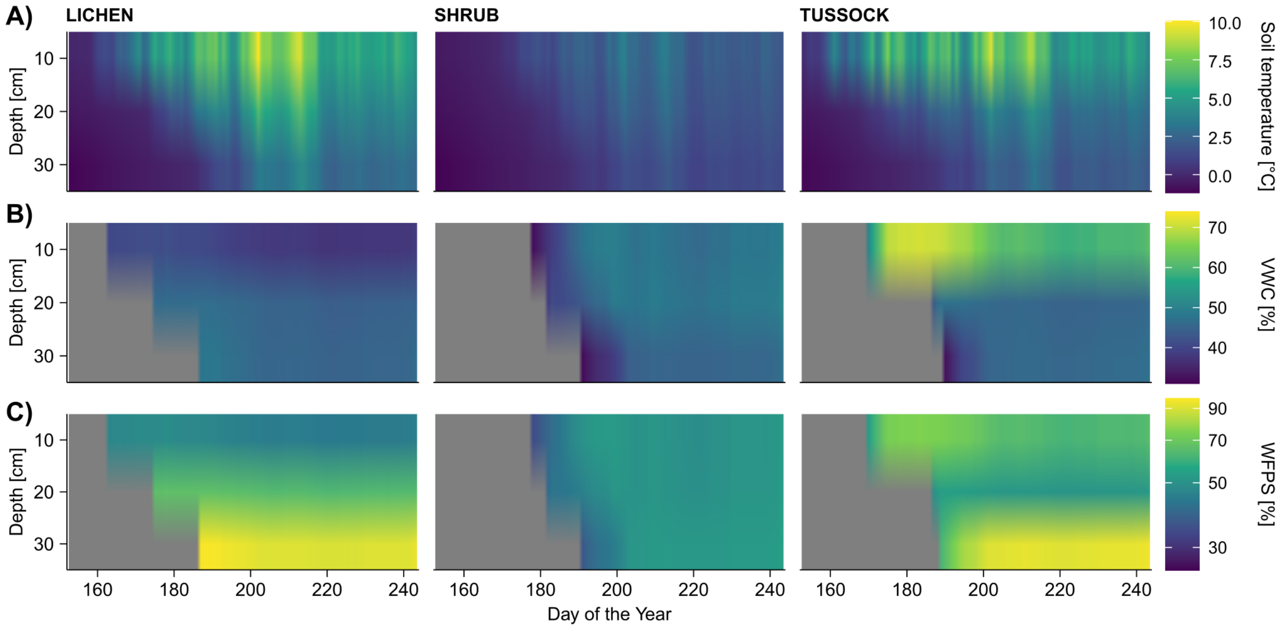

Supplementary Fig. 2: Soil temperature and moisture profiles at Trail Valley Creek. Heatmaps of soil temperature (**A**), volumetric water content (VWC; **B**), and soil water-filled pore space (WFPS; **C**) measured continuously (30 min intervals) at depths of 10 cm, 20 cm, and 30 cm in the soil profiles of Lichen, Shrub and Tussock at Trail Valley Creek during year 2021. Grey colour indicates no data (frozen soils).

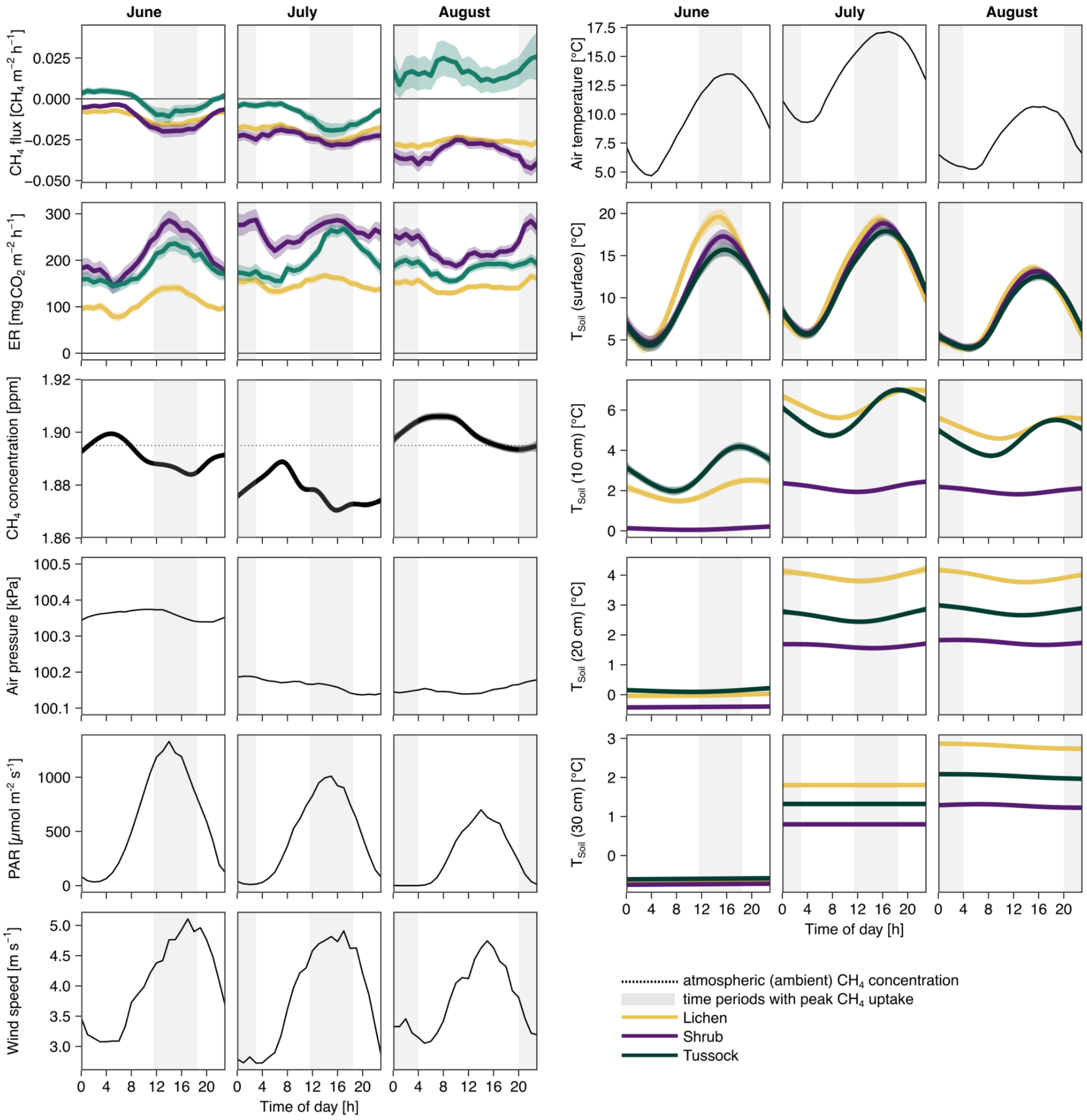

Supplementary Fig. 3: Diel cycles of fluxes and environmental conditions at Trail Valley Creek. Diel (24 h) variation in starting point methane (CH_4) concentrations at the time of chamber closure, CH_4 fluxes, ecosystem respiration (ER) as well as auxiliary variables at Trail Valley Creek, split by early (June), peak (July), and late growing season (August), including the spring green-up and autumn senescence periods. This figure complements Fig. 2 in the main text. Data for fluxes show means with confidence intervals of each vegetation type based on hourly values measured during 2019 and 2021. Negative values denote CH_4 uptake. Note that the daytime to nighttime difference in ambient CH_4 concentrations is <0.02 ppm, and higher ambient concentrations do not match peaks in atmospheric CH_4 uptake indicating the larger uptake is not driven by higher atmospheric CH_4.

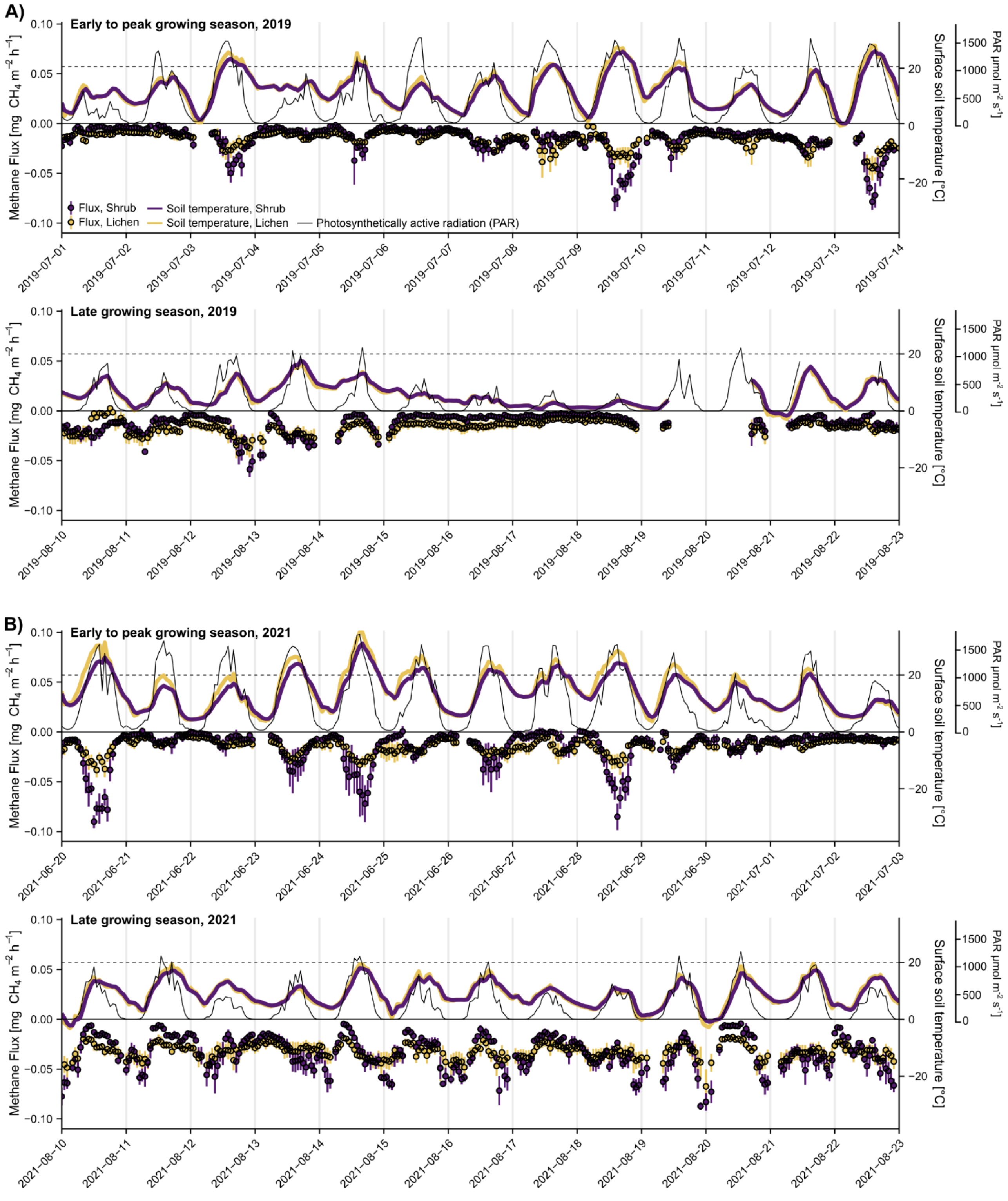

Supplementary Fig. 4: Diel variability of methane fluxes in early and late summer at Trail Valley Creek. Example of the diel variability in methane (CH_4) fluxes over 14-day periods in early to peak vs. late growing season during the measurement seasons 2019 (**A**) and 2021 (**B**) for Lichen and Shrub, as well as soil temperature (0–12 cm) and photosynthetically active radiation (PAR; measured as photon flux density). Fluxes were measured hourly with automated chambers. Data show microsite means ± standard error of transparent and opaque chambers combined. One transparent chamber with large CH_4 uptake was removed to not distort the calculation of mean and standard error (Lichen: n = 6; Shrub: n = 5). Negative values denote CH_4 uptake. Large diel variability was often observed on warm, sunny days as indicated by the dotted line at 20 °C. Differing time periods for early to peak growing season are shown due to a later start of measurements in 2019 and data gaps during July in 2021.

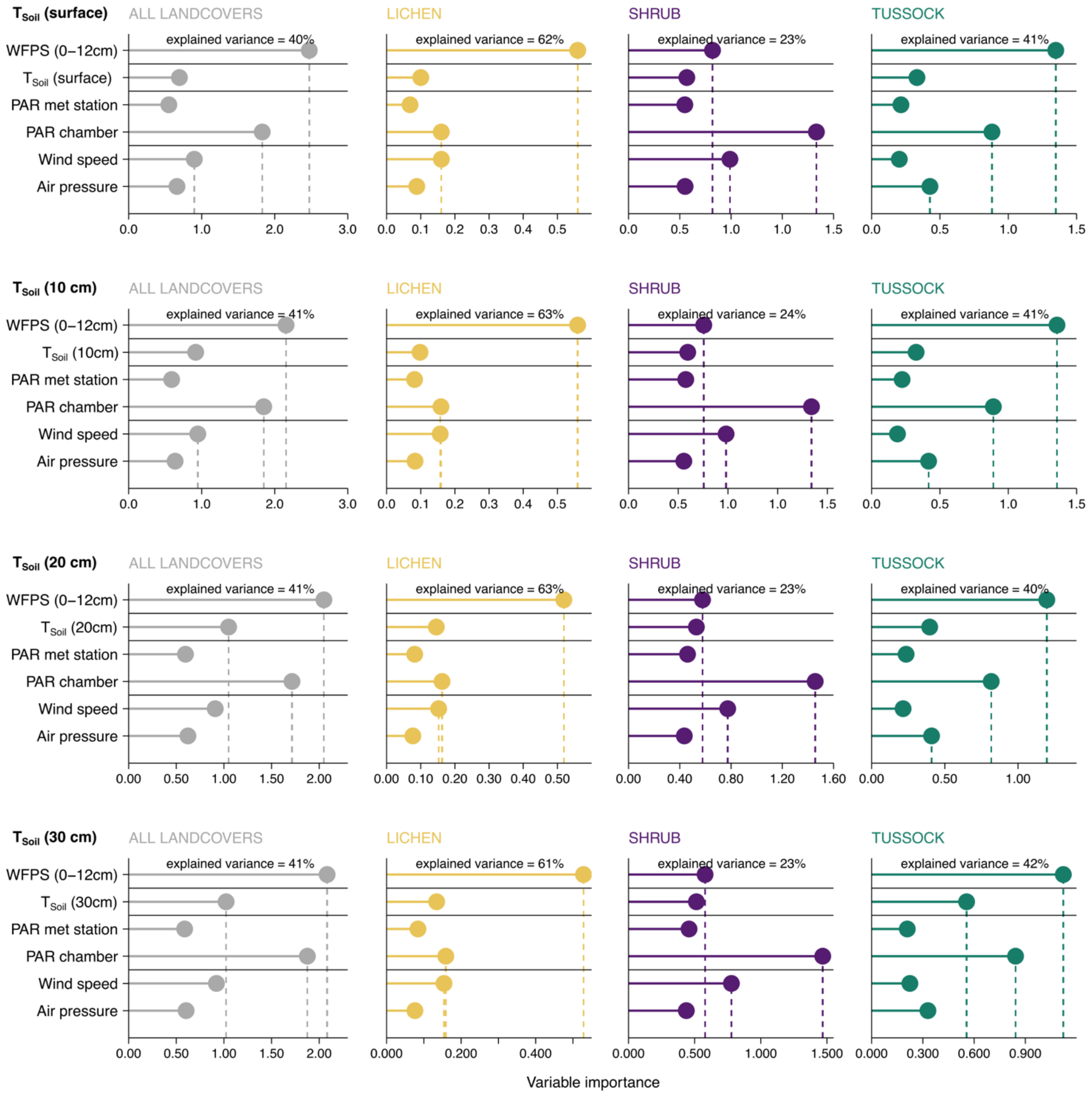

Supplementary Fig. 5: **Relative importance of abiotic variables on methane fluxes at Trail Valley Creek.** Relative importance of abiotic variables on hourly measured methane (CH_4) fluxes determined with a Random Forest (RF) model for Lichen, Shrub, and Tussock measured with automated chambers for a subset of data collected during comparatively dry conditions during 2021 (DOY 182-243). The figure shows that even during periods with mostly favourable soil moisture conditions, WFPS frequently had a higher relative importance on CH_4 fluxes compared to soil temperatures at various depths. The RF models were created using soil temperatures measured in different depths. The RF model for all vegetation types (18 chambers) includes only CH_4 uptake, whereas for the individual vegetation types (6 chambers per vegetation type), all fluxes were included. This includes mainly CH_4 uptake for Lichen and Shrub, but occasional emissions from Tussock. Variables are grouped from top to bottom into 'moisture-related', 'temperature-related', 'PAR-related', and 'other meteorological variables', and the three most important variables are indicated by vertical dashed lines. Note that 'PAR chamber' considers opaque and transparent chambers (PAR = 0 µmol m^{-2} s^{-1} in opaque chambers), whereas PAR met station are actual site PAR data. T_{Soil} = soil temperature, WFPS = soil water-filled pore space.

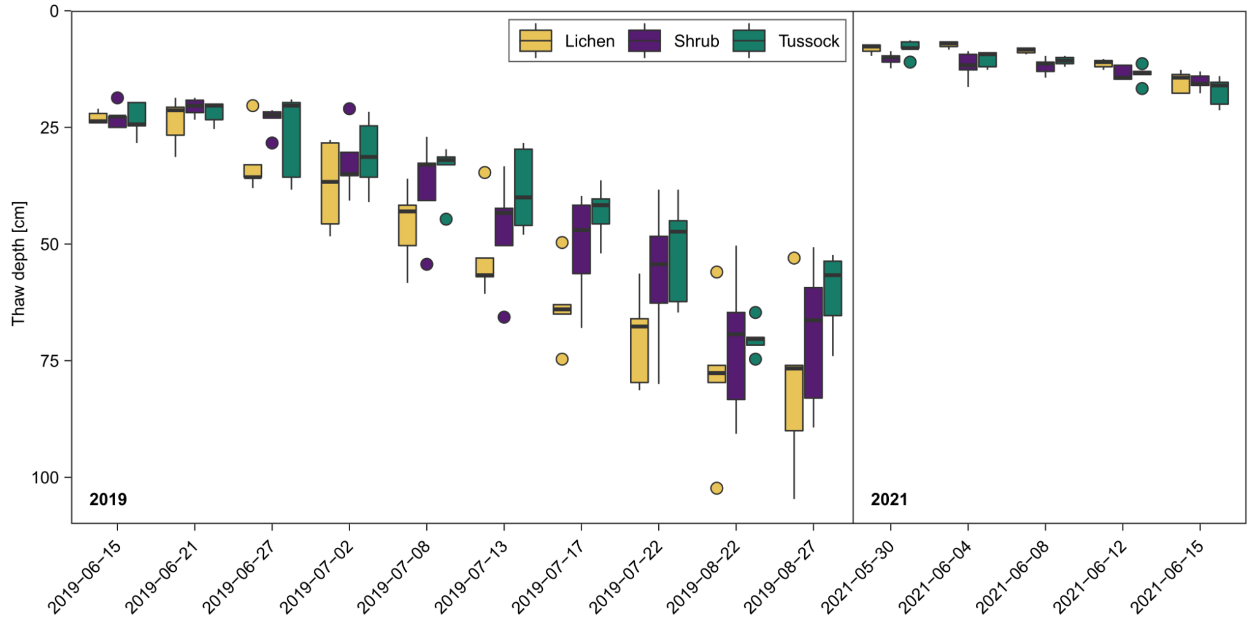

Supplementary Fig. 6: Seasonal variation in thaw depth at Trail Valley Creek. Thaw depths were measured at the manual chamber flux locations at Trail Valley Creek for Lichen, Shrub and Tussock during 2019 and 2021. Boxplots are based on n = 5 replicate collars per vegetation type and show median (thick, black line), upper and lower quartile (boxes), the highest and lowest values (black vertical lines), and outliers (circles).

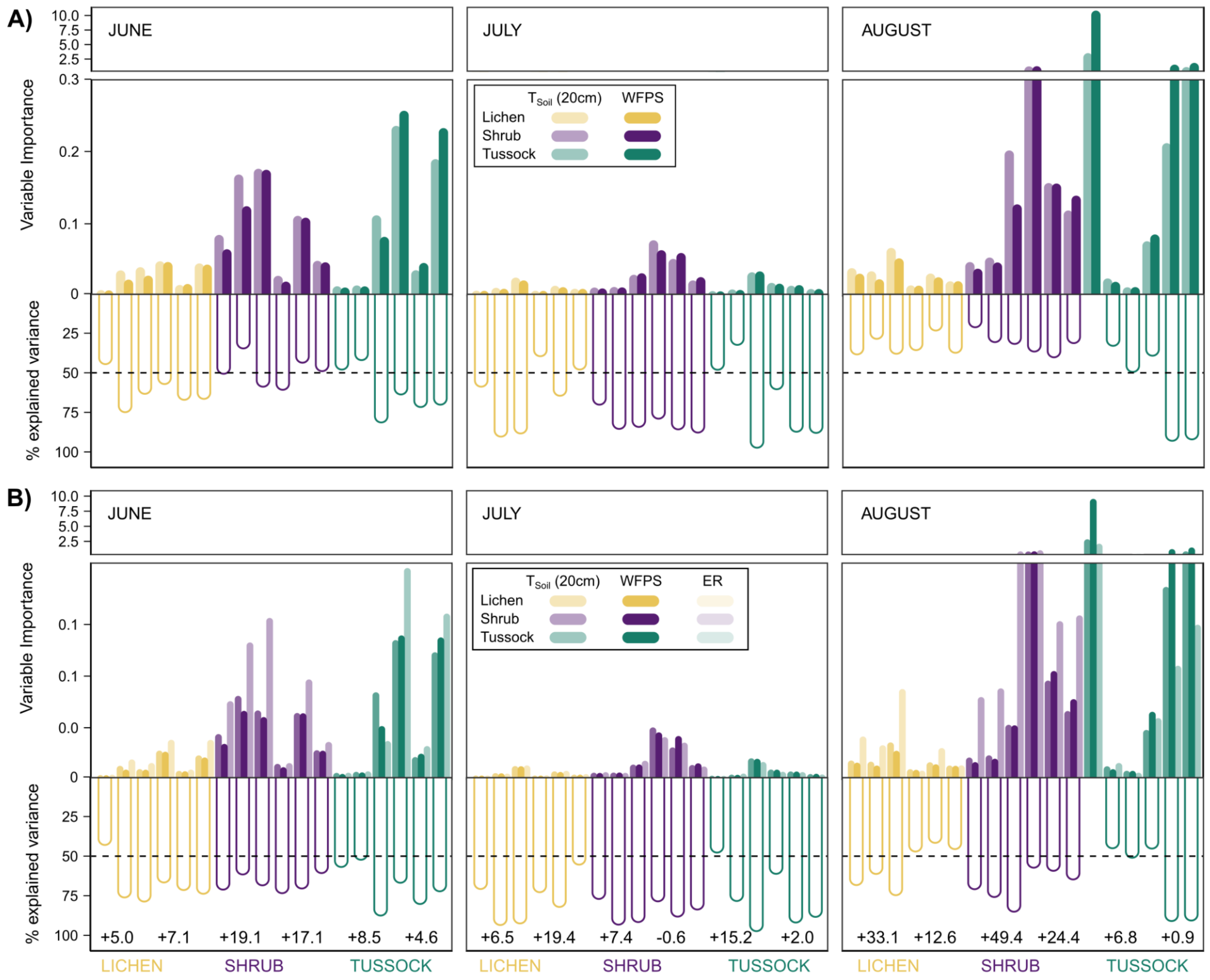

Supplementary Fig. 7: Relative importance of temperature, soil moisture, and ecosystem respiration. Relative importance of the two important predictors, soil temperature at the 20-cm depth and soil water-filled pore space (WFPS), on methane (CH_4) fluxes measured with automated chambers at Trail Valley Creek for Lichen, Shrub, and Tussock (**A**), as well as the additional percentage of variance explained by ecosystem respiration (ER; **B**). Data were split by chamber (18 chambers) and month. The additional percentages of variance explained by ER and PAR are indicated as the % change of model fit compared to fitting the model to only soil temperature and WFPS. Note that soil temperature at 20 cm was only available for year 2021, causing a larger uncertainty in model results particularly during July compared to models using surface soil temperature (Fig. 4).

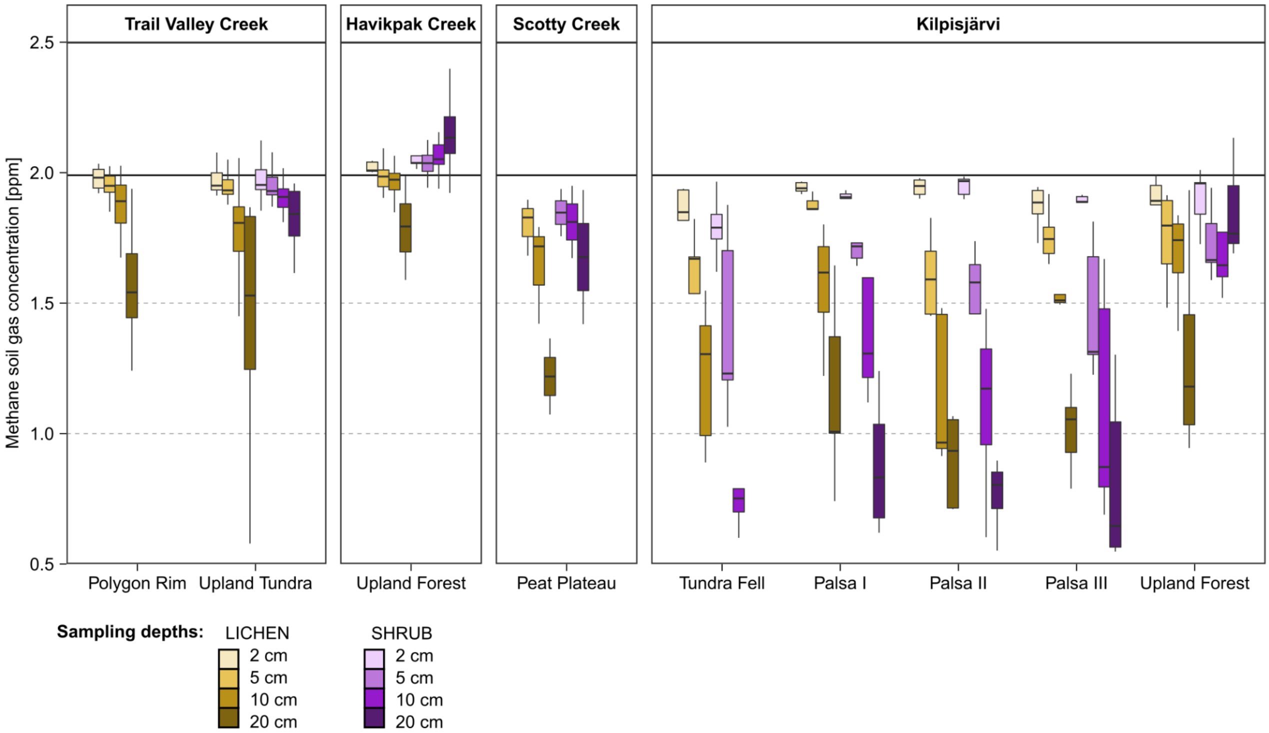

Supplementary Fig. 8: Methane concentration in the soil profile. Methane concentrations in the soil profile at depths of 2 cm, 5 cm, 10 cm, and 20 cm at manual chamber flux locations for Lichen and Shrub. Due to shallow soils, no samples were taken at the 20-cm depth in Tundra Fell. The solid black horizontal line indicates current atmospheric methane concentration (1.9 ppm). Boxplots show median (thick, black line), upper and lower quartile (boxes), and the highest and lowest values (black vertical lines). The number of replicates (n) is the same as used in flux measurements (see Fig. 5 for details).

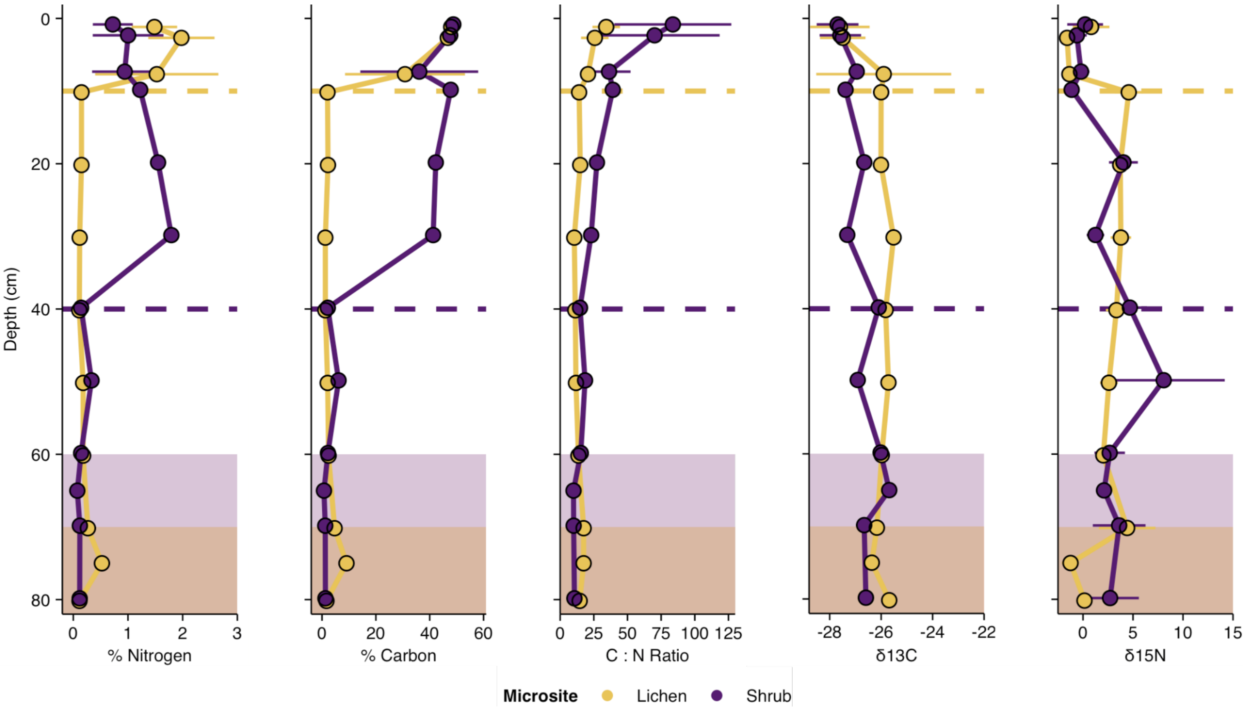

Supplementary Fig. 9: Soil profiles of carbon and nitrogen at Trail Valley Creek. Soil profiles of nitrogen (N) and carbon (C) content, C:N ratio, as well as stable isotopic signals of $\delta^{13}C$ and $\delta^{15}N$ in soil cores collected at Lichen and Shrub at Trail Valley Creek. Data show means ± standard deviation for one soil core per vegetation type analyzed from 3 replicate samples per depth, except for 0 – 10 cm where soil samples were additionally collected from the manual chamber flux locations (n = 5 per vegetation type). Light purple and brown shaded areas indicate permafrost for Shrub and Lichen, respectively, and dashed lines indicate the organic layer depths. Note that the organic layer depth under Shrub for the manual chamber flux measurement locations was shallower (typically < 20 cm; Supplementary Table 9).

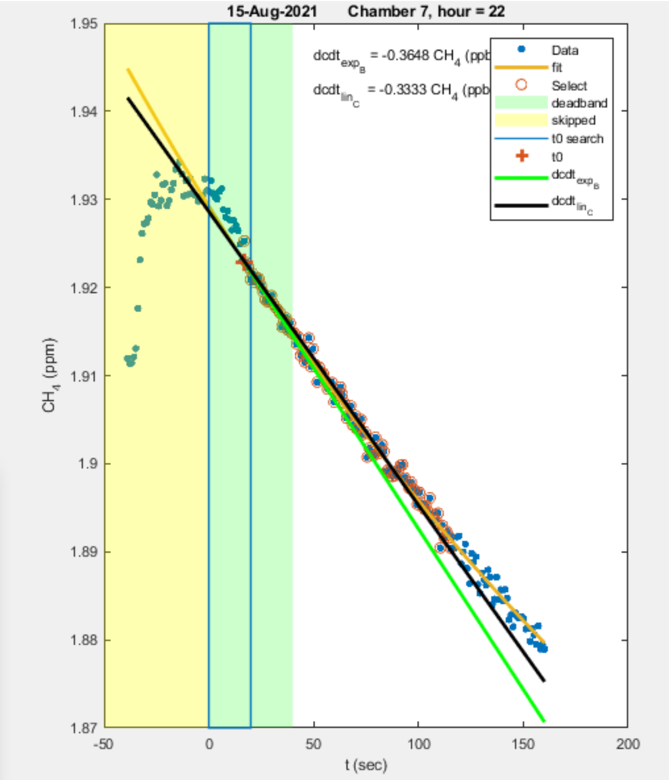

Supplementary Fig. 10: **Flux processing of automated chamber data.** Screenshot of flux determination in MATLAB code for processing of automated chamber data measured with Los Gatos Research Enhanced Performance greenhouse gas analyzer at Trail Valley Creek. Plot shows points skipped at the beginning of the measurement, which include concentrations from the previous chamber and switch to ambient concentrations (yellow), a deadband that includes the time of chamber lid closure (green), as well as a period of 20 seconds within the deadband, tested individually as a starting point for the slope (t_0 search) and the determined t0 starting point (red cross). Plot shows the slopes calculated with both linear and exponential fits.

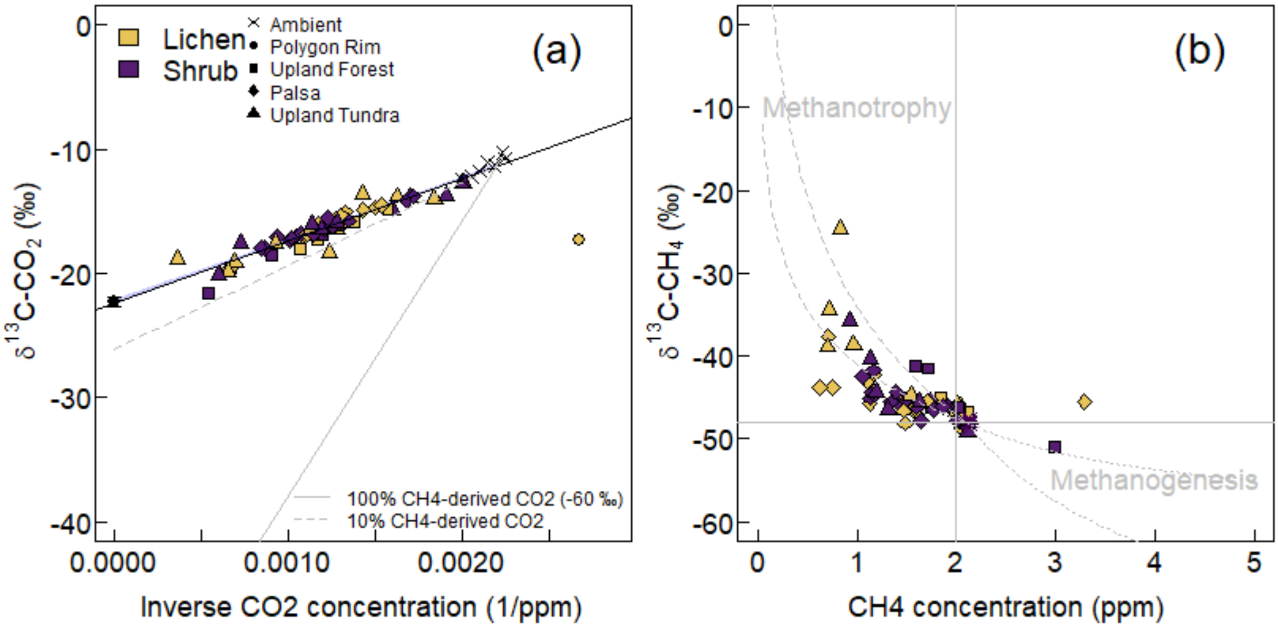

Supplementary Fig. 11: **Stable carbon isotope data.** Stable carbon isotope values of soil and ambient CO_2 (a) and methane (CH_4; b) relative to soil gas concentrations. Samples analyzed for isotope ratios were collected at the 10-cm depth during June 2021 at Trail Valley Creek and during August 2021 at all other sites. a) represents a Keeling Plot, finding that all measured data points except for one outlier fall on a single line, indicating that soils at all sites produced CO_2 with a constant δ^{13}C-CO_2 value. This source value can be estimated as the intercept of the regression line (-22.3±0.4 ‰), which is slightly δ^{13}C enriched relative to the value expected for soil respiration (ca. -27 %). In comparison, CO_2 derived from CH_4 oxidation would be depleted due to the low δ^{13}C of the source CH_4 (atmospheric CH_4: ca. -48 ‰, microbial CH_4: <-55 ‰), and which is further depleted by ca. 10–20 ‰ during CH_4 oxidation. The figure therefore shows the trend line expected if all soil CO_2 were derived from CH_4 oxidation assuming a source isotope value of -60 ‰, and if 10 % of soil CO_2 were derived from CH_4 oxidation. b) shows CH_4 isotope values plotted against concentrations to compare the measured values to the trends expected for the oxidation of atmospheric CH_4 and mixing of atmospheric CH_4 with microbially produced CH_4 in soil.

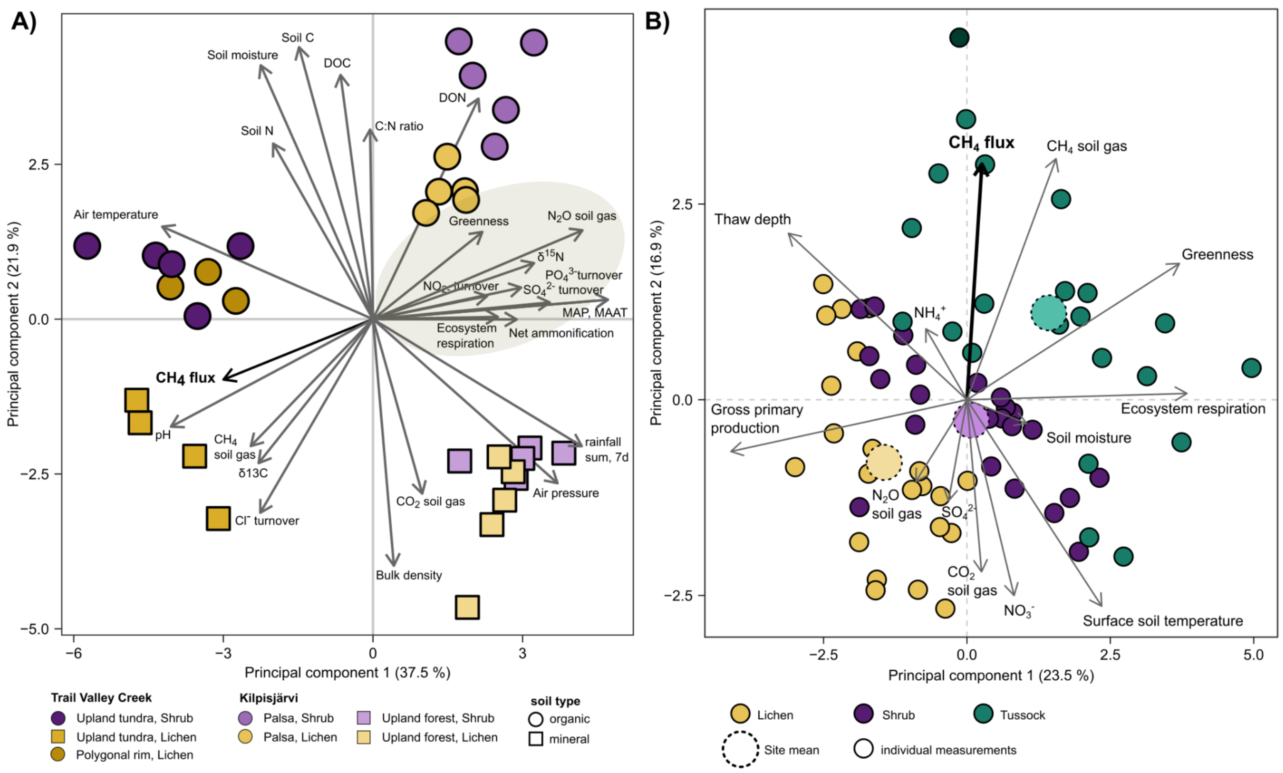

Supplementary Fig. 12: Principal component analysis. Principal component analysis (PCA) of methane (CH_4) fluxes and the abiotic and biotic controls measured in the western Canadian Arctic (Trail Valley Creek) and Finnish Lapland (Kilpisjärvi) (**A**), and PCA for three vegetation types at Trail Valley Creek (**B**). Data for PCA were scaled and centered, and in **A**) includes only CH_4 uptake (i.e., negative fluxes), whereas in **B**) data include CH_4 uptake and emissions. Shaded area in **A**) indicates variables with close, positive association with CH_4 uptake in the PCA. Note that the arrow for 'CH_4 flux' points towards lower uptake (i.e., higher flux). Organic soils: organic soil layer on top of mineral horizon is >30 cm. Variables: DOC = dissolved organic carbon, DON = dissolved organic nitrogen, C:N ratio = soil carbon to nitrogen ratio, Greenness = vegetation greenness in the flux collar, NO_2^- = nitrite, SO_4^{2-} = sulfate, PO_4^{3-} = phosphate, Cl^- = chloride, NH_4^+ = ammonium, NO_3^- = nitrate, N_2O = nitrous oxide, CO_2 = carbon dioxide, rainfall sum, 7d = amount of rainfall during the previous seven days, MAP = mean annual precipitation, MAAT = mean annual air temperature.

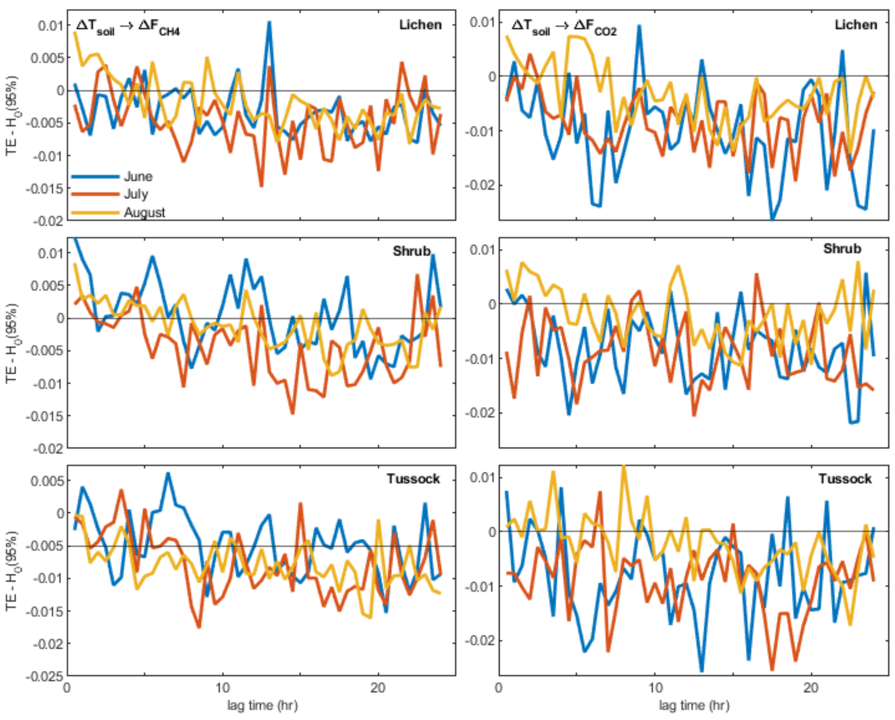

Supplementary Fig. 13: **Transfer entropy analysis of fluxes and surface soil temperature.** Lagged effects of surface soil temperature on methane (F_{CH4}) and ecosystem respiration (F_{CO2}) fluxes estimated from transfer entropy (positive values indicate significant effects with a 95 % confidence interval).

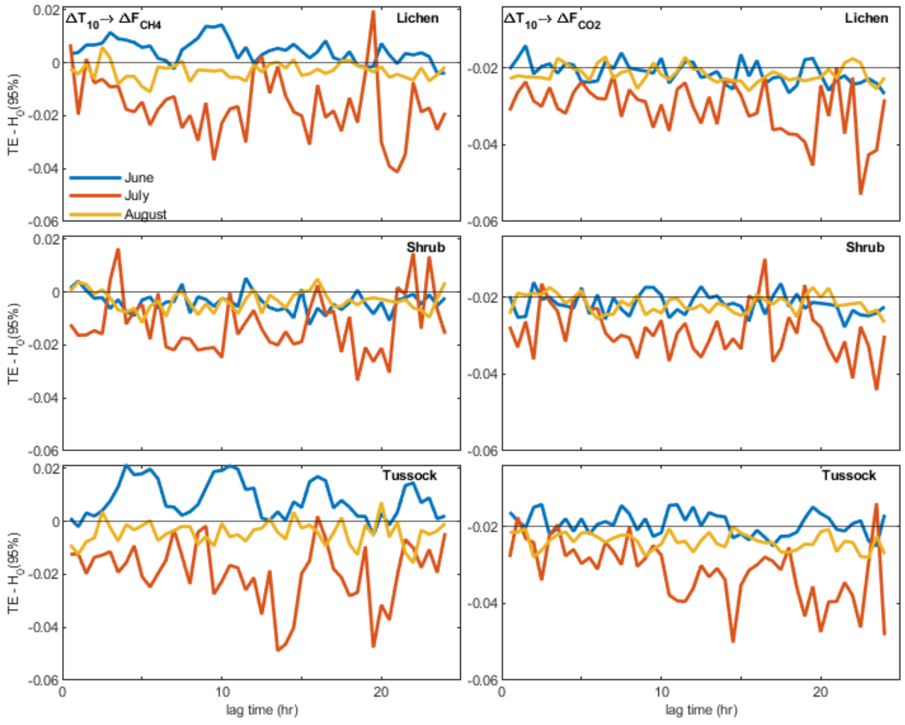

Supplementary Fig. 14: **Transfer entropy analysis of fluxes and soil temperature in 10 cm.** Lagged effects of soil temperature at the 10-cm depth on methane (F_{CH4}) and ecosystem respiration (F_{CO2}) fluxes estimated from transfer entropy (positive values indicate significant effects with a 95 % confidence interval). Note that temperature at this depth was only measured during the year 2021 and results, particularly for July, are therefore connected to larger uncertainty than using surface soil temperature.

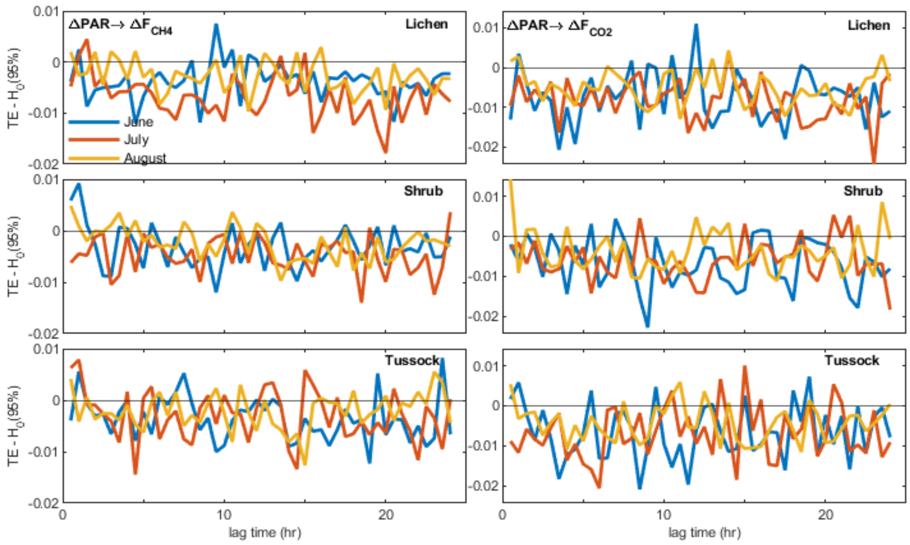

Supplementary Fig. 15: **Transfer entropy analysis of fluxes and photosynthetically active radiation.** Lagged effects of photosynthetically active radiation (PAR) on methane (F_{CH4}) and ecosystem respiration (F_{CO2}) fluxes estimated from transfer entropy (positive values indicate significant effects with a 95% confidence interval).

Supplementary tables

Supplementary Table 1: Overview of measurement sites. Elevation, mean annual air temperature (MAAT) and mean annual precipitation (MAP) measured at Inuvik (68°18’15.000" N, 133°28’58.000" W) for Trail Valley Creek and Havikpak Creek, at Fort Simpson (61°45’37.000" N, 121°14’12.000" W) for Scotty Creek, and at Enontekiö Kilpisjärvi station (69°02’20.58" N, 20°48’49.644" E) for sites in Finnish Lapland. *Note that Trail Valley Creek is located 45 km north of Inuvik in open tundra and MAAT at the site is lower than at Havikpak Creek and precipitation amounts likely differ between the two sites. Data sources: Environment and Climate Change Canada (Canadian sites, 1981–2010); Finnish meteorological Institute (Finnish sites, 1991–2020).

Site	Ecoregion	Coordinates	Permafrost zone	Elevation *(m)*	MAAT *(°C)*	MAP *(mm)*
Trail Valley Creek	western Canadian Arctic	68°44’32.496" N 133°29’55.824" W	continuous	68	-8.2*	241*
Havikpak Creek	western Canadian Arctic	68°19’14.9484" N 133°31’04.872" W	continuous	68	-8.2	241
Scotty Creek	western Canadian Arctic	61°18’29.2176" N 121°18’01.3392" W	discontinuous / sporadic	169	-2.8	388
Kilpisjärvi	European Arctic / Finnish Lapland	68°51’54.2886" N 21°06’24.1434" E	sporadic	85	-1.7	546

Supplementary Table 2: **Sites, sampling times, and number of observations included in this study.** Data collected during manual chamber measurement campaigns in the Western Canadian and Finnish Arctic during the growing seasons of 2018, 2019, and 2021.

Site	Time period	Instrument	Frequency	Obs.	Microsite, replication	Soil
**Trail Valley Creek**	June 21–Aug 22, 2019	G4301 Picarro GasScouter	1–2 times per week	96	**Upland tundra**	
					Shrub (n = 5)	mineral
					Lichen (n = 5)	mineral
					**Polygonal tundra (Polygon rim)**	
					Lichen (n = 3)	organic
**Havikpak Creek**	June 20, 2021 [Soil gas: Aug 31, 2018, June 9–June 20, 2021]	G4301 Picarro GasScouter	once	15	**Upland forest**	
					Shrub (n = 10)	mineral
					Lichen (n = 5)	mineral
**Scotty Creek**	Sept 12, 2018	LGR U-GGA-915 Ultraportable	once	5	**Peat Plateau (forested)**	
					Shrub (n = 2)	organic
					Lichen (n = 3)	organic
**Finnish Lapland (Kilpisjärvi)**	Aug 17–Aug 26, 2021	G4301 Picarro GasScouter	Site "Palsa III" twice, all others once	60	**Upland forest**	
					Shrub (n = 5)	mineral
					Lichen (n = 5)	mineral
					**Palsa I–III**	
					Shrub (n = 5)	organic
					Lichen (n = 5)	organic
					**Upland tundra (tundra fell)**	
					Shrub (n = 5)	mineral
					Lichen (n = 5)	mineral

Supplementary Table 3: **Meteorological conditions during manual chamber measurements at the Canadian and Finnish sampling locations.** Data show mean ± standard deviation.

Site	Land cover and vegetation type		Air temperature [°C]	Volumetric water content [%]	Water-filled pore space [%]
Trail Valley Creek	Upland tundra	Lichen	16.3 ± 4.5	31 ± 6	34 ± 6
Trail Valley Creek	Upland tundra	Shrub	16.2 ± 4.8	29 ± 5	30 ± 6
Trail Valley Creek	Polygonal tundra (polygon rim)	Lichen	13.5 ± 3.8	35 ± 7	37 ± 7
Havikpak Creek	Upland forest	Lichen	22.5 ± 0.3	2 ± 3	2 ± 3
Havikpak Creek	Upland forest	Shrub	21.8 ± 0.4	13 ± 13	14 ± 14
Scotty Creek	Peat plateau	Lichen	7.7 ± 0.1	11 ± 2	n.d. ± n.d.
Scotty Creek	Peat plateau	Shrub	7.5 ± 0.6	7 ± 1	n.d. ± n.d.
Kilpisjärvi	Palsa I	Lichen	8.2 ± 0.0	45 ± 6	n.d. ± n.d.
Kilpisjärvi	Palsa I	Shrub	8.2 ± 0.0	36 ± 9	n.d. ± n.d.
Kilpisjärvi	Palsa II	Lichen	13.1 ± 0.1	43 ± 12	47 ± 14
Kilpisjärvi	Palsa II	Shrub	13.1 ± 0.0	36 ± 8	38 ± 9
Kilpisjärvi	Palsa III	Lichen	11.0 ± 2.4	35 ± 12	38 ± 13
Kilpisjärvi	Palsa III	Shrub	11.0 ± 2.4	31 ± 13	32 ± 14
Kilpisjärvi	Upland forest	Lichen	7.3 ± 0.6	25 ± 7	34 ± 14
Kilpisjärvi	Upland forest	Shrub	7.3 ± 0.6	19 ± 5	23 ± 5
Kilpisjärvi	Upland tundra (tundra fell)	Lichen	7.1 ± 0.1	30 ± 3	n.d. ± n.d.
Kilpisjärvi	Upland tundra (tundra fell)	Shrub	7.1 ± 0.1	28 ± 7	n.d. ± n.d.

Supplementary Table 4: Methane fluxes measured with automated chambers at Trail Valley Creek. Table shows mean and median methane (CH_4) flux values measured during 2019 and 2021 split by month, as well as the lower and upper confidence intervals (CI; 95% confidence level), number of replicate collars per land cover and number of observations. Negative values denote CH_4 uptake. Measurement periods: June 21 – August 24, 2019 (DOY 172–236) and May 30 – August 31, 2021 (DOY 150–243).

CH_4 flux, mean ± standard deviation *(mg CH_4 m^{-2} h^{-1})*				Median	Min	Max	Lower CI	Upper CI	n	obs
**May-August**										
Lichen	-0.020	±	0.016	-0.016	-0.116	0.014	-0.020	-0.020	6	14939
Shrub	-0.024	±	0.027	-0.015	-0.215	0.021	-0.024	-0.023	6	15173
Tussock	0.003	±	0.052	-0.003	-0.185	0.780	0.002	0.004	6	14736
**May**										
Lichen	-0.004	±	0.004	-0.003	-0.014	0.006	-0.005	-0.003	6	117
Shrub	-0.001	±	0.003	-0.001	-0.008	0.005	-0.002	-0.001	6	134
Tussock	0.000	±	0.006	0.000	-0.011	0.020	-0.001	0.001	6	138
**June**										
Lichen	-0.011	±	0.011	-0.009	-0.094	0.008	-0.012	-0.011	6	4432
Shrub	-0.012	±	0.019	-0.006	-0.122	0.021	-0.012	-0.011	6	4516
Tussock	-0.002	±	0.021	-0.001	-0.169	0.090	-0.002	-0.001	6	4633
**July**										
Lichen	-0.021	±	0.015	-0.018	-0.104	0.010	-0.021	-0.020	6	4178
Shrub	-0.024	±	0.021	-0.019	-0.179	0.012	-0.024	-0.023	6	4251
Tussock	-0.010	±	0.020	-0.006	-0.185	0.078	-0.011	-0.010	6	4167
**August**										
Lichen	-0.026	±	0.017	-0.023	-0.116	0.014	-0.027	-0.026	6	6212
Shrub	-0.033	±	0.031	-0.024	-0.215	0.010	-0.033	-0.032	6	6272
Tussock	0.017	±	0.077	-0.004	-0.109	0.780	0.015	0.019	6	5798

Supplementary Table 5: Climate data for Inuvik. Air temperature and precipitation measured at Inuvik (68°18’15.000" N, 133°28’58.000" W) during 2019 and 2021, as well as the long-term (1981-2010) climate mean. Data source: Environment and Climate Change Canada. *data gaps in precipitation data for year 2021: 2 days missing in July 2021, and 4 days missing in September 2021.

	Jan	Feb	Mar	Apr	May	Jun	Jul	Aug	Sept	Oct	Nov	Dec	Annual	Jun-Aug
						Air temperature, mean [°C]								
**1981-2010**	-26.6	-25.5	-22.3	-11.8	0.4	11.6	14.1	11.0	3.9	-7.6	-21.1	-24.1	-8.2	12.2
**2019**	-22.0	-16.0	-8.0	-9.0	3.6	10.3	14.6	7.8	7.2	-3.2	-14.5	-26.4	-4.6	10.9
**2021**	-22.9	-29.5	-21.6	-10.1	1.6	13.1	16.3	10.6	5.3	-3.2	-16.6	-23.7	-6.7	13.3
						Precipitation, sum [mm]								
**1981-2010**	12.5	13.1	11.9	9.8	17.3	17.3	35.0	39.4	29.3	24.4	16.0	14.8	240.6	91.7
**2019**	12.8	10.9	4.3	3.5	4.8	9.5	19.7	63.6	32.1	16.6	13.3	9.9	201.0	92.8
**2021**	2.8	1.2	12.4	6.1	3.6	12.4	4.3*	25.2	29.9*	12.2	15.9	17.6	143.6*	41.9*

Supplementary Table 6: Diel peaks in methane uptake at Trail Valley Creek. Differences in magnitude of diel maximum and minimum rates in CH_4 uptake measured with automated chambers at Trail Valley Creek in years 2019 and 2021. Table shows mean flux values (mg CH_4 m^{-2} h^{-1}) with standard deviation (s.d.), number of replicate collars per vegetation type (n) and number of observations (obs), as well as the time period used for calculation of means during which peaks occurred. Negative values denote CH_4 uptake, and percent differences between nighttime and daytime peaks were calculated only for vegetation types and periods with net CH_4 uptake.

Month, vegetation type	**Period of maximum CH_4 uptake**					**Period of minimum CH_4 uptake**						% nighttime peak of daytime peak in CH_4 uptake
	Time period	mean		s.d.	obs	Time period	mean		s.d.	obs	n	
**June**												
Lichen	15:00-16:00	-0.016	±	0.014	388	4:00-7:00	-0.008	±	0.008	677	6	49.6
Shrub	15:00-16:00	-0.020	±	0.027	396	4:00-7:00	-0.004	±	0.006	693	6	20.2
Tussock	15:00-16:00	-0.007	±	0.026	407	4:00-7:00	*+0.004*	±	0.014	708	6	n. d.
**July**												
Lichen	16:00	-0.026	±	0.017	179	7:00-8:00	-0.017	±	0.012	334	6	64.6
Shrub	16:00	-0.028	±	0.020	186	7:00-8:00	-0.019	±	0.019	331	6	70.1
Tussock	16:00	-0.019	±	0.028	176	7:00-8:00	-0.004	±	0.013	327	6	21.1
**August**												
Lichen	22:00-4:00	-0.028	±	0.018	1696	11:00	-0.023	±	0.015	253	6	120.9
Shrub	22:00-4:00	-0.038	±	0.036	1666	11:00	-0.025	±	0.024	264	6	150.0
Tussock	22:00-4:00	*+0.018*	±	0.086	1558	11:00	*+0.016*	±	0.070	253	6	111.2

Supplementary Table 7: Over- and underestimation of daily methane fluxes at Trail Valley Creek. Cumulative, daily CH_4 fluxes (based on hourly measurements) obtained with automated chambers at Trail Valley Creek in years 2019 and 2021 over 24h-periods, as well as measured only during daytime when manual chamber measurements are typically conducted (10–18h). Values for cumulative CH_4 fluxes show mean ± standard deviation. Daytime fluxes were extrapolated to daily sums, which are compared with measured daily sums. Overestimation and underestimation of daily sums were calculated as % over- or underestimation if only daytime fluxes were used to obtain daily sums.

**Month, vegetation type**	**Cumulative CH_4 flux (0–24h)**				**Daytime flux (10–18h)**		**Overestimation (+) and underestimation (-)**
	Mean daily sum, mg CH_4 m^{-2} d^{-1}			obs	Mean, mg CH_4 m^{-2} h^{-1}	Extrapolated daily sum, mg CH_4 m^{-2} d^{-1}	%
**June**							
Lichen	-0.26	±	0.20	192	-0.015	-0.35	25.3
Shrub	-0.28	±	0.28	192	-0.018	-0.44	36.6
Tussock	-0.04	±	0.42	198	-0.008	-0.18	80.4
**July**							
Lichen	-0.51	±	0.27	187	-0.024	-0.58	13.2
Shrub	-0.59	±	0.39	189	-0.026	-0.62	4.4
Tussock	-0.26	±	0.32	187	-0.015	-0.37	28.2
**August**							
Lichen	-0.64	±	0.35	274	-0.025	-0.60	-6.0
Shrub	-0.80	±	0.60	272	-0.028	-0.67	-19.1
Tussock	*+0.43*	±	1.86	247	*+0.014*	+0.35	-24.1

Supplementary Table 8: Overview table of Random Forest models. Models are based on automated chamber data and the table provides a summary of model parameters and performance.

Model		Vegetation type	Fluxes included	Years	Predictor variables included in the random forest	Number of observations	Model performance		Model parameters	
							% Variance explained	Mean of squared residuals	number of variables tried at each split	Number of trees
1	A	All	Flux < 0	2019, 2021	Plot-level, site-level	36782	47.57	2.44×10^{-4}	6	500
1	B	Lichen	All	2019, 2021	Plot-level, site-level	14244	70.02	7.73×10^{-5}	6	500
1	C	Shrub	All	2019, 2021	Plot-level, site-level	14495	48.46	3.76×10^{-4}	3	500
1	D	Tussock	All	2019, 2021	Plot-level, site-level	14038	68.48	6.29×10^{-4}	6	500
2	A	All	Flux < 0	2021	Plot-level, microsite-level, site-level	20531	56.08	2.64×10^{-4}	13	500
2	B	Lichen	All	2021	Plot-level, microsite-level, site-level	8287	76.03	7.11×10^{-5}	18	500
2	C	Shrub	All	2021	Plot-level, microsite-level, site-level	8422	49.14	5.03×10^{-4}	13	500
2	D	Tussock	All	2021	Plot-level, microsite-level, site-level	7769	59.69	2.96×10^{-4}	18	500

Supplementary Table 9: Fluxes and environmental conditions measured at Trail Valley Creek and other sites across the Arctic. Methane (CH_4) fluxes measured during summer with manual, opaque chambers, soil gas concentrations, site conditions, soil properties, and soil process rates at the intensively studied sites Trail Valley Creek (polygon rim and upland tundra) and Finnish Lapland (upland forest and palsa). Note that surface permafrost was absent at upland forest (Finnish Lapland), and process rates were not measured for Tussock as indicated by n.d. = not determined. Variables: WFPS = water-filled pore space, LOI = loss on ignition, C content = organic carbon content, N content = total nitrogen content, C:N = carbon to nitrogen ratio, DOC = dissolved organic carbon, DON = dissolved organic nitrogen. Negative values for fluxes denote net uptake and net processing rates indicate immobilization in the microbial biomass.

	Trail Valley Creek				Finnish Lapland			
	Polygon rim	Upland tundra			Upland forest		Palsa	
	Lichen	Lichen	Shrub	Tussock	Lichen	Shrub	Lichen	Shrub
	mean ± standard deviation (median)							
CH_4 flux *[mg CH_4 m^{-2} h^{-1}]*	-0.047 ± 0.032 (-0.035)	-0.033 ± 0.026 (-0.020)	-0.023 ± 0.013 (-0.020)	0.123 ± 0.159 (0.097)	-0.103 ± 0.078 (-0.090)	-0.077 ± 0.071 (-0.067)	-0.128 ± 0.043 (-0.103)	-0.105 ± 0.043 (-0.096)
CH_4 soil gas (10 cm) *[ppm]*	1.88 ± 0.06 (1.86)	1.71 ± 0.15 (1.77)	1.89 ± 0.10 (1.90)	2.04 ± 0.24 (1.96)	1.68 ± 0.20 (1.74)	1.72 ± 0.21 (1.65)	1.57 ± 0.12 (1.51)	1.10 ± 0.44 (0.87)
CH_4 soil gas (20 cm) *[ppm]*	1.47 ± 0.17 (1.42)	2.17 ± 1.29 (1.73)	1.83 ± 0.10 (1.86)	2.07 ± 0.29 (2.15)	1.31 ± 0.44 (1.18)	1.86 ± 0.24 (1.77)	1.02 ± 0.17 (1.05)	0.82 ± 0.34 (0.65)

CO_2 soil gas (10 cm)	486 ± 31	960 ± 317	649 ± 72	739 ± 209	1261 ± 521	711 ± 162	689 ± 87	621 ± 78
[ppm]	(481)	(1151)	(617)	(686)	(1167)	(743)	(697)	(647)
CO_2 soil gas (20 cm)	821 ± 188	1430 ± 453	1002 ± 349	1690 ± 1245	2638 ± 895	1543 ± 1093	899 ± 84	793 ± 134
[ppm]	(928)	(1473)	(989)	(950)	(2877)	(999)	(910)	(776)
N_2O soil gas (10 cm)	0.336 ± 0.001	0.338 ± 0.004	0.337 ± 0.001	0.337 ± 0.001	0.335 ± 0.003	0.336 ± 0.002	0.355 ± 0.009	0.352 ± 0.027
[ppm]	(0.336)	(0.339)	(0.337)	(0.336)	(0.335)	(0.336)	(0.356)	(0.342)
N_2O soil gas (20 cm)	0.339 ± 0.002	0.342 ± 0.004	0.341 ± 0.007	0.337 ± 0.001	0.333 ± 0.004	0.338 ± 0.006	0.397 ± 0.042	0.377 ± 0.067
[ppm]	(0.339)	(0.342)	(0.338)	(0.337)	(0.335)	(0.338)	(0.384)	(0.349)
Soil temperature	9.3 ± 1.1	9.9 ± 1.8	12.3 ± 2.3	10.6 ± 2.0	13.0 ± 0.8	12.4 ± 0.9	13.7 ± 1.1	12.9 ± 1.2
[°C]	(8.9)	(9.9)	(13.4)	(11.5)	(13.2)	(12.4)	(13.6)	(12.5)
Air temperature	11.9 ± 0.2	15.7 ± 0.2	15.7 ± 0.2	15.7 ± 0.2	7.3 ± 0.6	7.3 ± 0.6	11.0 ± 0.3	11.0 ± 0.3
[°C]	(11.8)	(15.6)	(15.8)	(15.7)	(7.0)	(7.0)	(10.8)	(11.1)
WFPS	40 ± 4	33 ± 6	30 ± 4	36 ± 5	34 ± 14	23 ± 5	38 ± 7	32 ± 7
[%]	(40)	(37)	(28)	(38)	(27)	(22)	(42)	(34)
Thaw depth	32 ± 2	61 ± 11	52 ± 12	47 ± 6	>100 ± n.d.	>100 ± n.d.	54 ± 14	48 ± 6
[cm]	(31)	(61)	(52)	(45)	(>100)	(>100)	(58)	(46)
pH	5.0 ± 0.7	7.3 ± 0.1	6.7 ± 0.5	7.0 ± 0.2	4.8 ± 0.3	4.6 ± 0.2	3.8 ± 0.3	4.0 ± 0.1
[--]	(4.8)	(7.3)	(6.8)	(6.9)	(4.7)	(4.6)	(3.8)	(4.0)
bulk density	0.09 ± 0.00	0.55 ± 0.33	0.09 ± 0.00	n.d. ± n.d.	0.54 ± 0.23	0.44 ± 0.13	0.13 ± 0.06	0.08 ± 0.02
[g cm^{-3}]	(0.09)	(0.61)	(0.09)	(n.d.)	(0.47)	(0.41)	(0.14)	(0.09)
Organic layer depth	>50 ± n.d.	5.63 ± 2.29	16.50 ± 2.12	0-40 ± n.d.	1.80 ± 0.45	2.20 ± 0.45	>100 ± n.d.	>100 ± n.d.
[cm]	(>50)	(6.00)	(16.50)	(0-40)	(2.00)	(2.00)	(>100)	(>100)
LOI	93.7 ± 0.6	44.8 ± 24.8	85.9 ± 12.7	32.7 ± 35.1	8.8 ± 3.5	8.6 ± 2.0	96.2 ± 1.9	98.5 ± 0.4
[%]	(93.8)	(41.7)	(90.4)	(20.3)	(7.8)	(7.9)	(95.5)	(98.3)
C content	47.3 ± 0.2	24.3 ± 12.1	43.2 ± 6.8	18.6 ± 19.2	4.2 ± 2.2	3.9 ± 0.9	49.4 ± 2.3	48.1 ± 1.8
[%]	(47.2)	(21.4)	(45.5)	(13.4)	(3.8)	(3.8)	(48.4)	(47.7)
N content	1.2 ± 0.1	1.2 ± 0.4	1.2 ± 0.5	0.9 ± 0.9	0.2 ± 0.1	0.2 ± 0.0	1.5 ± 0.6	1.0 ± 0.5
[%]	(1.2)	(1.1)	(1.3)	(0.6)	(0.2)	(0.1)	(1.7)	(1.1)
C:N ratio	39 ± 2	22 ± 2	44 ± 29	20 ± 2	24 ± 4	24 ± 1	39 ± 20	61 ± 31
[--]	(38)	(22)	(29)	(21)	(24)	(24)	(31)	(44)
d^{13}C	-26.2 ± 0.0	-23.8 ± 3.0	-27.4 ± 0.3	-26.5 ± 0.8	-27.0 ± 0.5	-27.1 ± 0.3	-27.3 ± 0.3	-28.1 ± 0.8
[--]	(-26.2)	(-23.8)	(-27.5)	(-26.6)	(-27.1)	(-27.0)	(-27.3)	(-28.0)
d^{15}N	-0.37 ± 0.44	-0.44 ± 1.64	0.19 ± 1.86	1.24 ± 1.12	1.25 ± 0.56	2.08 ± 1.12	2.99 ± 1.81	2.08 ± 1.08
[--]	(-0.45)	(-1.42)	(0.09)	(1.23)	(1.29)	(2.19)	(3.19)	(2.61)
DOC	744 ± 427	519 ± 639	1259 ± 739	456 ± 277	121 ± 51	177 ± 80	778 ± 222	1796 ± 759

[µg C g DW^{-1}]	(951)	(333)	(1048)	(439)	(88)	(171)	(790)	(1725)
DON	0.00 ± 0.00	12.47 ± 22.52	17.43 ± 28.45	5.78 ± 3.05	82.91 ± 42.37	123.29 ± 72.24	735.67 ± 200.25	1485.83 ± 746.43
[µg N g DW^{-1}]	(0.00)	(2.71)	(0.00)	(5.90)	(67.96)	(120.59)	(767.87)	(1553.97)
net ammonification	-0.52 ± 0.83	-0.17 ± 0.13	-0.51 ± 0.58	n.d. ± n.d.	0.00 ± 0.01	0.00 ± 0.00	0.21 ± 0.26	0.01 ± 0.02
[µg NH_4^+-N g DW^{-1} d^{-1}]	(-0.11)	(-0.12)	(-0.34)	(n.d.)	(0.00)	(0.00)	(0.09)	(0.00)
net nitrification	0.00 ± 0.00	0.00 ± 0.00	0.00 ± 0.01	n.d. ± n.d.	0.00 ± 0.00	0.00 ± 0.00	0.00 ± 0.04	0.00 ± 0.00
[µg NO_3^--N g DW^{-1} d^{-1}]	(0.00)	(0.00)	(0.00)	(n.d.)	(0.00)	(0.00)	(-0.01)	(0.00)
net N mineralization	-0.52 ± 0.83	-0.17 ± 0.13	-0.51 ± 0.58	n.d. ± n.d.	0.00 ± 0.01	0.00 ± 0.00	0.21 ± 0.28	0.01 ± 0.02
[µg N g DW^{-1} d^{-1}]	(-0.11)	(-0.12)	(-0.34)	(n.d.)	(0.00)	(0.00)	(0.06)	(0.00)
nitrite turnover	0.00 ± 0.01	-0.05 ± 0.02	-0.01 ± 0.02	n.d. ± n.d.	0.00 ± 0.00	0.00 ± 0.00	-0.01 ± 0.01	-0.01 ± 0.00
[µg NO_{2-} g DW^{-1} d^{-1}]	(0.01)	(-0.06)	(-0.01)	(n.d.)	(0.00)	(0.00)	(-0.01)	(-0.01)
sulphate turnover	-0.51 ± 0.32	-0.43 ± 0.20	-0.46 ± 0.35	n.d. ± n.d.	-0.03 ± 0.06	0.10 ± 0.47	0.21 ± 0.22	0.00 ± 0.22
[µg SO_4^{2-} g DW^{-1} d^{-1}]	(-0.41)	(-0.43)	(-0.27)	(n.d.)	(-0.01)	(-0.03)	(0.16)	(0.02)
phosphate turnover	-0.53 ± 0.44	-0.08 ± 0.16	-0.78 ± 0.79	n.d. ± n.d.	-0.01 ± 0.01	0.00 ± 0.00	0.19 ± 0.31	0.40 ± 0.47
[µg PO_4^{3-} g DW^{-1} d^{-1}]	(-0.42)	(0.00)	(-0.72)	(n.d.)	(0.00)	(0.00)	(0.23)	(0.24)
chloride turnover	-0.09 ± 0.22	-0.15 ± 0.10	0.15 ± 0.42	n.d. ± n.d.	-0.12 ± 0.05	-0.31 ± 0.33	-1.16 ± 0.57	-1.54 ± 0.61
[µg Cl^- g DW^{-1} d^{-1}]	(-0.04)	(-0.13)	(-0.04)	(n.d.)	(-0.10)	(-0.32)	(-0.92)	(-1.46)
DOC turnover	0.42 ± n.d.	n.d. ± n.d.	-20.49 ± 16.71	n.d. ± n.d.	-2.36 ± 1.26	-4.10 ± 2.40	-6.87 ± 10.53	-40.15 ± 29.02
[µg C g DW^{-1} d^{-1}]	(0.42)	(n.d.)	(-20.49)	(n.d.)	(-2.44)	(-3.49)	(-7.45)	(-38.24)

Supplementary Table 10. Methane fluxes measured with manual chambers at Trail Valley Creek and other sites across the Arctic. Methane (CH_4) fluxes measured with manual, opaque chambers during summer at upland and permafrost peatland sites in the Western Canadian and European Arctic, summarized by vegetation type (Lichen vs. Shrub), site, and region. Table shows mean and median values with standard deviation (s.d.) and standard error (s.e.), adjusted p-values and number of observations (obs). A weighted mean was calculated for the Canadian sites due to the large number of observations at the primary site, Trail Valley Creek, compared to other Canadian sites. Negative values denote CH_4 uptake. Levels of significance: P-values are derived from Welch's two-sample t-test (two-tailed).

**Site**	**Vegetation type**	**obs**	**median** *mg CH_4 m^{-2} h^{-1}*	**mean**	**s.d.**	**s.e.**	**p-value**
Trail Valley Creek	Lichen	56	-0.033	-0.050	0.042	0.006	
Trail Valley Creek	Shrub	40	-0.019	-0.036	0.041	0.007	
Trail Valley Creek	*Tussock*	*40*	*0.015*	*0.049*	*0.149*	*0.023*	
Havikpak Creek	Lichen	5	-0.017	-0.032	0.044	0.020	
Havikpak Creek	Shrub	10	-0.058	-0.048	0.065	0.021	
Scotty Creek	Lichen	3	-0.049	-0.051	0.021	0.012	
Scotty Creek	Shrub	2	-0.013	-0.013	0.053	0.038	
Kilpisjärvi	Lichen	30	-0.142	-0.156	0.075	0.014	
Kilpisjärvi	Shrub	30	-0.110	-0.130	0.081	0.015	
Trail Valley Creek	Lichen, Shrub	96	-0.028	-0.044	0.042	0.004	
Havikpak Creek	Lichen, Shrub	15	-0.041	-0.043	0.058	0.015	
Scotty Creek	Lichen, Shrub	5	-0.049	-0.036	0.037	0.017	
Kilpisjärvi	Lichen, Shrub	60	-0.120	-0.143	0.079	0.010	
Canadian sites	Lichen	64	-0.032	-0.049	0.041	0.005	
Canadian sites	Shrub	52	-0.025	-0.037	0.046	0.006	0.1555
Finnish sites	Lichen	30	-0.142	-0.156	0.075	0.014	
Finnish sites	Shrub	30	-0.110	-0.130	0.081	0.015	0.1961
all sites	Lichen	94	-0.075	-0.083	0.074	0.008	
all sites	Shrub	82	-0.059	-0.071	0.075	0.008	0.2846
Canada	Lichen, Shrub	116	-0.029	-0.044	0.044	0.004	
Canada, *weighted mean*	Lichen, Shrub	3	-0.039	-0.041	0.046	0.012	
Kilpisjärvi	Lichen, Shrub	60	-0.120	-0.143	0.079	0.010	7.49×10^{-14}
All sites	Lichen, Shrub	176	-0.067	-0.077	0.074	0.006	
All sites, *weighted mean*	Lichen, Shrub	2	-0.080	-0.092	0.063	0.011	

Supplementary Table 11. **Upscaled growing season methane uptake.** First-order approximation of growing season methane (CH_4) uptake by uplands based on median and mean flux values (Supplementary Table 10), as well as uncertainty ranges, calculated as lower (5 %) and upper (95 %) confidence intervals, scaled up to the study region. Note that this approximation is for the growing season (June–August), while annual CH_4 uptake may well be larger.

Flux, mg CH_4 m^{-2} h^{-1}	Flux, mg CH_4 m^{-2} d^{-1}	Area, $\times 10^6$ km^2	Flux, g CH_4 m^{-2} growing season^{-1}	Tg CH_4 growing season^{-1}	Uncertainty range	Upland area estimate
BASED ON MEDIAN FLUX						
-0.080	-1.92	18.68	-0.177	-3.30	-0.06 to -6.96	BAWLD domain, uplands[1]
-0.080	-1.92	16.22	-0.177	-2.87	-0.05 to -6.04	This study, dry and mesic uplands[2]
-0.080	-1.92	15.37	-0.177	-2.71	-0.05 to -5.73	This study, dry uplands[2]
BASED ON MEAN FLUX						
-0.092	-2.208	18.68	-0.203	-3.79	-0.06 to -6.96	BAWLD domain, uplands[1]
-0.092	-2.208	16.22	-0.203	-3.29	-0.05 to -6.04	This study, dry and mesic uplands[2]
-0.092	-2.208	15.37	-0.203	-3.12	-0.05 to -5.73	This study, dry uplands[2]

Supplementary Table 12: Technical specifications of greenhouse gas analyzers used in this study. Table shows values of analyzer precision as specified by the manufacturers.

**Precision** (1 sigma)	**LGR Enhanced Performance**			**LGR Ultraportable**			**Picarro GasScouter**		
	CH_4 *ppb*	CO_2 *ppb*	H_2O *ppm*	CH_4 *ppb*	CO_2 *ppb*	H_2O *ppm*	CH_4 *ppb*	CO_2 *ppb*	H_2O *ppm*
1 sec.	1	300	15	< 2 ppb	< 300	< 100	-	-	-
5 sec.	-	-	-	-	-	-	3	400	100 + 5%
100 sec.	0.3	50	5	< 0.25	< 40	< 15	-	-	-
300 sec.	-	-	-	-	-	-	0.3	40	10 + 5%

Supplementary Table 13: Linear-mixed-effects models. Structure and performance of linear-mixed-effects models applied to automated chamber data.

Model	Land cover type	Years	Range of fluxes included	Outlier removal	Data transformation	% of variance explained by random effects	Conditional R^2 (total explanatory power)	Marginal R^2 (explanatory power of fixed effects)
M1	All	2019, 2021	Flux < 0	Outside of 1 and 99 percentile	$1/(\log_{10}(Flux)^2)$	30	0.47	0.24
$Flux \sim PAR_{chamber} + WFPS_{chamber} + SoilTemp_{chamber}:WFPS_{chamber} + WindSpeed + AirTemp + PPFD + rH + (1\|Collar)$								
M2	Lichen	2019, 2021	all	none	none	29	0.36	0.10
$Flux \sim SoilTemp_{chamber}:WFPS_{chamber} + WindSpeed + AirTemp + (1\|Collar)$								
M3	Shrub	2019, 2021	all	none	$1/(\log_{10}(Flux)^2)$	25	0.53	0.38
$Flux \sim PAR_{chamber} + WFPS_{chamber} + SoilTemp_{chamber}:WFPS_{chamber} + WindSpeed + (1\|Collar)$								
M4	Tussock	2019, 2021	all	none	none	11	0.20	0.09
$Flux \sim PAR_{chamber} + WFPS_{chamber} + SoilTemp_{chamber} + PPFD + (1\|Collar)$								

Supplementary Table 14: Model comparison. Comparison of Random Forest (RF) models with and without inclusion of ecosystem respiration (ER), in addition to soil moisture and surface soil temperature (shown in Fig. 4 and Extended Data Fig. 6), and tests for overfitting of RF models. Statistics for model comparison were calculated based on running the RF models one hundred times. Improved models with inclusion of ER are highlighted in bold. All differences were statistically significant at p < 0.001 using Welch's two-sample t-test (two-tailed). Potential model overfitting was assessed by comparison of root mean square error (RMSE) of RF models run on training and validation data, where a significantly higher RMSE using validation data would indicate overfitting in the RF models. s.d. = standard deviation, CI = confidence interval.

			RF model without ER			RF model with ER				
Chamber no.	Vegetation type	Month	% explained variance	s.d.	95% CI	% explained variance	s.d.	95% CI	RMSE training data	RMSE validation data
1	Lichen	June	47.7	0.40	0.08	44.3	0.45	0.09	0.0027	0.0025
2	Lichen	June	65.0	0.37	0.07	63.4	0.47	0.09	0.0088	0.0085
3	Shrub	June	63.3	0.25	0.05	**71.8**	**0.28**	**0.06**	0.0091	0.0091
4	Lichen	June	54.4	0.28	0.06	**64.6**	**0.30**	**0.06**	0.0047	0.0048
5	Lichen	June	76.1	0.22	0.04	71.6	0.28	0.06	0.0040	0.0040
6	Shrub	June	74.2	0.23	0.05	68.7	0.29	0.06	0.0138	0.0116
7	Shrub	June	58.8	0.38	0.07	**62.5**	**0.33**	**0.07**	0.0054	0.0063
8	Lichen	June	44.5	0.43	0.08	**78.8**	**0.28**	**0.06**	0.0033	0.0037
9	Shrub	June	69.5	0.28	0.06	**71.5**	**0.30**	**0.06**	0.0107	0.0114
10	Tussock	June	49.7	0.35	0.07	**51.1**	**0.40**	**0.08**	0.0028	0.0031
11	Tussock	June	63.2	0.37	0.07	**66.9**	**0.34**	**0.07**	0.0157	0.0195
12	Shrub	June	49.2	0.36	0.07	**59.2**	**0.48**	**0.09**	0.0189	0.0163
13	Tussock	June	79.9	0.17	0.03	**81.7**	**0.24**	**0.05**	0.0048	0.0049
14	Lichen	June	76.0	0.15	0.03	**76.3**	**0.19**	**0.04**	0.0057	0.0057
15	Tussock	June	51.5	0.32	0.06	**54.3**	**0.34**	**0.07**	0.0035	0.0034
16	Tussock	June	68.4	0.33	0.07	67.1	0.40	0.08	0.0155	0.0165
17	Tussock	June	73.6	0.16	0.03	**82.7**	**0.23**	**0.05**	0.0082	0.0079
18	Shrub	June	58.7	0.36	0.07	**60.7**	**0.42**	**0.08**	0.0084	0.0080
1	Lichen	July	69.7	0.20	0.04	**76.5**	**0.20**	**0.04**	0.0034	0.0032
2	Lichen	July	65.6	0.33	0.07	**66.2**	**0.37**	**0.07**	0.0116	0.0113
3	Shrub	July	34.0	0.36	0.07	**50.7**	**0.34**	**0.07**	0.0076	0.0081
4	Lichen	July	74.5	0.23	0.05	**76.5**	**0.28**	**0.06**	0.0044	0.0054
5	Lichen	July	66.5	0.20	0.04	**67.7**	**0.22**	**0.04**	0.0065	0.0076
6	Shrub	July	59.5	0.28	0.05	**62.5**	**0.37**	**0.07**	0.0098	0.0111
7	Shrub	July	58.8	0.35	0.07	**69.7**	**0.26**	**0.05**	0.0148	0.0203
8	Lichen	July	71.8	0.17	0.03	**82.4**	**0.16**	**0.03**	0.0065	0.0064
9	Shrub	July	72.9	0.18	0.03	**75.6**	**0.20**	**0.04**	0.0119	0.0116
10	Tussock	July	63.7	0.39	0.08	55.7	0.61	0.12	0.0060	0.0069
11	Tussock	July	68.7	0.22	0.04	68.5	0.25	0.05	0.0079	0.0086
12	Shrub	July	64.0	0.31	0.06	**79.8**	**0.24**	**0.05**	0.0096	0.0087
13	Tussock	July	66.3	0.26	0.05	**69.0**	**0.33**	**0.07**	0.0058	0.0049
14	Lichen	July	67.7	0.24	0.05	**70.8**	**0.25**	**0.05**	0.0072	0.0058
15	Tussock	July	55.9	0.29	0.06	**59.1**	**0.33**	**0.07**	0.0087	0.0097
16	Tussock	July	59.5	0.48	0.10	**59.7**	**0.51**	**0.10**	0.0207	0.0202
17	Tussock	July	84.7	0.22	0.04	**84.9**	**0.24**	**0.05**	0.0105	0.0089
18	Shrub	July	49.5	0.35	0.07	**52.6**	**0.36**	**0.07**	0.0152	0.0120
1	Lichen	August	37.0	0.28	0.06	**66.4**	**0.19**	**0.04**	0.0066	0.0062
2	Lichen	August	60.7	0.26	0.05	**62.4**	**0.32**	**0.06**	0.0043	0.0042
3	Shrub	August	28.6	0.24	0.05	**71.3**	**0.14**	**0.03**	0.0078	0.0080
4	Lichen	August	68.3	0.14	0.03	**80.2**	**0.13**	**0.03**	0.0060	0.0062
5	Lichen	August	48.1	0.38	0.07	**61.4**	**0.28**	**0.06**	0.0071	0.0062
6	Shrub	August	36.8	0.23	0.04	**77.3**	**0.12**	**0.02**	0.0075	0.0075
7	Shrub	August	38.3	0.33	0.07	**58.9**	**0.31**	**0.06**	0.0310	0.0294
8	Lichen	August	48.1	0.25	0.05	**71.2**	**0.20**	**0.04**	0.0103	0.0112
9	Shrub	August	32.6	0.29	0.06	**60.0**	**0.28**	**0.06**	0.0154	0.0145
10	Tussock	August	93.6	0.10	0.02	**95.9**	**0.13**	**0.02**	0.0363	0.0394
11	Tussock	August	85.3	0.13	0.03	**88.2**	**0.17**	**0.03**	0.0152	0.0141
12	Shrub	August	29.9	0.29	0.06	**80.2**	**0.16**	**0.03**	0.0116	0.0117
13	Tussock	August	54.8	0.42	0.08	**56.7**	**0.56**	**0.11**	0.0250	0.0279
14	Lichen	August	62.4	0.24	0.05	**64.1**	**0.22**	**0.04**	0.0052	0.0052
15	Tussock	August	40.5	0.28	0.06	**57.6**	**0.30**	**0.06**	0.0065	0.0072
16	Tussock	August	81.5	0.14	0.03	**83.9**	**0.20**	**0.04**	0.0286	0.0279
17	Tussock	August	69.9	0.18	0.04	**77.5**	**0.23**	**0.05**	0.0082	0.0080
18	Shrub	August	43.6	0.25	0.05	**72.7**	**0.14**	**0.03**	0.0121	0.0117

Supplementary Methods

Site description

Trail Valley Creek is situated just north of the tree line east of the Mackenzie Delta and is underlain by continuous ice-rich permafrost. The long-term (1981–2010) mean annual air temperature (MAAT) determined for Inuvik is -8.2 °C and mean annual precipitation (MAP) amounts to 241 mm of which >50 % fall as snow[3]. The site is typically snow-covered for eight months of the year (October–May) during which mean monthly air temperature remains below freezing point **(Supplementary Table 5).** Most of the landscape (59 %) at Trail Valley Creek is classified as low or erect dwarf-shrub tundra (classes S1 and S2)[4,5], followed by slightly wetter, mesic tundra (classes G4: tussock-sedge, dwarf-shrub, moss tundra)[4,5] covering 37 %, while the remainder is covered by scattered tree patches and waterbodies[6]. In the well-drained shrub tundra classes (S1, S2), the dominant vegetation types are deciduous shrubs such as dwarf birch (*Betula nana* subsp. *glandulosa*), labrador tea (*Ledum palustre* L.), herbaceous species such as mountain cranberry (*Vaccinium vitis-idaea* L.), bearberry (*Arctostaphylos uva-ursi* L.), bilberry (*Vaccinium uliginosum* L.), crowberry (*Empetrum nigrum* L.), forbs, graminoids, bryophytes (e.g., *Aulacomnium* sp.), and a well-developed lichen layer (*Alectoria ochroleuca*, *Cladonia* sp., *Gowardia* sp., *Flavocetraria* sp.). Taller shrubs (> 40 cm) such as willow (*Salix* sp.) and green alder (*Alnus viridis*) occur pre-dominantly in depressions and riparian areas, although short willow shrubs (< 40 cm) are interspersed with other deciduous shrubs in the drier areas as well. In poorly drained areas (class G4), tussock-forming sedges (*Eriophorum* sp., *Carex* sp.) dominate, but bryophytes and lichen cover the inter-tussock spaces. In landscape depressions, polygonal tundra has developed, characterized by a thick (> 50 cm) mat of peat-forming mosses (*Sphagnum* sp.) which fill the wet polygon troughs and form poorly decomposed organic layer in the polygon centers and rims. The surface of the predominantly high centered polygons is dominated by lichen in polygon centers and rims interspersed with occasional cloudberry (*Rubus chamaemorus* L.), Labrador tea, and cranberry. Apart from the polygonal tundra areas, the Trail Valley Creek region is underlain by ice-rich, morainal deposits and consists of mineral soils several metres thick in most places, overlain by a thin organic layer of typically <10 cm under lichen and around tussocks, and <25 cm under shrubs (**Supplementary Figure 9**, **Supplementary Table 9**). The thickness of the average active layer at the site is 40–70 cm[ref 7] although thaw can be deeper in locations with a thin organic layer. More details on the site can be found elsewhere[6,8,9].

Havikpak Creek is located close to Inuvik Airport on continuous permafrost, ca. 50 km south of Trail Valley Creek. Havikpak Creek is a boreal upland forest near (ca. 30–40 km) the arctic tree line. Vegetation at the site is dominated by black spruce (*Picea mariana*)[10], and understory vegetation at the location where manual chamber measurements were conducted compares to well-drained lichen-shrub tundra at Trail Valley Creek (dwarf birch, Labrador tea, mountain cranberry, bearberry, bilberry, crowberry, cloudberry, bryophytes and lichen). The organic layer at Havikpak Creek is thicker than at Trail Valley Creek and amounts to 10–60 cm overlaying mineral soil[10], while no information is available on active layer thickness.

Scotty Creek is a thawing peat landscape in the boreal forest biome, located in the lower Liard River valley in the discontinuous to sporadic permafrost zone, ca. 850 km south of Trail Valley Creek. The long-term (1981–2010) MAAT at nearby Fort Simpson is -2.8 °C, and MAP is 388 mm, 60 % of which falls as rain[3,11]. The site is situated at the southern extent of permafrost distribution, overlain by 2–8 m thick peat deposits, and the landscape consists of raised, forested permafrost peat plateaus, lakes, and permafrost-free wetlands that include ombrotrophic bogs as well as nutrient-rich fens[11–13]. Manual chamber measurements at Scotty Creek were carried out on a peat plateau, elevated above the surrounding fen and bog landscapes through permafrost aggradation, and where permafrost is preserved by the insulating properties of peat[12]. Like Havikpak Creek, the peat plateau is forested by black spruce (*picea mariana*) and ground vegetation consists of labrador tea, mountain cranberry, bryophytes (e.g., *Hylocomium splendens*, *Pleurozium schreberi*, *Dicranum* sp.) and lichen species[13]. The average active layer on the intact peat plateaus is ca. 60 cm and >160 cm on degrading, actively thawing parts of the peat plateaus[12].

Kilpisjärvi, located in Finnish Lapland in proximity to the Swedish and Norwegian border, is situated in the sporadic permafrost zone marking the southern extent of permafrost. The long-term (1981–2010) MAAT at Kilpisjärvi station is -1.9 °C, and MAP is 487 mm[ref 14]. Permafrost has mostly disappeared in Kilpisjärvi, but, similar to Scotty Creek, is preserved in elevated peat complexes called palsas[15,16], covered by dwarf shrubs and herbaceous species (*Betula nana* L., *Rubus chamaemorus* L., *Empetrum nigrum* L*, vaccinium vitis-idaea* L.), and in some wetter areas bryophytes (*Dicranum* sp., *Polytrichum* sp., *Pleurozium* sp.)[17]. Palsas contain the largest soil C pools in the Kilpisjärvi region but cover relatively small areas (<2 %)[18,19]. The most dominant land cover types in the region are mountain birch forest (*Betula pubescens var. pumila*) and upland tundra located on the surrounding mountains ('fells'). Soils in these upland ecosystems are well-drained, rocky, and have a thin organic layer (<5 cm) and small soil C stocks[20]. Ground vegetation at these upland sites consists primarily of dwarf–shrub heaths, with *Empetrum nigrum* L., *Betula nana* L., *Vaccinium myrtillus* L., *Vaccinium vitis-idaea* L. and *Phyllodoce caeruleae* (L.) Bab. as the dominant species. Patches of lichen (pre-dominantly *Cladonia* sp.) occur at all these landforms but are subjected to grazing by reindeer, often resulting in patches of barren ground.

Automated chamber flux measurements – system maintenance, flux calculation and quality control

The chamber system was powered by an on-site hybrid energy system providing AC power between June 21 – August 24, 2019 (DOY 172–236) and May 30 – August 31, 2021 (DOY 150–243). Site access was not possible in 2020 due to travel restrictions in response to the COVID-19 pandemic. The resulting lack of required maintenance led to power system failure for a four-week period in July 2021, causing a gap in automated chamber data collection. However, during most of the measurement periods in 2019 and 2021, trained personnel were on-site for daily routine visual inspections and maintenance tasks to ensure high-quality data collection by the automated chamber system. During periods when site visits were not possible, performance of the chamber system was monitored via remote access to the autochamber PC. At the beginning of each measurement season chamber inlet and outlet tubes were flushed with dry compressed gas to remove water due to condensation in the tubes. During operation of the chamber system, fluctuations in pressure, caused by clogged 7-µm filters or water in the inlet tube, were resolved by cleaning filters in an ultrasonic bath, and cleaning water traps with pressurized air. In the rare event that a pressure build-up occurred due to blocked tubing, a built-in safety mechanism switched the airflow from the affected chamber to an additional outlet line, to release pressure in the system and avoid damaging the pump. In addition to power outages and variations in pressure, possible issues included broken fans, or leakage due to vegetation caught in between chamber lid and collar. In spring 2021, low air temperature and snowfall events resulted in limited lid movement and poor seal of the chambers for 2–3 days. Gas analyzer, pump, and control system, as well as lids were removed over the autumn and winter months to reduce the artificial formation of snow drifts over the chamber site and to prevent damage caused by strong winds and harsh environmental conditions. The sensitive components such as the gas analyzer, pump and site PC were placed in a heated storage over winter.

Data processing was performed with various routines developed in-house in the MATLAB computing environment, version R2020b (The MathWorks Inc., Natick, MA, USA). Selection of the slope initiation for flux calculation was automated according to following criteria: i) data immediately after chamber closure (usually 30–55 s; **Supplementary Figure 10**) were skipped to account for the time it takes for the air sample to travel through the 38-m-long tube and flush it, ii) a deadband (usually 40 s) encompassing the timing of lid movement to close the chamber, and iii) a window of 20 data points within the deadband that were individually tested as a starting point for the slope selection of a best fit. Fluxes were calculated as follows:

$$F = \frac{\mathrm{V}\times\mathrm{P}\times\left(1-\frac{\chi_\mathrm{v}}{1000}\right)}{\mathrm{A}\times T_{abs}\times R}\times\frac{dc}{dt} \quad (1)$$

where F is the flux of CH_4 or CO_2 [µmol m^{-2} s^{-1}], V is the volume of the chamber [m^3], P is the initial atmospheric pressure [Pa], χ_v is the initial water vapour mole fraction during the measurement [mmol mol^{-1}], A is the collar area [m^{-2}], T_{abs} is the initial temperature during the measurement [K], R is the universal gas constant [8.314 Pa m^3 mol^{-1}

K^{-1}], and $\frac{dc}{dt}$ is the rate of change in dry CH_4 or CO_2 mole fraction (or mixing ratio; i.e., slope) [µmol mol^{-1} s^{-1}]. Fluxes were converted to mass units and are reported here as mg CH_4 m^{-2} h^{-1} or mg CH_4 m^{-2} d^{-1}, where negative fluxes denote net uptake and positive fluxes represent emissions to the atmosphere. Fluxes of CO_2 were preferentially calculated using exponential fits. Poor (i.e., low root mean square error; RMSE) exponential fits that did not meet quality criteria were replaced, if available, with 'good' linear fits. In total, 84 % of CO_2 fluxes were calculated using exponential fits, and 16 % using linear fits. As the chamber CH_4 concentration increase or decrease was mostly small compared to that of CO_2, fluxes of CH_4 were preferentially calculated using linear fits. However, exponential fits were selected if they yielded a better fit for fluxes above a certain threshold ($\frac{dc}{dt}$ of linear fit >0.0001 or <-0.0001 µmol mol^{-1} s^{-1}). A total of 93 % of CH_4 fluxes were calculated using linear fits, and 7 % using exponential fits.

The following quality criteria were applied to retain high-quality fluxes for further analyses: total RMSE limit = 1 (CO_2) and 0.005 (CH_4), total flux limit = -10 to +20 (CO_2) and -0.02 to +0.02 (CH_4), BinLimits = 0 to 1 (CO_2) and 0 to 0.001 (CH_4), BinWidth = 0.01 (CO_2) and 0.00001 (CH_4); units all in µmol m^{-2} s^{-1}. Individual RMSE thresholds for inclusion of fluxes were calculated for each chamber and each season of measurement using mean RMSE + 2*standard deviation of the RMSE. RMSE thresholds for all chambers and years were between 0.0006–0.0008 for CH_4, and between 0.1547–0.6681 for CO_2. Diagnostic plots were created for each chamber and the entire measurement periods in 2019 and 2021. When diagnostics plots revealed irregularities in the data that were not filtered out with the automated data cleaning procedure, slopes were inspected visually and compared with field notes for periods of maintenance of the chamber system, broken fans, renewed system start-up after power outages, negative CO_2 fluxes (ER) measured with dark chambers (for those periods, CH_4 fluxes were also removed when they indicated issues with flow rate or chamber closure), or elevated starting concentrations of CO_2 and CH_4 above ambient (CH_4 >2.5 ppm, CO_2 >600 ppm, H_2O >20 000 ppm). For those cases, if not detected by the automated procedure, additional manual filtering was applied. For CH_4 fluxes, all cases when exponential fits were selected by the MATLAB algorithm were inspected visually. Similarly, when the MATLAB algorithm failed to select either linear or exponential fit, fluxes were also inspected visually. In most of these cases, the concentration in the chamber increased exponentially but remained below the set threshold for applying exponential fits. In those cases, exponential fits were selected manually.

Manual chamber flux measurements – chamber design and flux processing

Methane fluxes with manual chambers at the Canadian sites were measured using transparent plexiglas chambers (cylindrical, headspace volume: 17 L, base area: 0.054 m^2) with an opaque cover made of reflective bubble wrap for simulated dark conditions. The plexiglas chamber was equipped with a fan and equilibrium pressure vent, and temperature in and outside the chamber was monitored using a HOBO Microstation logger (E348-H21-USB, Onset, Bourne, MA, USA) connected to two temperature sensors (E348-S-TMB-M002 Smart Temp Sensor 12-bit, Onset). At the Finnish sites, we used an opaque, aluminum chamber (conical with flat top, headspace volume: 29 L, base area: 0.096 m^2), equipped with a fan and equilibrium pressure outlet, as well as a HOBO temperature sensor (UA-001-08 Pendant, Onset).

Gas concentrations were recorded at 1 s intervals over an enclosure time of 5 min, and fluxes were calculated based on linear and non-linear model fits using the *chamberflux* script[21] in MATLAB version R2020b. The first 10 s after chamber closure were discarded for the flux calculation, and for calculation of CH_4 fluxes a time interval of 200 sec was used for slope calculation. Each slope was inspected visually along with chamber headspace temperature recorded during the flux measurement. If the temperature increase or decrease was larger than 1.5 °C, the measurement interval used for flux calculation was adjusted manually, to ensure stable temperature conditions during the measurement. If the RMSE exceeded the analyzer precision, the curve fit was visually reinspected. The best model fit was selected based on RMSE, adjusted R^2, and the Akaike Information Criterion. To further filter fluxes we used the following quality criteria: an adjusted R^2 > 0.85 and, based on visual inspections of scatter plots we set an RMSE cut-off value of 0.004 ppm for CH_4. Small fluxes (> -0.1 and < 0.1 mg CH_4 m^{-2} h^{-1}) were accepted

regardless of their adjusted R^2 value, to avoid overestimation of fluxes. Based on these quality criteria, the linear fit was selected for 68 % of CH_4 fluxes, whereas the remaining fluxes used exponential fits, and filtering discarded 1.1 % of CH_4 fluxes resulting in a final dataset of 176 individual, manual chamber fluxes (**Supplementary Table 10**).

Flux upscaling

To estimate atmospheric CH_4 uptake across the northern circumpolar permafrost region, we used our manual chamber measurements conducted in uplands and other well-drained landforms in the tundra and boreal biomes. The area of uplands vs. wetlands was calculated using the ESA CCI land cover product at 300-m resolution[2]. For a conservative estimate, we additionally distinguished between dry upland areas such as lichen- and dwarf shrub-dominated mineral upland tundra, upland forests or dry permafrost peatlands that can be considered growing season CH_4 sinks, as well as wetter, mesic tundra areas such as tussock tundra that may act as small CH_4 source or switch between being a net sink or source of CH_4. The land cover product was first cropped to contain only areas with permafrost[22] within the tundra and boreal biomes[23]. We used the following classes to represent dry uplands: tree cover (classes 50–90), mosaic tree, shrub, or herbaceous cover (classes 100 and 110), shrubland (classes 120–122), sparse vegetation (classes 150–153), bare areas (classes 200–202). Grasslands (class 130) were used to represent mesic uplands from the ESA CCI land cover product[2]. Wetlands were defined based on the flooded vegetation classes (160–180). Atmospheric CH_4 uptake was estimated for the growing season (90 days). Our upscaled values should be considered as a first-order approximation and are presented as mean and median with 95 % confidence intervals as uncertainty estimates.

Soil gas, soil sampling and analyses

Soil pore gas samples for determining concentrations of CH_4, CO_2, and N_2O were collected from 2-cm, 5-cm, 10-cm, 20-cm depths as well as in ambient air using a stainless-steel tube equipped with three-way-valve, following the procedure described by Marushchak et al.[24] Soil gas samples at Trail Valley Creek were taken bi-weekly at the time of manual chamber flux measurements. At all other sites, soil gases were taken once on the same day as manual chamber flux measurements, except at Havikpak Creek, where soil gases were collected on three occasions (see **Supplementary Table 2** for details). Analysis took place on a gas chromatograph (Agilent 7890B, Agilent Technologies, Santa Clara, CA, USA) with autosampler (Gilson Inc., Middleton, WI, USA), flame ionization detector (FID) for methane (CH_4), a thermal conductivity detector (TCD) for CO_2, and an electron capture detector (ECD) for N_2O. Gas concentrations were determined using two certified standards, with a CH_4 concentration of 2.02 and 15 ppm, a CO_2 concentration of 398 and 3990 ppm, and a N_2O concentration of 0.84 and 5000 ppm. As N_2O concentrations were near ambient in all locations and thus below the lowest standard, we used ambient air samples collected at the same time as soil gas samples to correct N_2O concentrations, taking atmospheric observations collected by NOAA Global Monitoring Laboratory[25] during the growing season for the Canadian sites (2018: 331.20 ppb, 2019: 332.07 ppb, 2021: 334.66 ppb; measured at Barrow, Alaska) and the Finnish sites (2021: 334.65 ppb; measured at Pallas, Finland).

Soil samples were taken from the top 0–10 cm. Soils were homogenized, and visible roots removed within two days from sampling. Soil sampling at Trail Valley Creek took place in the second half of June, 2021, and in Kilpisjärvi at the end of August, 2021. Soils collected in Finnish Lapland were stored at 4°C until further processing, whereas soils collected at Trail Valley Creek were frozen during transport and until further analyses.

Soil pH was determined in a soil slurry with a 1:2 volume ratio of deionized water and fresh soil. Soil bulk density was determined by drying soil samples of known volume to a constant weight. At the Finnish sites, bulk density was determined for each manual chamber measurement location (n = 5) for the top 0–5 cm of soil (dried at 65°C). At Trail Valley Creek, bulk density was determined for each land cover class at replication of 3 (air-dried to constant mass), in the vicinity of the flux measurement locations for the top 0–7 cm of soil. To obtain bulk density values for below the 7-cm depth, needed for conversion of soil volumetric water content (VWC) to soil water-filled pore space

(WFPS) measured in soil profiles down to 30 cm, bulk density samples from the mineral active layer (A, B, and C horizon) were collected using a stainless-steel soil sampling ring (53 mm sample diameter; Eijkelkamp, Giesbeek, The Netherlands) and dried at 65°C. Organic layer sample volume was determined using the dimensions of carefully cut rectangular cuboids. WFPS was calculated based on bulk density, VWC and particle density[26].

For analysis of soil organic matter (SOM), soil dry mass, and soil C and N content, soil samples were oven dried to a constant mass at 65°C for 48 h. SOM was determined via loss on ignition at 550 °C in a muffle furnace. Analyses of soil C and N content as well as their stable isotopic signals took place at the Institute of Soil Science, University of Hanover, whereas all other soil and nutrient analyses were conducted in the facilities of the University of Eastern Finland, Department of Environmental and Biological Sciences. Soil C and N were determined from homogenized soil after milling at 30 rpm (Retsch MM301, Haan, Germany). Soil samples were measured for organic C and total N (TN) as well as for $\delta^{13}C$ via dry combustion in an Isotope cube element analyzer (Elementar Analysensysteme GmbH, Hanau, Germany) coupled to an IsoPrime 100 IRMS (IsoPrime Ltd, Cheadle Hulme, UK) after removing inorganic C by fumigation with HCl and subsequent neutralization over NaOH pellets (modified from Walthert et al.[27]). The measured $^{12}C/^{13}C$ ratios were corrected by calculating response factors from IAEA-derived standard compounds ($CaCO_3$, cellulose, caffein) and expressed in the delta notation ($\delta^{13}C$) related to the Vienna Peedee-Belemnite-Standard (0 ‰). Accordingly, the $^{14}N/^{15}N$ ratios are presented as $\delta^{13}N$ relative to the atmospheric isotope signature of N_2 gas (0 ‰). The $\delta^{13}C$ values of -25 to -28 ‰ are in the typical range of C3 vegetation derived soil organic matter and thus indicate complete removal of inorganic C. Positive $\delta^{15}N$ values (e.g., as observed at the Finnish sites) indicate excess N in the system leading to substantial losses of gaseous N species during N-cycling processes[28].

Methane and CO_2 isotope analysis

Soil gas samples for isotopic analysis were collected at the 10-cm depth following the procedure for soil pore gas sampling described above. Isotope samples were collected once for each collar during the year 2021 (Trail Valley Creek: June; Kilpisjärvi: August). Samples were transferred to 12-mL glass exetainers (Labco Exetainer®, Labco Ltd., Lampeter, UK) and stored at room temperature until analysis by cavity ring-down spectroscopy (Picarro G2201-i, Picarro Inc., Santa Clara, CA, USA). 19 mL of sample were introduced manually through a small sample isotope module (SSIM, 2021). The measured isotope values were corrected for instrument drift and concentration effects based on repeated measurements of an in-house reference gas at different dilutions. Data processing included (1) concentration correction for small dilutions with zero air during sample introduction (2) drift control based on the reference gas, and (3) correction of the concentration dependence of measured isotope ratios based on the standard dilutions. The concentration dependence was corrected by fitting a linear regression between measured isotope ratio and inverse concentration, with individual analysis weighted inversely to the standard deviation during the measurement to account for the lesser precision of measurements of lower concentrated dilutions.

To test if CH_4 oxidation contributed significantly to the soil CO_2 pool, isotope values of CO_2 were plotted against the inverse concentration (Keeling plot) representing a mixing model of variable amounts of soil-derived CO_2 with a constant background of atmospheric CO_2 (**Supplementary Fig. 11**). This plot represents a two-pool mixing model with a constant background concentration and isotope value (i.e., atmospheric CO_2) to which a second source of unknown $\delta^{13}C$–CO_2 was added. In this model, the $\delta^{13}C$–CO_2 value of this source can be estimated as the intercept of a linear regression in the plot. All points fell close to a single line, indicating little variation in the source $\delta^{13}C$–CO_2 values, which was estimated as -22.3±0.4 (2 x standard error) ‰. This is close to the $\delta^{13}C$-CO_2 of soil organic matter under C3-vegetation (~-27 ‰); the difference may have resulted from isotope discrimination due to diffusion out of the soil (i.e., ^{13}C becomes enriched in the residual CO_2 in soil). Soil CH_4, in contrast, was isotopically depleted (-50 to -35 ‰), with further discrimination (ca. 10–20 ‰) against ^{13}C during CH_4 oxidation. Any substantial contribution of CH_4 oxidation to the soil CO_2 pool would therefore have resulted in more negative $\delta^{13}C$–CO_2 values. We indicated such a scenario in **Supplementary Fig. 11**, where the dashed grey line indicates a 10 % contribution of CH_4-derived CO_2 with an assumed isotope value of -60 ‰. As all points fell well above this line, it is clear that CH_4 oxidation was only a marginal source of CO_2 in these soils. The conclusion of a negligible contribution fraction of

CH_4-oxidation to soil CO_2 concentrations is also supported by a simple mass balance: Soil CH_4 uptake was four orders of magnitude smaller than ER (**Fig. 2A–B**), or, in other words, ER was approximately 500-fold higher than CH_4 (0 to 750 mg CO_2 m^{-2} h^{-1} of ER vs. 0.00 to -0.15 mg CH_4 m^{-2} h^{-1} of CH_4 oxidation; **Fig. 2D**).

We further tested whether microbial methanogenesis occurred in the studied soils. We plotted the CH_4 concentration of each sample against its carbon isotope value (**Supplementary Fig. 11**) to compare the measured values to the trends expected for the oxidation of atmospheric CH_4 and mixing of atmospheric CH_4 with microbial CH_4 produced in soil. We compare atmospheric concentration (~1.9 ppm) and carbon isotope value (-48 ‰) to two scenarios: (i) inputs of ^{13}C-depleted microbial CH_4, (leading to increasing concentrations and decreasing $\delta^{13}C–CH_4$), and (ii) isotope fractionation due to microbial CH_4 oxidation and diffusion, which decreases concentrations and increases $\delta^{13}C–CH_4$. Methane oxidation is modeled following a Rayleigh distillation function with fractionation factors (alpha) of either 1.010 or 1.020. Admixture of microbial CH_4 is modeled using a simple mixing model with source isotope values of -60 and -80 ‰). Note that the two processes are not mutually exclusive; samples affected by both processes would fall above the plotted scenarios. As shown in **Supplementary Fig. 11**, most measured data points fell within the area expected for atmospheric CH_4 affected by CH_4 oxidation.

Together, our isotopic analyses show that (i) microbial methanogenesis contributed only marginal amounts of CH_4 and that (ii) CH_4 oxidation contributed only minimal amounts of CO_2.

Incubation experiments to determine the effects of temperature, moisture, and carbon addition on methane oxidation

Incubation experiment 1: Response of methane oxidation to temperature under field-moist conditions

To test for the temperature response of CH_4 consumption we conducted a soil incubation experiment at 4 °C and 20 °C with soils from Trail Valley Creek (Upland tundra and Polygon rim) and Finnish Lapland (Upland forest and Palsa II). Prior to incubation, soils from Trail Valley Creek were stored frozen, subsequently unfrozen at 4 °C, and left to acclimatize for one week, whereas soil samples from Finnish Lapland, collected four weeks before the incubation experiment, had been stored at 4 °C. We used biological replicates and replication followed those in *in situ* flux measurements. 30 mL of fresh soil were weighed into pre-sterilized, 120 mL incubation flasks (VWR, Radnor, PA, USA). A smaller volume was used in case of nine of the Trail Valley Creek samples, due to low sample amount. Samples were pre-incubated at the respective temperatures for 48 h prior to the start of the incubation experiment. During pre-incubation, flasks were covered with perforated parafilm, to prevent moisture loss but allow air exchange. At the start of the incubation the flasks, including three blanks, were flushed with ambient, outside air (measured concentration: 2.00 ppm CH_4, 421 ppm CO_2) before closing them with thick butyl rubber septa secured with screw caps (GL45, Glasgerätebau Ochs, Bovenden, Germany). 25 mL of gas were sampled at four time points for the Finnish soils (0 h, 2 h, 6 h, and 24 h after closing the flasks), and at six timepoints for the Trail Valley Creek soils (0 h, 1 h, 2 h, 4 h, 6 h, 24 h). In order not to disturb the diffusion gradient and artificially increase CH_4 uptake rates by creating overpressure, an equal amount of ambient air was added immediately after each sampling. Gas samples were analyzed via gas chromatography as described above.

Incubation experiment 2: Response of methane oxidation to temperature under dry and wet conditions

To further investigate the temperature sensitivity of CH_4 oxidation, which was not statistically significant under field-moist conditions and biological replication in incubation 1 (**Extended Data Fig. 9**), we conducted a second incubation experiment. For this purpose, we pooled soil samples from the five field replicates and incubated four technical replicates of each microsite to reduce the effect of high microsite variability masking a possible temperature effect. Due to limited availability of fresh soil from the Canadian sites, incubation experiment 2 was conducted with soils from Finnish Lapland. Soils were collected in the year 2022 within ca. 50 m of the sampling locations from 2021. Samples were collected from Upland forest, Palsa II, and Upland tundra land cover types. Soil sampling and storage followed the procedure described above. Incubations were done with fresh soil under 'dry' and 'wet' conditions. For the dry treatment, soils were air-dried to 20 % water-holding capacity (WHC). For the wet

treatment, deionized water was added to reach a WHC of 60 %. We used the same temperature scenarios as during incubation 1 (4°C and 20 °C). While sample amounts and incubation flasks were the same as during incubation 1, we used a portable gas analyzer (LI-7810, LI-COR Biotechnology, Lincoln, NE, USA) to measure flask headspace concentrations instead of manual sampling, to avoid CH_4 substrate depletion while measuring CH_4 consumption under ambient headspace conditions. The closure time was 2 minutes, and fluxes were calculated from the change in headspace concentrations over time using MATLAB version R2020b as described above for manual chamber measurements. Fluxes were converted to a soil dry mass basis.

Incubation experiment 3: Response of methane oxidation to carbon addition at different temperatures

To test for a direct effect of labile C addition on CH_4 oxidation and to explore the link between CH_4 uptake and ER observed under *in situ* conditions, we conducted a third incubation experiment. The procedure followed the one described for incubation 2 above. Incubations were done with fresh soil under field-moist conditions. Fluxes were measured from a "control" treatment at both temperatures (no C-addition), within 1 h after C-addition, and 24 h after C-addition. Labile C was added as glucose ($C_6H_{12}O_6$). The amount of added C was calculated per land cover type to match 0.3 % of the soil organic C content, corresponding to ca. 1460 µg C (g DW^{-1}) in Palsa II and 120 µg C (g DW^{-1}) in the upland soils consisting of a homogenized sample of the organic and mineral soil layer.

Auxiliary data collection

Accompanying manual chamber flux measurements, thaw depth, surface soil moisture (0–6 cm depth), air and soil temperature (5 cm depth) were recorded next to each flux collar at the same time as manual chamber flux measurements. Surface soil moisture (as %VWC) was measured with a HydroSense II probe and display (CS-658 and HS2, Campbell Scientific Inc.) in years 2018 and 2019, and using an ML-3 Thetaprobe connected to a HH2 moisture sensor (Eijkelkamp) in 2021. The Thetaprobe was site-calibrated with Trail Valley Creek soils during summer 2021 according to the manufacturer recommendation. The HydroSense used a custom-made calibration for organic soils, and built-in calibration for mineral soils. To make sure measurements were comparable, simultaneous measurements with both sensors were conducted once at all collars at Trail Valley Creek in June 2021. For investigating correlations between manual chamber measurements and environmental variables, these simultaneous VWC measurements by the two instruments were used to develop a function to convert soil moisture measurements collected with the HydroSense Probe to match the ML-3 sensor output ($y = 0.968x^{3.4763}$; $R^2 = 0.81$). Soil temperature at Trail Valley Creek was recorded at 30-min intervals using HOBO Pendant sensors (UA-001-08, Onset, Bourne, MA, USA) installed next to each flux collar. At all other sites, soil temperature was measured using a handheld thermometer (TM-80N with K-type thermocouple probe, Tenmars Electronics, Taipei City, Taiwan).

For continuous measurements of soil temperature, VWC, electrical conductivity, and soil oxygen concentration to accompany continuous flux measurements collected with automated chambers at Trail Valley Creek, we installed sensors at three depths (10 cm, 20 cm, 30 cm) in one soil profile per vegetation type (Lichen, Shrub, Tussock) using soil moisture probes (CS650L, Water Content Reflectometer Plus with 30cm rods, Campbell Scientific Inc.) and oxygen probes (Yuasa KE-25; Figaro, Japan). Oxygen sensors were calibrated in ambient air and waterproofed by placing them in a silicon tube sealed with rubber septum. To cover the spatial variability, soil temperature and moisture (CS655-L Water Content Reflectometer Plus, Campbell Scientific Inc., Logan, UT, USA) were also measured next to each of the nine opaque chambers (n = 3 per vegetation type) using vertically inserted, 12-cm long rods. According to the manufacturer, soil moisture measurements corresponded to the 0–12 cm profile, whereas soil temperature was measured at the top of the rods, corresponding to surface soil temperature (below the vegetation and lichen cover).

Meteorological variables were collected at nearby automated weather stations located within a 50-m radius of the automated chamber set-up. Meteorological variables were obtained from a weather station operated by the Meteorological Survey of Canada (MSC)[3]. For periods where MSC data were missing or removed for quality control purposes, data collected at two independent weather stations operated by the Trail Valley Creek Research Station,

utilizing identical meteorological sensors albeit at slightly varying heights, were used to gap-fill. Air temperature and relative humidity were measured at 2 m above the ground surface using a temperature and humidity probe (HMP155, Campbell Scientific Inc.). Wind speed was measured with a wind monitor (6.1 m height; Model 05103 Wind Monitor, RM Young, Michigan, USA). Rainfall was measured using a Geonor weighted precipitation gauge equipped with a single alter windshield (Geonor T-200B, Bærum Norway). Rainfall during the growing season was additionally measured using a tipping bucket (TE525M, Campbell Scientific Inc.). PAR was measured at 4.1 m using PQS1 sensors (Kipp Zonen, Delft Netherlands).

Statistical analyses

Linear-mixed-effects models

Linear-mixed-effects models (R-package *LME4*)[29] were applied to the automated chamber data to identify the contribution of environmental variables, as well as their interacting effect, on CH_4 consumption rates at Trail Valley Creek. Prior to analyses, all data were inspected visually for their distribution via histograms, and density and QQ-plots. Automated chamber data were transformed if they deviated too much from normal distribution, which was the case for Shrub and Tussock (**Supplementary Table 13**). We constructed four separate models: Model 1 included all three vegetation types (Lichen, Shrub, Tussock), but was restricted to CH_4 fluxes < 0 (uptake) and with outliers in the 1 % percentile removed to obtain normal distribution. Models 2–4 were models for the three individual vegetation types (Lichen, Shrub, Tussock). All models included the chamber ID as a random effect to account for repeated measures on the same chamber collar, and data were pooled for both measurement years. Multi-collinearity was checked using variance-inflation factors as described earlier[30]. The total explanatory power of the linear-mixed-effects models was moderate (R^2 = 0.20–0.53). A large portion of the variability in fluxes was explained by site variability as expressed by the explanatory power of random effects (11–30 %), resulting in a poor explanatory power of the fixed effects structure (R^2 = 0.09–0.38; **Supplementary Table 13**).

Random Forest models

The poor predictive performance of linear-mixed effects models implies complex, non-linear relationships between environmental variables and their influence on CH_4 fluxes. We therefore applied a Random Forest (RF) model, better suited for large datasets involving non-normal and non-linear distribution and relationships[31]. RF analysis was performed using the R package *randomForest*[32], and the modeled relationships were assessed with partial dependence plots created using package *pdp*[33]. As soil temperature and moisture were only measured for the opaque chambers (n = 9) and PAR only in the transparent chambers (n = 9), we calculated soil temperature and moisture for transparent chambers as the mean of each vegetation type measured with opaque chambers (n = 3) and used the same method to determine PAR values for the remaining 9 opaque chambers in RF analyses. We used 500 trees to construct the random forests ('ntree' = 500), and the number of variables tried at each split was determined individually for each model using function '*tuneRF*' to find the optimal 'mtry' value with the smallest out-of-bag error (**Supplementary Table 8**). Default values were used for 'nodesize' and 'maxnodes' parameters. Variable importance was assessed using the average increase in node purity of the regression trees based on splitting on the various environmental variables[34]. The 'percent variance explained' (**Supplementary Table 8**) is a measure of how well the model explains the target variance of the training dataset[32].
First, RF models were created for all vegetation types (including only fluxes < 0), as well as separate models for the individual vegetation types. Data were further split into two datasets, resulting in eight main random forest models (Table S7):

1) Flux data from 2019 and 2021, with a smaller set of environmental variables (soil temperature and moisture only measured in the surface soil; 6 predictors). Highly-correlated variables (Spearman correlation coefficient > 0.7) were excluded from this model.

2) Flux data from 2021, during which a larger set of environmental variables were measured (including soil temperature and moisture in the soil profile down to 30 cm; 18 predictors). This model included some highly correlated predictors (Spearman correlation coefficient > 0.7).

These models were created for hourly measured fluxes. To ensure robustness of the RF analysis, and to identify if the dominant predictors change over different temporal scales as observed for CH_4 emissions by Knox et al.[35], we repeated Model 1 for daily and weekly aggregated data. Aggregation to daily and weekly values did not majorly change the model output but indicated a slightly higher relative importance of temperature (**Extended Data Fig. 3**). We then repeated Model 1 for a subset of data measured during a consistently dry period in 2021 (DOY 182–243), using surface soil temperature, temperature at the 10-cm, 20-cm, and 30-cm depths. These models showed that when the relative importance of soil moisture is reduced during comparably dry conditions, temperature slightly gains in importance, though still frequently overruled by other abiotic variables (WFPS, PAR, wind speed) (**Supplementary Fig. 5**). Further RF models were created for each chamber to explore the relative importance of the two established controls on CH_4 uptake, temperature, and soil moisture[36–38], as well as the additional explanatory power of ecosystem respiration (ER). These RF models were created for each replicate chamber to control for microsite heterogeneity, split by early-, peak-, and late summer. To test whether the additional explanatory power of ER was statistically significant, we ran each RF model 100 times and compared the model outputs (**Supplementary Table 14**). Constructing RF models with the default values and optimized 'mtry' value in *randomForest* generally has a low risk of overfitting[31]. Nonetheless, we sporadically checked for overfitting by splitting the datasets into training data and validation data at a 7:3 ratio. We used RMSE values of the RF models using training and validation data to assess whether the models were overfitting, where a significantly higher RMSE when running the model with validation data would indicate model overfitting. The RMSE of models using validation data was typically low and comparable to RMSE of models run on the training datasets. For models split by vegetation type (**Fig. 3A**) RMSE values for models using training and validation data, respectively, were 0.0090 and 0.0091 (Lichen), 0.0191 and 0.0195 (Shrub), 0.0274 and 0.0244 (Tussock). For models using only soil moisture, temperature, and ER split by chamber and month (**Fig. 3B, Extended Data Fig. 6**) RMSE values were 0.0027–0.0363 for models run on training data, and 0.0025–0.0394 for models run on validation data (**Supplementary Table 14**).

Finally, we applied the RF model approach to CH_4 fluxes measured with manual chambers across Canadian and Finnish sites for which soil analyses were performed (Trail Valley Creek: Upland tundra, Polygon rim; Kilpisjärvi: Upland forest, Palsa II). The RF analysis for the manual chamber dataset included not only abiotic variables (as for the automated chambers) but also soil characteristics as well as soil biogeochemical properties and nutrient turnover rates. Principal component analysis (PCA) as well as correlation tests (Spearman) were conducted prior to RF modeling to identify variables closely related to CH_4 uptake for inclusion in the RF model (**Supplementary Fig. 12**). We used packages *FactoMineR*[39] and *factoextra*[40] in the PCA. Biplots were created both for the manual chamber dataset as well as for the automated chamber dataset.

Lagged interactions via transfer entropy

We used transfer entropy (TE) to detect lagged interactions between fluxes measured by automated chambers and environmental variables at Trail Valley Creek. The method followed the approach described by Chamberlain et al.[41]. TE is a method based on information theory that quantifies the extent to which knowledge of a variable X reduces the uncertainty of the present state of Y given the past knowledge of Y [ref 42]. For observations collected at regular intervals, TE at a specific lag k can be defined from joint and conditional probability functions incorporating time lags as follows[43]:

$$TE(X \to Y, k) = \sum_{y_t, y_{t-1}, x_{t-k}} p(y_t, y_{t-1}, x_{t-k}) \log \frac{p(y_t, | y_{t-1}, x_{t-k})}{p(y_t, | y_{t-1})} \quad (2)$$

If X at lag k contains no additional information to predict Y of what is already contained in Y, the ratio inside the log of Eq. (2) is one, and TE equals zero; otherwise, TE is bigger than zero. The significance of TE is evaluated as the 95th percentile obtained from 250 independent Monte Carlo reshuffling of variable X. To ensure stationarity and

reduce long-term trends, the analyses were performed on changes in fluxes, the differences between consecutive observations. Data were pooled for each vegetation type after normalizing each series to unit variance and analyzed for each period (June, July, August). The probabilities in equation (2) were computed using a simple box-counting algorithm based on marginal equiquantization, which means that the bins of the marginal distribution are not defined equidistantly but so that there is the same number of data points in each marginal bin[44]. For n variables the number of bins should be less than $\sqrt[n+1]{N}$, where N is the number of data points[45]. The reshuffling is computed along each hour of the day to preserve the diurnal fluctuation.

We investigated the presence of lagged interactions between CH_4 flux and surface soil temperature, temperature measured in 10 cm, 20 cm, and 30 cm, as well as with PAR, and repeated the same analysis with ER. Transfer entropy analysis generally revealed only weak lagged effects of temperature. Lag interactions (commonly 3–4 h) occurred with surface soil temperature (**Fig. 3B, Supplementary Fig. 13**). During June, a diel pattern of lagged interactions occurred between CH_4 flux and deeper soil temperature (10 cm) for Lichen and Tussock (**Supplementary Fig. 14**). No significant lagged effects were detected with PAR (**Supplementary Fig. 15**). It has to be noted that soil temperatures in 10 cm, 20 cm, and 30 cm were only measured during the 2021 measurement season and the resulting TE appears noisier due to fewer observations, particularly during July (**Supplementary Fig. 14**). Similarly, relationships with ER are noisier, as ER was directly measured only in the opaque chambers, reducing the number of observations.

References for Supplementary Material

1. Olefeldt, D. *et al.* The Boreal–Arctic Wetland and Lake Dataset (BAWLD). *Earth Syst Sci Data* **13**, 5127–5149 (2021).
2. ESA. Land Cover CCI Product User Guide Version 2. Tech. Rep. (2017). Available at: maps.elie.ucl.ac.be/CCI/viewer/download/ESACCI-LC-Ph2-PUGv2_2.0.pdf.
3. Environment and Climate Change Canada. Canadian climate normals. (2022). Available at http:// climate.weather.gc.ca/climate_normals/.
4. Raynolds, M. K. *et al.* A raster version of the Circumpolar Arctic Vegetation Map (CAVM). *Remote Sens Environ* **232**, 111297 (2019).
5. Walker, D. A. *et al.* The circumpolar Arctic vegetation map. *Journal of Vegetation Science* **16**, 267–282 (2005).
6. Grünberg, I., Wilcox, E. J., Zwieback, S., Marsh, P. & Boike, J. Linking tundra vegetation, snow, soil temperature, and permafrost. *Biogeosciences* **17**, 4261–4279 (2020).
7. Burn, C. R. & Kokelj, S. V. The environment and permafrost of the Mackenzie Delta area. *Permafr Periglac Process* **20**, 83–105 (2009).
8. Walker, B., Wilcox, E. J. & Marsh, P. Accuracy assessment of late winter snow depth mapping for tundra environments using Structure-from-Motion photogrammetry. *Arct Sci* **7**, 588–604 (2020).
9. Wilcox, E. J. *et al.* Tundra shrub expansion may amplify permafrost thaw by advancing snowmelt timing. *Arct Sci* **5**, 202–217 (2019).
10. Krogh, S. A. & Pomeroy, J. W. Recent changes to the hydrological cycle of an Arctic basin at the tundra–taiga transition. *Hydrol Earth Syst Sci* **22**, 3993–4014 (2018).
11. Dearborn, K. D., Wallace, C. A., Patankar, R. & Baltzer, J. L. Permafrost thaw in boreal peatlands is rapidly altering forest community composition. *Journal of Ecology* **109**, 1452–1467 (2021).

12. Quinton, W. *et al.* A synthesis of three decades of hydrological research at Scotty Creek, NWT, Canada. *Hydrol Earth Syst Sci* **23**, 2015–2039 (2019).

13. Standen, K. M. & Baltzer, J. L. Permafrost condition determines plant community composition and community-level foliar functional traits in a boreal peatland. *Ecol Evol* **11**, 10133–10146 (2021).

14. Pirinen, P. *et al.* Tilastoja suomen ilmastosta 1981–2010. *The Finnish Meteorological Institute* **1**, 96 pp. (2012).

15. Seppälä, M. Palsa mires in Finland. *The Finnish environment* **23**, 155–162 (2006).

16. Seppälä, M. Synthesis of studies of palsa formation underlining the importance of local environmental and physical characteristics. *Quat Res* **75**, 366–370 (2011).

17. Voigt, C. *et al.* Increased nitrous oxide emissions from Arctic peatlands after permafrost thaw. *Proc Natl Acad Sci U S A* **114**, 6238–6243 (2017).

18. Aalto, J., Venäläinen, A., Heikkinen, R. K. & Luoto, M. Potential for extreme loss in high-latitude Earth surface processes due to climate change. *Geophys Res Lett* **41**, 3914–3924 (2014).

19. Borge, A. F., Westermann, S., Solheim, I. & Etzelmüller, B. Strong degradation of palsas and peat plateaus in northern Norway during the last 60 years. *Cryosphere* **11**, 1–16 (2017).

20. Happonen, K., Virkkala, A., Kemppinen, J., Niittynen, P. & Luoto, M. Relationships between above-ground plant traits and carbon cycling in tundra plant communities. *Journal of Ecology* **110**, 700–716 (2022).

21. Eckhardt, T. *et al.* Partitioning net ecosystem exchange of CO 2 on the pedon scale in the Lena River Delta, Siberia. *Biogeosciences* **16**, 1543–1562 (2019).

22. Brown, J., Ferrians Jr, O. J., Heginbottom, J. A. & Melnikov, E. S. (2002). Circum-Arctic map of permafrost and ground-ice conditions.

23. Dinerstein, E. *et al.* An Ecoregion-Based Approach to Protecting Half the Terrestrial Realm. *Bioscience* **67**, 534–545 (2017).

24. Marushchak, M. E. *et al.* Thawing Yedoma permafrost is a neglected nitrous oxide source. *Nat Commun* **12**, 7107 (2021).

25. Lan, X., Thoning, K.W., Dlugokencky, E. J. Trends in globally-averaged CH_4, N_2O, and SF_6 determined from NOAA Global Monitoring Laboratory measurements. (2022). Version 2022-12. Available at: https://gml.noaa.gov/hats/combined/N2O.html.

26. Okruszko, H. Determination of specific gravity of hydrogenic soils on the basis of their mineral particles content. Wiadomosci Instytutu Melioracji i Uzytkow Zielonych, 10.1: 47–54 (in Polish with English summary). In: *Organic soils and peat materials for sustainable agriculture* (eds. Léon-Etienne, P. & Ilnicki, P.) (CRC Press LLC, 2003).

27. Walthert, L. *et al.* Determination of organic and inorganic carbon, $\delta^{13}C$, and nitrogen in soils containing carbonates after acid fumigation with HCl. *Journal of Plant Nutrition and Soil Science* **173**, 207–216 (2010).

28. Bai, E., Houlton, B. Z. & Wang, Y. P. Isotopic identification of nitrogen hotspots across natural terrestrial ecosystems. *Biogeosciences* **9**, 3287–3304 (2012).

29. Bates, D., Mächler, M., Bolker, B. & Walker, S. Fitting Linear Mixed-Effects Models Using lme4. *J Stat Softw* **67**, 1–48 (2015).

30. Voigt, C. *et al.* Warming of subarctic tundra increases emissions of all three important greenhouse gases - carbon dioxide, methane, and nitrous oxide. *Glob Chang Biol* **23**, 3121–3138 (2017).

31. Breiman, L. Random forests. *Mach Learn* **45**, 5–32 (2001).

32. Liaw, A. & Wiener, M. *Classification and Regression by randomForest*. *R news* **2**, 18–22 (2002).

33. Greenwell, B. M. pdp: an R Package for constructing partial dependence plots. *R J* **9**, 421 (2017).

34. Louppe, G., Wehenkel, L., Sutera, A. & Geurts, P. Understanding variable importances in forests of randomized trees. *Adv Neural Inf Process Syst* **26**, 1–9 (2013).

35. Knox, S. H. *et al.* Identifying dominant environmental predictors of freshwater wetland methane fluxes across diurnal to seasonal time scales. *Glob Chang Biol* **27**, 3582–3604 (2021).

36. King, G. Responses of atmospheric methane consumption by soils to global climate change. *Glob Chang Biol* **3**, 351–362 (1997).

37. Smith, K. A. *et al.* Oxidation of atmospheric methane in Northern European soils, comparison with other ecosystems, and uncertainties in the global terrestrial sink. *Glob Chang Biol* **6**, 791–803 (2000).

38. Tate, K. R. Soil methane oxidation and land-use change–from process to mitigation. *Soil Biol Biochem* **80**, 260–272 (2015).

39. Lê, S., Josse, J. & Husson, F. FactoMineR: an R package for multivariate analysis. *J Stat Softw* **25**, 1–18 (2008).

40. Kassambara, A. & Mundt, F. factoextra: Extract and Visualize the Results of Multivariate Data Analyses. R Package Version 1.6 (2017).

41. Chamberlain, S. D. *et al.* Effect of drought-induced salinization on wetland methane emissions, gross ecosystem productivity, and their interactions. *Ecosystems* **23**, 675–688 (2020).

42. Schreiber, T. Measuring information transfer. *Phys Rev Lett* **85**, 461 (2000).

43. Kumar, P. & Ruddell, B. L. Information driven ecohydrologic self-organization. *Entropy* **12**, 2085–2096 (2010).

44. Paluš, M. From nonlinearity to causality: statistical testing and inference of physical mechanisms underlying complex dynamics. *Contemp Phys* **48**, 307–348 (2007).

45. Paluš, M. Testing for nonlinearity using redundancies: Quantitative and qualitative aspects. *Physica D* **80**, 186–205 (1995).
