# Supplementary material for: Arctic soil methane sink increases with drier conditions and higher ecosystem respiration
Source: Nat Clim Chang. 2023 Aug 31;13(10):1095–104. doi: 10.1038/s41558-023-01785-3 (PMC10550823; doi:10.1038/s41558-023-01785-3)
Supplement: Supplementary file 2 — Reporting Summary [file 41558_2023_1785_MOESM2_ESM.pdf]

nature portfolio

Corresponding author(s): Dr. Carolina Voigt

Last updated by author(s): Jul 17, 2023

Reporting Summary

Nature Portfolio wishes to improve the reproducibility of the work that we publish. This form provides structure for consistency and transparency in reporting. For further information on Nature Portfolio policies, see our Editorial Policies and the Editorial Policy Checklist.

Statistics

For all statistical analyses, confirm that the following items are present in the figure legend, table legend, main text, or Methods section.

n/a	Confirmed	
☐	☒	The exact sample size (*n*) for each experimental group/condition, given as a discrete number and unit of measurement
☐	☒	A statement on whether measurements were taken from distinct samples or whether the same sample was measured repeatedly
☐	☒	The statistical test(s) used AND whether they are one- or two-sided<br>*Only common tests should be described solely by name; describe more complex techniques in the Methods section.*
☐	☒	A description of all covariates tested
☐	☒	A description of any assumptions or corrections, such as tests of normality and adjustment for multiple comparisons
☐	☒	A full description of the statistical parameters including central tendency (e.g. means) or other basic estimates (e.g. regression coefficient) AND variation (e.g. standard deviation) or associated estimates of uncertainty (e.g. confidence intervals)
☐	☒	For null hypothesis testing, the test statistic (e.g. *F*, *t*, *r*) with confidence intervals, effect sizes, degrees of freedom and *P* value noted<br>*Give P values as exact values whenever suitable.*
☒	☐	For Bayesian analysis, information on the choice of priors and Markov chain Monte Carlo settings
☒	☐	For hierarchical and complex designs, identification of the appropriate level for tests and full reporting of outcomes
☐	☒	Estimates of effect sizes (e.g. Cohen's *d*, Pearson's *r*), indicating how they were calculated

Our web collection on statistics for biologists contains articles on many of the points above.

Software and code

Policy information about availability of computer code

Data collection

Greenhouse gas concentrations measured with the automated chamber system were recorded continuously and the switching of the gas flow between the 18 chambers as well as lid movement (opening and closing of chambers) were controlled by a central computer. All data were recorded using common dataloggers with multiplexers (Campbell Scientific Inc.). Recordings of greenhouse gas concentration measurements and auxiliary, environmental variables (temperature, soil moisture, photosynthetically active radiation), recorded by a separate datalogger, were merged into one file for data analyses. An example of a merged data file is provided with the code for this article.

Data analysis

Automated chamber flux data were processed with various routines developed in-house in the MATLAB computing environment, version R2020b (The MathWorks Inc., Natick, MA, USA), and the code is made available alongside this article (https://github.com/znesic/Voigt2023_CH4_uptake). The MATLAB algorithm used common formulas for flux calculation as described in the LI-8100A user manual (available at: https://www.licor.com/env/support/LI-8100A/manuals.html). However, the code was designed specifically for the automated chamber system at our site and allowed for direct processing (flux calculation) as well as initial quality control of input data files. The individual parts of the code, instructions on how to run the code, as well as example raw data files have been deposited in GitHub (https://github.com/znesic/Voigt2023_CH4_uptake). Manual chamber fluxes were processed using a published MATLAB algorithm (Eckhardt et al., 2019). All statistical analyses were performed in R, version 4.2.2, utilizing the following packages: FSA, LME4, RandomForest, multcompView, reshape, FactoMineR, factoextra. The R-codes used for Random Forest models are available on Zenodo (doi: 10.5281/zenodo.8152386).

References:
Eckhardt, T., Knoblauch, C., Kutzbach, L., Holl, D., Simpson, G., Abakumov, E., & Pfeiffer, E.-M. (2019). Partitioning net ecosystem exchange of CO2 on the pedon scale in the Lena River Delta, Siberia. Biogeosciences, 16(7), 1543–1562.

For manuscripts utilizing custom algorithms or software that are central to the research but not yet described in published literature, software must be made available to editors and reviewers. We strongly encourage code deposition in a community repository (e.g. GitHub). See the Nature Portfolio guidelines for submitting code & software for further information.

Data

Policy information about availability of data

All manuscripts must include a data availability statement. This statement should provide the following information, where applicable:

- Accession codes, unique identifiers, or web links for publicly available datasets
- A description of any restrictions on data availability
- For clinical datasets or third party data, please ensure that the statement adheres to our policy

Processed data files of fluxes and auxiliary measurements at high temporal resolution (automated chamber measurements) as well as high spatial resolution (manual chamber measurements) to reproduce the reported findings have been deposited in PANGAEA (doi: 10.1594/PANGAEA.953120).

Human research participants

Policy information about studies involving human research participants and Sex and Gender in Research.

Reporting on sex and gender	not applicable.
Population characteristics	not applicable.
Recruitment	not applicable.
Ethics oversight	not applicable.

Note that full information on the approval of the study protocol must also be provided in the manuscript.

Field-specific reporting

Please select the one below that is the best fit for your research. If you are not sure, read the appropriate sections before making your selection.

☐ Life sciences ☐ Behavioural & social sciences ☒ Ecological, evolutionary & environmental sciences

For a reference copy of the document with all sections, see nature.com/documents/nr-reporting-summary-flat.pdf

Ecological, evolutionary & environmental sciences study design

All studies must disclose on these points even when the disclosure is negative.

Study description	The study is based on field measurements of fluxes at high temporal and spatial resolution. For fluxes at high temporal resolution, we established an automated chamber system at Trail Valley Creek, an upland tundra site on continuous permafrost in the western Canadian Arctic. There, hourly fluxes were recorded over two growing seasons from 18 plots installed on three vegetation types (6 chambers per vegetation type): dwarf-shrub tundra with lichen cover where vascular plants were absent ('Lichen'), as well as more productive sites with deciduous and evergreen dwarf shrub ('Shrub') or tussock coverage ('Tussock'). To judge the spatial representativeness of these site-specific measurements we conducted campaign-based measurements at three additional sites across permafrost zones in the Canadian and European Arctic (Havikpak Creek, Scotty Creek, Kilpisjärvi). Manual chamber measurements were conducted on 5 landcover types (upland tundra, polygonal tundra, upland forest, peat plateau, palsa) and 1-3 vegetation types in each land cover (Lichen, Shrub, Tussock) at a typical replication of 5, resulting in 88 individual plots. Additionally, auxiliary variables were measured for both the automated chamber measurements (site meteorology, soil moisture, temperature, soil oxygen concentrations) and the manual chamber measurements (soil biogeochemistry, soil physical chemical properties, soil gas concentrations, soil incubations for sensitivity of CH_4 oxidation to temperature, soil moisture, and addition of labile carbon). Detailed soil analyses were conducted only at Trail Valley Creek and Kilpisjärvi.
Research sample	Trail Valley Creek was selected as an autochamber site because it was the only accessible site in the Western Canadian Arctic providing the necessary logistics, such as sufficient power supply, that realistically allowed us to operate an automated chamber system. Trail Valley Creek is a typical upland tundra site representative of other sites displaying CH_4 uptake in the Arctic. All other sites, where manual chamber measurements were conducted, are typical, well-drained upland tundra, upland forest, and permafrost peatland sites located in the Northern circumpolar permafrost region. Trail Valley Creek as well as Kilpisjärvi, the two intensively sampled sites in terms of soil biogeochemical data, have been the subject of previous studies on site hydrology, permafrost thaw and biogeochemistry, and background data such as site meteorological measurements are available.
Sampling strategy	Fluxes of CH_4 (and CO_2) at high temporal resolution were measured with a newly established, automated chamber system at Trail Valley Creek, following a chamber design described previously (Gaumont-Guay et al., 2009; Lai et al., 2012). Fluxes were measured during the growing season (June–August 2019 and 2021). For obvious logistical, technical, and financial reasons, we were not able to operate the automated chambers during the winter, but we made an effort to include the spring green-up and autumn senescence periods, as we acknowledge that non-growing season fluxes can be important for annual flux budgets (Treat et al., 2018), although the importance of atmospheric CH_4 uptake outside the growing season is unresolved. Automated fluxes were recorded hourly per chamber, resulting in 44 848 CH_4 flux measurement points after data cleaning from 18 plots representing 3 tundra vegetation types.

Half of the automated chambers were transparent, while the other half was equipped with an opaque cover. Since we did not observe marked differences in chamber temperature and flux, we consider these as 6 independent biological replicates for CH_4. Fluxes of CO_2 were determined alongside CH_4, where the opaque chambers (n = 3 per vegetation type, 9 opaque chambers in total) represent direct observations of ecosystem respiration. Auxiliary data associated with automated flux measurements were collected as follows: soil moisture and temperature in the surface soil (n = 3 per vegetation type), photosynthetically active radiation inside the chambers (n = 3 per vegetation type), soil profile data of oxygen, temperature, moisture (n = 1 per vegetation type for the depths 10 cm, 20 cm, 30 cm), site meteorology (n=1).

Manual chamber measurements at Trail Valley Creek were conducted on the same upland tundra vegetation types as the automated chambers (Lichen, Shrub, Tussock) in replication of 5 and additionally on polygon rims (Lichen only, as vascular plants were absent) in replication of 3, yielding a total of 18 manual flux plots. At all other sites, manual chamber measurements were conducted only on representative lichen and shrub-dominated plots, identified as atmospheric CH_4 sinks at Trail Valley Creek. As a default, manual flux measurements and associated auxiliary measurements were conducted at replication of 5, with the exception of Havikpak Creek (n = 10 for Shrub) and Scotty Creek (n = 2 for Shrub and n = 3 for Lichen). The number of plots per site and land cover type were as follows: Scotty Creek (5), Havikpak Creek (15), Kilpisjärvi palsa (30), Kilpisjärvi upland tundra (10), Kilpisjärvi upland forest (10). Auxiliary data associated with manual chamber flux measurements were collected as follows: measurements conducted in or next to each flux plot at replication described above were soil pore gas concentrations (3-4 depths along the soil profile, all sites), plant-available nitrogen (Trail Valley Creek only), soil moisture, soil temperature, thaw depth, greenness. Soil physical-chemical properties and soil biogeochemical measurements (e.g., soil extractable nutrients and dissolved organic carbon, soil incubations, soil carbon and nitrogen, soil bulk isotopic signal) were determined only for the following sites: Trail Valley Creek (all land covers), Kilpisjärvi upland forest, Kilpisjärvi palsa. One subset of incubations included also Kilpisjärvi upland tundra. Most of the manual chamber measurements were conducted during the main growing season (June-August). Only for Scotty Creek, manual chamber measurements took place in mid-September (due to logistical reasons). However, Scotty Creek is the southernmost site and we consider flux measurements comparable to the ones measured at the other sites.

The number of spatial field replicates (typically n = 5) was guided by our previous experience from similar field studies (Voigt et al., 2017). In a study design like ours, inclusion of sufficient number of different land cover and vegetation types was more important than increasing the number of replicates for revealing trends in the CH_4 uptake dynamics. This can be justified, because the spatial variability in CH_4 uptake observed here is smaller than for other fluxes, for example N_2O (Voigt et al., 2020), and plot selection for manual chamber studies usually requires compromising between the number of studied surface types and the replicate number.

References:
Gaumont-Guay, D., Black, T. A., McCaughey, H., Barr, A. G., Krishnan, P., Jassal, R. S., & Nesic, Z. (2009). Soil CO2 efflux in contrasting boreal deciduous and coniferous stands and its contribution to the ecosystem carbon balance. Global Change Biology, 15(5), 1302–1319.
Lai, D. Y. F., Roulet, N. T., Humphreys, E. R., Moore, T. R., & Dalva, M. (2012). The effect of atmospheric turbulence and chamber deployment period on autochamber CO 2 and CH 4 flux measurements in an ombrotrophic peatland. Biogeosciences, 9(8), 3305–3322.
Treat, C. C., Bloom, A. A., & Marushchak, M. E. (2018). Nongrowing season methane emissions-a significant component of annual emissions across northern ecosystems. Global Change Biology, 24(8), 3331–3343. https://doi.org/10.1111/gcb.14137
Voigt, C., Lamprecht, R. E., Marushchak, M. E., Lind, S. E., Novakovskiy, A., Aurela, M., Martikainen, P. J., & Biasi, C. (2017). Warming of subarctic tundra increases emissions of all three important greenhouse gases - carbon dioxide, methane, and nitrous oxide. Global Change Biology, 23(8), 3121–3138. https://doi.org/10.1111/gcb.13563
Voigt, C., Marushchak, M. E., Abbott, B. W., Biasi, C., Elberling, B., Siciliano, S. D., Sonnentag, O., Stewart, K. J., Yang, Y., & Martikainen, P. J. (2020). Nitrous oxide emissions from permafrost-affected soils. Nature Reviews Earth & Environment, 1(8), 420–434. https://doi.org/10.1038/s43017-020-0063-9

Data collection

Field data at the Canadian study sites were mainly collected by the University of Montreal Team led by C. Voigt (primary affiliation at University of Montreal 2018-2020, and primary affiliation at University of Eastern Finland 2020-2023). Field work costs between 2018-2020 were covered by O. Sonnentag and P. Marsh (University of Montreal/Wilfrid Laurier University), and from 2021 onwards for C. Voigt by the University of Eastern Finland/Academ of Finland). Assistance in data collection, as well as logistical support, funding and maintenance for the automated chamber system infrastructure was provided by colleagues from the Cold Regions Research Centre, Wilfrid Laurier University. The field work in Finland was led by C. Voigt and supported by colleagues from University of Jyväskylä and Woodwell Climate Research Center. Laboratory work was conducted at the University of Eastern Finland, with the exception of isotopic analyses which was conducted at the University of Hanover.

Measurement of field fluxes were conducted with chamber methods, where the automated chambers followed a design described previously (Gaumont-Guay et al., 2009; Lai et al., 2012), but the system was adapted to work at Trail Valley Creek. Gas concentrations in the chamber headspace were measured at 1 s intervals with a Los Gatos Research (LGR) Enhanced Performance greenhouse gas analyzer (Rackmount GGA-24EP 911-0010, Los Gatos Inc; Los Gatos, CA, USA), enhanced for thermal stability to provide ultra-stable readings. We used a measurement frequency of 1 Hz (Enhanced performance, slow flow) and an external three-head diaphragm pump (N-920, 1.2 s flow-through time, 0.83 Hz, KNF Neuberger Inc., Trenton, NJ, USA), bypassing the internal pump of the analyzer to achieve a slow flow response. Manual chamber fluxes were measured at 1 s intervals with a Picarro gas analyzer (G4301 GasScouter, Picarro Inc., Santa Clara, CA, USA) at all sites except Scotty Creek, where we used an LGR instrument (LGR U-GGA-915 478 Ultraportable, Los Gatos Inc; Los Gatos, CA, USA). All flux measurements are described in detail in the Methods section and the Supplementary Methods.

Accompanying manual chamber flux measurements, thaw depth, surface soil moisture (0–6 cm depth), air and soil temperature (5 cm depth) were recorded next to each flux collar concurrent with manual chamber flux measurements. For continuous measurements of soil temperature, volumetric water content, and soil oxygen concentration to accompany automated chamber measurements at Trail Valley Creek, we installed sensors at three depths (10 cm, 20 cm, 30 cm) in one soil profile per vegetation type using soil moisture probes (CS650L, Water Content Reflectometer Plus with 582 30cm rods, Campbell Scientific Inc.) and oxygen probes (Yuasa KE-25, Figaro Engineering Inc., Osaka, Japan).

Soil gas concentrations were analyzed with a gas chromatograph (Agilent 7890B Agilent Technologies, Santa Clara, CA, USA) equipped with an autosampler (Gilson Inc., WI, Middleton, USA), an electron capture detector (ECD) for N_2O, a flame ionization

detector (FID) for methane (CH4), and a thermal conductivity detector (TCD) for CO2. Soil gas sampling and analysis followed the general description provided by Marushchak et al. (2021).

Soil samples were collected in 0-10 cm at the intensively sampled sites Trail Valley Creek (all vegetation types and land covers) and Kilpisjärvi (upland forest and palsa). Soil sampling procedure and physical-chemical analyses are described in detail in the Methods and Supplementary Methods sections and followed established methods (Marushchak et al., 2011, 2021; Voigt et al., 2017).

For vegetation greenness, collar photographs were taken weekly to bi-weekly at Trail Valley Creek and once at all other sites. Collar greenness was calculated using the Canopeo beta version Foliage (version 1.0) (Patrignani & Ochsner, 2015).

Amounts of the plant-available nitrogen forms ammonium and nitrate at Trail Valley Creek were determined using plant-root simulator (PRS®) probes (Western Ag Innovations Inc., Saskatoon, SK, Canada). Probes were installed next to each manual chamber flux collar and left in place for four weeks, after which the next set of probes was installed at the same location. We determined plant nutrient supply for three time periods (June, July, August) during 2019, as described in the Methods section. Nutrient turnover rates at Trail Valley Creek and Kilpisjärvi were determined via soil extractions before and after a four-week incubation at 4 °C, similar to the procedure described by Marushchak et al. (2021) and as described in the Methods section. Dissolved organic carbon (DOC) and dissolved TN (DTN) concentrations were determined on a TOC analyzer with TN measurement unit and autosampler (TOC-L, TNM-L, and ASI-L, Shimadzu, Kyoto, Japan).

Soil incubation experiments were conducted at 4 °C and 20 °C with soils from Trail Valley Creek (Upland tundra and Polygon rim) and Finnish Lapland (Upland forest and Palsa II). The first soil incubation used soils from both sites (homogenized soil, biological replicates in the same replication as for in situ flux measurements) in a simple temperature incubation. A second soil incubation experiment used soils from Finnish Lapland only (4 technical replicates for each land cover and vegetation type). During the second incubation, we incubated soils under the same temperatures mentioned above and additionally applied 2 different moisture regimes: 20% water-holding capacity and 60% water-holding capacity. In a third incubation experiment we used the same replication and soils as in the second incubation, but the incubation was done under field-moist conditions and labile carbon was added as glucose 1h and 24h into the incubation and compared to treatsments before carbon addition at 4 °C and 20 °C. Incubations are described in detail in the Supplementary Methods.

References:
Gaumont-Guay, D., Black, T. A., McCaughey, H., Barr, A. G., Krishnan, P., Jassal, R. S., & Nesic, Z. (2009). Soil CO2 efflux in contrasting boreal deciduous and coniferous stands and its contribution to the ecosystem carbon balance. Global Change Biology, 15(5), 1302–1319.
Lai, D. Y. F., Roulet, N. T., Humphreys, E. R., Moore, T. R., & Dalva, M. (2012). The effect of atmospheric turbulence and chamber deployment period on autochamber CO 2 and CH 4 flux measurements in an ombrotrophic peatland. Biogeosciences, 9(8), 3305–3322.
Marushchak, M. E., Kerttula, J., Diáková, K., Faguet, A., Gil, J., Grosse, G., Knoblauch, C., Lashchinskiy, N., Martikainen, P. J., Morgenstern, A., Nykamb, M., Ronkainen, J. G., Siljanen, H. M. P., van Delden, L., Voigt, C., Zimov, N., Zimov, S., & Biasi, C. (2021). Thawing Yedoma permafrost is a neglected nitrous oxide source. Nature Communications, 12(7107), 1–10. https://doi.org/10.1038/s41467-021-27386-2
Marushchak, M. E., Pitkamaki, A., Koponen, H., Biasi, C., Seppala, M., & Martikainen, P. J. (2011). Hot spots for nitrous oxide emissions found in different types of permafrost peatlands. Global Change Biology, 17(8), 2601–2614. https://doi.org/10.1111/j.1365-2486.2011.02442.x
Patrignani, A., & Ochsner, T. E. (2015). Canopeo: A powerful new tool for measuring fractional green canopy cover. Agronomy Journal, 107(6), 2312–2320.
Voigt, C., Lamprecht, R. E., Marushchak, M. E., Lind, S. E., Novakovskiy, A., Aurela, M., Martikainen, P. J., & Biasi, C. (2017). Warming of subarctic tundra increases emissions of all three important greenhouse gases - carbon dioxide, methane, and nitrous oxide. Global Change Biology, 23(8), 3121–3138. https://doi.org/10.1111/gcb.13563

Timing and spatial scale

Automated chamber and auxiliary measurements were conducted between June 21– August 24, 2019, and May 30 – August 31, 2021. Site access was not possible in 2020 due to travel restrictions in response to the COVID-19 pandemic. During the measurement periods, concentrations of CH4 and CO2 were recorded at 1 s intervals during a chamber closure of 3 minutes. All 18 chambers were measured once within an hour, resulting in hourly flux measurements. Manual chamber measurements at Trail Valley Creek were conducted 1–2 times per week between June 15 – August 30, 2019, and measurements were done once at all other sites (Scotty Creek: September 2018, Havikpak Creek: June 2021, Kilpisjärvi: August 2021). Manual chamber fluxes were measured during daytime (9:00–21:00, with the majority of flux measurements between 11:00–15:00).

Plant-available nitrogen at Trail Valley Creek was determined monthly during 2019 (June, July, August), and soil-extractable nutrients and DOC were determined once at Trail Valley Creek and Kilpisjärvi sites.

At Trail Valley Creek, the vegetation types measured with automated and manual chambers on upland tundra are located within a ca. 100 m radius from each other. The polygonal tundra site at Trail Valley Creek was ca. 2 km from the upland tundra site. The other two sites in the Western Canadian Arctic are located south of Trail Valley Creek: Havikpak is ca. 50 km south of Trail Valley Creek, and Scotty Creek ca. 900 km south of Trail Valley Creek. Kilpisjärvi is located in the European Arctic.

Data exclusions

Automated chamber flux data were cleaned as described in detail in the Methods and Supplementary Methods sections. After applying all data cleaning steps, 16 % of CO2 fluxes and 15 % of CH4 fluxes were discarded, resulting in a final dataset of 44 644 individual data points for CO2 and 44 848 measurement points for CH4 (sum of both measurement years). For manual chamber fluxes, filtering based on R2 and RMSE caused 1.1% of CH4 fluxes to be discarded. Data filtering criteria were based on previous studies (Järveoja et al., 2018; Marushchak et al., 2021; Voigt et al., 2017).

References:
Järveoja, J., Nilsson, M. B., Gažovič, M., Crill, P. M., & Peichl, M. (2018). Partitioning of the net CO 2 exchange using an automated chamber system reveals plant phenology as key control of production and respiration fluxes in a boreal peatland. Global Change Biology, 24(8), 3436–3451.
Marushchak, M. E., Kerttula, J., Diáková, K., Faguet, A., Gil, J., Grosse, G., Knoblauch, C., Lashchinskiy, N., Martikainen, P. J.,

	Morgenstern, A., Nykamb, M., Ronkainen, J. G., Siljanen, H. M. P., van Delden, L., Voigt, C., Zimov, N., Zimov, S., & Biasi, C. (2021). Thawing Yedoma permafrost is a neglected nitrous oxide source. Nature Communications, 12(7107), 1–10. https://doi.org/10.1038/s41467-021-27386-2<br>Voigt, C., Lamprecht, R. E., Marushchak, M. E., Lind, S. E., Novakovskiy, A., Aurela, M., Martikainen, P. J., & Biasi, C. (2017). Warming of subarctic tundra increases emissions of all three important greenhouse gases - carbon dioxide, methane, and nitrous oxide. Global Change Biology, 23(8), 3121–3138. https://doi.org/10.1111/gcb.13563
Reproducibility	Atmospheric CH4 uptake occurred consistently during both growing seasons at Trail Valley Creek, and were measured not only with the automated chamber system but were confirmed with further independent methods: manual chamber measurements and soil gas concentration measurements. Measurements with manual chambers confirmed the magnitude of CH4 uptake measured at Trail Valley Creek compared well to other well-drained sites in the northern circumpolar permafrost region. Measured uptake rates are also of the same magnitude as measured at other sites with portable greenhouse gas laser instruments (Hermesdorf et al., 2022; Juncher Jørgensen et al., 2015; Juutinen et al., 2022).<br><br>References:<br>Hermesdorf, L., Elberling, B., D'Imperio, L., Xu, W., Lambæk, A., & Ambus, P. L. (2022). Effects of fire on CO2, CH4, and N2O exchange in a well-drained Arctic heath ecosystem. Global Change Biology, 28(16), 4882–4899. https://doi.org/https://doi.org/10.1111/gcb.16222<br>Juncher Jørgensen, C., Lund Johansen, K. M., Westergaard-Nielsen, A., & Elberling, B. (2015). Net regional methane sink in High Arctic soils of northeast Greenland. Nature Geoscience, 8(1), 20–23.<br>Juutinen, S., Aurela, M., Tuovinen, J.-P., Ivakhov, V., Linkosalmi, M., Räsänen, A., Virtanen, T., Mikola, J., Nyman, J., Vähä, E., Loskutova, M., Makshtas, A., & Laurila, T. (2022). Variation in CO2 and CH4 fluxes among land cover types in heterogeneous Arctic tundra in northeastern Siberia. Biogeosciences, 19(13), 3151–3167. https://doi.org/10.5194/bg-19-3151-2022
Randomization	The measurement plots were selected in the field using the expertise of the field team to establish flux plots on the dominant vegetation and land cover types representative of the study region. Subjective selection was necessary to catch the important features in the studied landscape with a reasonable number of flux plots. Although the plot selection was not randomized, and thus prone to bias, for practical reasons this is a common plot selection method in chamber flux studies, where random plot selection would require a larger sample size than is feasible due to limited resources. The location of the flux plots, in particular the automated chamber plots, was constrained be their proximity to a power source and the autochamber system control unit: plots had to be within a ca. 35 m radius to the control unit and gas analyzer due to limited length of the inlet and outlet tubings between each chamber and the gas analyzer.
Blinding	Investigators were not blinded to group allocation during the data collection. Blinding was not possible since the same researchers who selected the study plots representing different vegetation types as described above carried out the data collection. The analytical and data processing methods were quantitative and as such insensitive for subjective interpretations.

Did the study involve field work? ☒ Yes ☐ No

Field work, collection and transport

Field conditions	All sites are located in the northern circumpolar permafrost region and extent from the continuous permafrost zone in the North to the sporadic permafrost zone in the South. Trail Valley is an upland tundra site located 45 km north of Inuvik, NT, in the western Canadian Arctic. The mean annual air temperature (MAAT) determined for Inuvik is -8.2 °C and mean annual precipitation (MAP) is 241 mm. Havikpak Creek is located 10 km south of Inuvik, NT, Scotty Creek is located 60 km south of Fort Simpson, NT (MAAT: -2.8 °C, MAP: 388 360 mm), and Kilpisjärvi is located in Finnish Lapland (MAAT: -1.9 °C, MAP 487 mm). Trail Valley Creek and Havikpak Creek are underlain by continuous permafrost, Scotty Creek is located in the discontinuous to sporadic permafrost zone where permafrost is mainly preserved in peat plateaus, and Kilpisjärvi is located in the sporadic permafrost zone. In Kilpisjärvi, permafrost has disappeared in the studied upland ecosystems (upland forest, upland tundra) but is preserved in the palsas.
Location	The locations of the study sites are as follows: Trail Valley Creek - 68°44'32" N, 133°29'55" W, 68 m a.s.l.; Havikpak Creek - 68°19'15" N, 133°31'05" W, 68 m a.s.l.; Scotty Creek - 61°18'29" N, 121°18'01" W, 169 m a.s.l.; Kilpisjärvi - 68°51'54" N, 21°06'24" E, 85 m a.s.l.
Access & import/export	The access to Trail Valley Creek and Havikpak Creek was organized by the University of Montreal in collaboration with Wilfrid Laurier University and facilitated by the Polar Continental Shelf Program. Research permits were administered by the Aurora Research Institute in Inuvik, Northwest Territories, Canada. Access to Scotty Creek was kindly permitted by the Dehcho First Nations and supported by Wilfrid Laurier University. Sample export was organized through the University of Montreal and University of Eastern Finland. Access to Kilpisjärvi was organized through the University of Eastern Finland and the research permit allowing measurements and soil sampling was administered by Metsähallitus.
Disturbance	The disturbance of the study sites was minimal and involved walking within the measurement plots and sampling of top soil as well as installation of small instrumentation. For repeated access to the flux plots as well as installation of the autochamber control unit, wooden boardwalks and a wooden platform were constructed at Trail Valley Creek to minimize disturbance.

Reporting for specific materials, systems and methods

We require information from authors about some types of materials, experimental systems and methods used in many studies. Here, indicate whether each material, system or method listed is relevant to your study. If you are not sure if a list item applies to your research, read the appropriate section before selecting a response.

Materials & experimental systems

n/a	Involved in the study
☒	☐ Antibodies
☒	☐ Eukaryotic cell lines
☒	☐ Palaeontology and archaeology
☒	☐ Animals and other organisms
☒	☐ Clinical data
☒	☐ Dual use research of concern

Methods

n/a	Involved in the study
☒	☐ ChIP-seq
☒	☐ Flow cytometry
☒	☐ MRI-based neuroimaging
